# Prescribing Generic Medication in Chronic Musculoskeletal Pain Patients: An Issue of Representations, Trust, and Experience in a Swiss Cohort

**DOI:** 10.1371/journal.pone.0134661

**Published:** 2015-08-03

**Authors:** Valérie Piguet, Stéphanie D’Incau, Marie Besson, Jules Desmeules, Christine Cedraschi

**Affiliations:** 1 Division of Clinical Pharmacology and Toxicology, Multidisciplinary Pain Center, Geneva University Hospitals and University of Geneva, Geneva, Switzerland; 2 Division of General Internal Medicine, Geneva University Hospitals, Geneva, Switzerland; 3 Division of General Medical Rehabilitation, Geneva University Hospitals and University of Geneva, Geneva, Switzerland; University of Washington, UNITED STATES

## Abstract

**Objective:**

Parallel to an ever stronger advocacy for the use of generics, various sources of information report concerns regarding substitution. The literature indicates that information regarding substitution is not univocal. The aim of this qualitative study was to explore patients’ representations regarding generics in patients suffering from non-specific disabling chronic musculoskeletal pain, as these patients are confronted with the issue of the prescription and/or substitution of original formulations with generics.

**Methods:**

Qualitative methods were selected because the aim was to access the range of patients' representations and to consider their conceptions. Standardized face-to-face semi-structured interviews were used, and transcripts were submitted to content analysis.

**Results:**

Patients’ representations suggest that they might be confident in taking a generic medication: when he/she has an understanding of generics as resulting from a development process that has become part of the public domain; the generic medication is prescribed by the physician; each prescription is discussed, i.e., the patient is prescribed the generic version of a given medication and not a generic medication.

**Discussion:**

Economic arguments are not sufficient to justify substitution, and may even raise issues calling upon cognitive dissonance. Even in non-life-threatening diseases, negative cues require attention and need be de-emphasized - in particular lower price as an indication of lower quality, and generic status as contradictory with advocating individualization of medication.

## Introduction

The prescription of generic drugs has been advocated as a less expensive alternative to brand-name drugs in an era of rising healthcare costs [1–3]. In the US and in European countries, the volume share of generics has increased since 2000 at varying rates, depending on market conditions and national regulations [4–5]. In Switzerland, a study on the determinants of generic substitution showed that higher rates were associated with higher out-of-pocket costs, greater price differences and the number of generics on the market [6]. In this country, physicians are not obliged to prescribe via the INN (International Non-proprietary Name). However, they must inform patients when a generic is available to replace an original drug. Pharmacists are legally allowed to replace a physician’s prescription of an original drug by a less costly generic. This substitution is always possible except when the physician notifies in writing that substitution is excluded for medical reasons.

Parallel to an ever stronger advocacy for the use of generics, a growing number of studies report concerns regarding medication substitution [7–9]. These observations have been reported in the media, raising mistrust of substitution in patients and health professionals and confusing the concept of safe substitution [7–8, 10–12].

Information regarding generic substitution is thus not univocal. This issue can be considered within the framework of the common-sense model of illness representations [13]. In this model, representations are the central cognitive construct that guides coping and the appraisal of action outcomes; these representations are conceptualized as highly individual and not necessarily in accord with medical or pharmacological facts. Developed as an extension of the common-sense model, the theoretical model of medication representations [14] is of particular interest. This model distinguishes between patients’ mental representations of whether they require medication (perceived necessity) and whether it will cause problems (perceived potential for concerns).

Non-specific musculoskeletal pain, a most common source of chronic pain and physical disability unrelated to life-threatening illnesses producing pain, is a frequent reason for patients to take prescribed and/or over-the-counter analgesic medication [15–19].

Scarce information is available about these patients with chronic musculoskeletal pain using generic analgesic medication. The aim of this study was to explore their views about generics, i.e., how they perceive them, their experience of generics, and how they feel about taking them. The use of generics has become an important topic, both in public health and economical terms. Using a qualitative interview study, we investigated the personal definitions and understanding of generics in patients suffering from non-specific and non life-threatening chronic musculoskeletal pain, to elucidate the reasons that might explain the reasons that patients are confident or reluctant to take generics. Our general hypothesis was that patients’ representations of generics would call upon the dimension of perceived potential for concerns more than upon the dimension of perceived necessity and confidence.

## Methods

### Study Design

Qualitative research methods were selected because the aim was to access the range of patients' representations and to consider their conceptions [20–21]. Standardized face-to-face semi-structured interviews were used [22].

### Participants

Participants referred to the Multidisciplinary Pain Center were recruited by telephone between January and October 2012. Patients were eligible and approached for participation if they presented with non-specific chronic pain of musculoskeletal origin and having a current and/or previous analgesic intake. Patients were not eligible if they had a diagnosis of tumor or inflammation-related pain. The type and number of participants were determined by the need to ensure a sampling typical of the chronic musculoskeletal pain patients attending the pain center. In qualitative studies, the number of participants is typically determined by purposive sampling, i.e., by the need to encompass the range of possible responses [23–24] and to achieve data saturation [25–27]. The participants are therefore selected according to predetermined criteria relevant to the research objective [28]. The determination of the number of participants required is based on data depth rather than frequencies, the sample should consist of participants as illustrative as possible of the study population [29–30]. In this study, the data collection essentially addressed the description of the type of analgesic medication used and the patients’ representations of the generics. Within a pragmatic and flexible approach to sampling [31], participants were included to ensure that the sample was diverse in terms of age, sex and experience and to provide additional insight based on data from the literature [25, 27, 32–33]. It was necessary that this sample include the various experiences and representations that individuals might have in relation to generics. A sample of 25 patients allowed reaching a point in the data collection and analysis at which new information produced no change in the codebook and the data were considered saturated [30].

### Data collection

The interviews were conducted at the Pain Center by a multidisciplinary team of three (VP, SI, CC) investigators (one clinical pharmacologist, one internist and one psychologist) not involved in clinical patient care. All three investigators are chronic pain providers and experienced in conducting qualitative interviews; the face-to-face interviews lasted approximately 45–60 minutes. The participants were questioned about their views on generic medications (Table 1). The interview sought to highlight the participants’ representations regarding generic medication and the conditions under which they have/would have accepted or refused such medication. It was responsive to the issues raised by the participants during the discussion so that the participants’ representations could be further explored whenever appropriate. The interviews were tape-recorded and transcribed.

**Table 1:** Semi-structured interview guide.

Topics	Examples of questions
Definition and contents of the term ‘generic’	According to you, what is a generic medication?
Prescription of generics	Has your physician ever prescribed generics to you?
Substitution of brand drugs by a pharmacist	Has the pharmacist ever proposed to substitute the brand drug with a generic one? What was your response?
Reasons to accept/hesitate to use generics	How would you feel about taking a generic drug? What would make you feel confident / hesitant to take a generic drug?

### Data analysis

The transcripts were analyzed using a manual data indexing technique to identify the key themes [34]. The qualitative content analysis began with individual readings by the three researchers, working separately. The analysis continued throughout the data collection and coding process, using the constant comparative method, which consists of analyzing the interviews by comparing one response with earlier observed responses [35]. This process was followed by a comparison of the readings, which were subsequently used to establish the analytical categories and dimensions. These categories and dimensions served as the basis for a final grid, which was used independently by the three researchers to analyze the transcripts to maximize the theoretical sensitivity and rigor [27,36]. The use of the patient-generated data via the interviews and validation by the three investigators of the data interpretation allowed for an assessment of trustworthiness [20]. This assessment entailed an analysis of the data carried out by a multidisciplinary group of investigators (VP, SI, CC) so that findings emerged from consensus between investigators]. As Barbour & Barbour [37] indicated, multidisciplinary research teams may raise challenges, but they allow data to be subjected to a range of ‘multidisciplinary gazes’ [38], drawing on a wider store of theoretical framework and insights, and “providing for a much more comprehensive and conceptually productive review than do traditional approaches based on triangulation with its restrictive focus on internal validation”. The matter at stake here may not be the degree of concordance between investigators but rather the insights that discussion can provide for refining coding frames [23]. Emergent findings were corroborated with existing theories and examined in comparison with previous research findings to assess the degree to which they were congruent with those of past studies. The demographic data as well as a reliable and validated measure of pain intensity (100 mm Visual Analogue Scale) and medication use data were collected using a questionnaire sent to the patients before the interview and the patients’ medical charts.

The protocol conformed to the Helsinki Declaration and was approved by the Ethics Committee of the Geneva University Hospitals; written informed consent was obtained from all the participants.

## Results

### Characteristics of the participants

Participants were recruited between January and October 2012. Twenty-eight participants were eligible. The final sample included 12 female and 13 male respondents and 3 patients (one male and two females) declined to participate because of time contingencies. Participants were aged 35 to 78 years presented with varying educational experience (Table 2). They suffered from non-specific disabling chronic musculoskeletal pain (Table 2). All subjects responded that they had taken some analgesic medication, and most (96%) reported being treated with at least one generic medication (S1 Table).

**Table 2:** Sociodemographic characteristics of the participants (S1 Table).

Characteristics		Participants
Age (y) Mean (SD) [range]		51 (15) [35–78]
Gender		
	Female	12
	Male	13
Educational status		
	Elementary school	8
	Qualified worker	8
	High school	5
	University	4
Employment status		
	Full time	1
	Part time	8
	Retired	1
	Unemployed	1
	Sick leave	8
	Disability pension	6
Pain etiology		
	Low back pain	10
	Fibromyalgia	4
	Other musculoskeletal	11
Pain duration (y) Median [range]		8.5 [1–50]
Pain intensity (VAS mm) Mean (SD)		66 (26)
Current analgesic intake		(n = 23, 92%)
	Acetaminophen	11 (48%)
	NSAIDs	12 (52%)
	Weak opioids	10 (43%)
	Strong opioids	8 (35%)
	Antidepressants	15 (65%)
	Anticonvulsivants	8 (35%)
	Muscle relaxants	5 (22%)
Current other medication intake		12 (48%)
Current generics intake		19 (76%)
Current generics analgesic intake		11 (44%)
Intake of generics ‘ever’		24 (96%)
Intake of generics analgesic ‘ever’		20 (80%)

### Participants’ representations of generic medication

The content analysis identified the following three dimensions: the identity, the issues related to the risks, and the costs of generic medications (S1 Text).

#### Identity of the generic medication

The first dimension referred to the similarities between the generic and the branded medication. At the beginning of the interview, the participants’ most frequent response echoed the biomedical definition in terms of an imitation of the branded medication with another name and a lower price. This response covered a two-fold perception of identity, i.e., the similarity and the differences or doubts regarding the similarity. Table 3 presents illustrative quotes from the participants’ responses.

**Table 3:** Dimensions of participants’ representations of generic medication: identity of the generic, issues related to the risks, and costs (S1 Text).

Dimension	Summary of main issues	Illustrative quotes
**Identity**		
Similarity		
	Generic and branded medication are similar in terms of substance, efficacy, adverse effects, and required controls	“It’s a cover version of a drug which has been marketed with a brand name (…) then this formula is taken over with the same dosages with maybe a different package or tablet…”[R12]
	Similarity can mean both types of medication lack efficacy, or are considered as dangerous	“Taking a drug is always a risk, whether it’s a generic or a normal medication, one always wonders about spots, patches or other problems” [R22]
Differences–doubts about similarity		
	Generics are not similar to branded medication, differences might concern the contents, appearance, manufacturer, name, price, and effects of the medication (positive and/or adverse)	“It’s a substitution drug but it’s not the same though because the components are different… otherwise they would have the same name (…) For me the [generic] has no effect while the [original] helps my pain” [R9]
	Generics are intended to be similar, but there are overt differences in the appearance, name and price and possibly also covert differences (e.g., the substance or the amount of the substance) raising doubts about similarity	“It’s the same but… they [the manufacturers] give the ingredients of the recipe, but not how to put them together (…) the order and how to proceed…” [R8]
	Notion of ‘copy’ as an issue	“It’s kind of a copy, rather a partial copy” [R15
**Risks**		
Related to the drug itself	Generics might have more or different adverse effects than the original drug	“It’s the same substance but combined with others. (…) The other substances can make adverse effects, in addition to those… [of the basic substance]” [R15]
	However, drugs could cause problems in general	“I’m scared of drugs [. . .] I check the information leaflet, and then I check the excipients on the Internet. . . [. . .] and I saw xx and no lactose. . . and I looked up for zz because I didn’t understand why my stomach was hurting and there it was: 'lactose'!” [R18]
Related to the prescription	Not (always) prescribed automatically	“One wonders why the physicians sometimes prescribe the brand name than at the pharmacy, they give you the choice and you wonder why (…) I’d be reassured if the physician would write it down immediately, but it’s also because there are several kinds of generic for a drug” [R21]
Related to the context of production	Questions are raised regarding the manufacturer, as well as the controls and regulations	“It’s the same stuff, normally… (…) it’s maybe 99% as efficient… difference of laboratory, chemical formula… not 100% the same but on the information leaflet it’s the same! They are less controlled; sometimes they are not at all controlled. Because sometimes you see products that come from far away or… It’s not the same… it's not 100% reliable… I take many medications and I have only the originals, from the French laboratories!” [R19]
**Costs**		
Positive aspects of generic medication		
	Less expensive because of the patent expiration and no need for research and development	“It’s an old drug that… after many years, is in the public domain… it’s a drug that has the same molecules, the same properties as the old one, but is cheaper because no research was needed… and the non-generic is more expensive because there was all the research work…” [R8]
	Less expensive and thus helps to reduce health care costs	“I’m a citizen concerned about the finances of the state… and mine as well, meaning also those of the healthcare insurances… I mean, why not take a medication that’s been proven efficient and less costly?” [R12]
Negative aspects		
	Less expensive because the quality is lower	“One can produce many more with machines that work much more rapidly, but the substance, there is less of it than in the old, more expensive ones” [R6]
	Less expensive; however, it is an obligation rather than a choice	“Well, it’s also the price that’s attractive uh. . . well… anyway it's not me who pays but the insurance gets nervous if you take the original…” [R11]

The concept of “copy” was very frequently mentioned, with various connotations that ranged from an “*identical copy*” to a “*forgery*” and included different forms of ‘copy (“*simultaneous translation*”, “*photocopy*”, “*partial copy*”, “*pale copy*”, and “*imitation*”). Using a copy might not be a problem in the cases in which the medications are identical and efficient, as follows: “*It is a healing molecule which has fallen into the public domain in its copyright*… *it is scientifically proven for its efficiency*… *(*…*) It’s an identical copy*, *there is absolutely no difference between the two*” [respondent 12]; it might not be a problem when the difference is only in the packaging: “*It’s like [low-cost products from a well-known superstore]*… *if you look at what's inside*, *it’s all the same*, *but there's the packaging looking like recycled*, *it is not for show*, *there’s nothing to attract customers’ attention*” [respondent 20].

It might become more of a problem when the copy indicates that the patient thinks he/she is receiving a second-choice medication: “*It’s a copy of the original product*… *the laboratory*… *they never give the exact formula*, *that's why it's a copy*… *(*…*) regarding the substance or*… *it’s not the first choice as they say*…” [respondent 19], instead of the best available medication: “*“It’s the same but… they [the manufacturers] give the ingredients of the recipe*, *but not how to put them together (…) the order and how to proceed…*” [respondent 8].

#### Issues related to the risks

The risks and fears related with generic medications were related to the drug itself, to its prescription or to the context of their production. Generics raised doubts about the quality control and regulations potentially resulting in weaker and more dangerous drugs than those for brand-name medications.

#### Costs

The third dimension referred to the costs, with generic medications being characterized as less expensive. This response was very frequent, with approximately 90% of the respondents referring to the costs; the response involved a two-fold representation of this issue, positive vs. negative (Table 3).

### Participants’ views about the prescription of and substitution by generics

When explicitly asked about the generics they might have been prescribed, i.e., when the prescription was placed in a personalized context, the majority of the respondents indicated that they had received a generic prescription and had discussed the substitution of the brand medication with a generic one or the shift from one generic medication to another one with the pharmacist.

Consistently with their current intake of generics, the patients emphasized the importance of trusting the prescriber and/or the dispenser- trust in the physician in one-half of the respondents and in the pharmacist in approximately one-fourth of them. Three respondents indicated that they had asked the physician for a prescription of a generic medication. Unconditional acceptance was described as being related to an inner conviction of the importance of generics to the reduction of healthcare costs or to a perceived absence of control over the medication, whether original or generic as follows: “*I don’t really care… I don’t know what’s in it anyway*, *so I don’t care how it’s labeled… (…) as long as it helps me*, *I don’t need to know more…*” [Respondent 24].

Hesitation to use generics was explicitly acknowledged in one-third of the participants and was predominantly related to their doubts about the similarity between brand and generics. Some respondents suggested that costs were the actual issue: “*My sister-in-law*, *she refuses all generics… she says as long as she can afford to pay for it*, *she wants to get the good one…*” [Respondent 17]. Hesitation or reluctance might have been more widely shared in our sample. Eight of the respondents expressed doubts about similarity as well as fears related to the generics while declaring that they would accept a prescription or a substitution. This contradiction was solved in different ways. Some of the patients explained that, although they would accept treatment with generics, the generic drug was not the best option in his/her specific situation (“*I’d try*, *but… I would try yes*, *but I’d keep the [original] in reserve*, *just in case…*” [Respondent 6]) or was an option that should be tried if his/her doctor considered it in the future.

## Discussion

The major finding of this study is that patients’ representation of generics is ambivalent; when one perception predominates, it is clearly a negative one. We found that patients question the similarity between brand and generic medication on a level significantly beyond the identity of the chemical substance. Patients emphasized their apprehension aspects related to generic medications, including the context of production, cost from a societal perspective and the risk of generic medication becoming mandatory rather than an option. Cost to the patient, to pharmaceutical companies and to society were indeed frequently referred to, and patients suspicion that generic medication may be inferior in quality to brand medication may be due to the premise that they are associating lower price when purchasing the medication at the pharmacy with lower production quality.

When addressing the issue of risks, the respondents either noted no difference between branded and generics (both can cause problems) or insisted on the risks of generics as possibly being higher. The risks were balanced with the efficacy: a number of respondents insisted on the inefficacy of the medication, presented as related to its very nature as a generic, i.e., to their doubts regarding the similarity between generic and branded medications.

This ambivalence calls upon Horne’s model of medication representations and the two constructs underlying medication-specific beliefs, i.e. perceived need for the medication and perceived potential for the medication to cause problems [14]. The issue here may well be that the perceived necessity is overwhelmed by the concerns regarding generics, thus resulting in an increased difficulty to feel comfortable with such a prescription. However, as Fairman [39] pointed out, why would we want more expensive drugs instead of engaging in an economically rational behavior? But engaging in such a behavior requires that lower cost is viewed as a positive attribute that connotes better value for the money spent. It has been suggested that paying a higher price for an analgesic drug leads to the perception of higher efficacy so that a higher cost or brand status increased the perceived value of a drug [40]. This in turn means that the perceived value of a drug is not only a matter of objectively cost-effective choice [39].

References to cost issues were another salient aspect of the findings of this study. The positive aspects of lower costs were generally perceived from society’s point of view with generics helping to decrease healthcare costs. The respondents described themselves as being concerned with a fair distribution of the benefits and costs between stakeholders. A representation of generics as efficient contributed to positively highlight the lower costs. Yet, reference was frequently made to the acceptance of generics as an obligation rather than a choice. Most of these respondents declared that they would agree with such an obligation to decrease healthcare costs; however, a number of them explained that in his/her specific situation, the regulation could/should not be applied as emphasized in the Shrank et al’s study [41]. As for a possible impact of patient’s insurance type and wealth on generic substitution, various studies have demonstrated that type of insurance and annual income influence generic substitution, with patients residing in high-income zip codes more likely to initiate treatment with a generic than patients in low-income regions [42;43]. Consistent with other studies, our results suggest that substitution contributes to patients’ anxiety and possible confusion. As reported by participants, since generic are not always initially prescribed, this raises doubts and some anxiety as to why one rather than the other may be prescribed [10, 44–45]. These findings were particularly relevant to patients who thought they had not received sufficient information and when provider’s explanations did not meet their expectations because substitution for a generic analgesic was not clearly discussed or cleared by the patient; these elements are likely related to how prescription of generic drugs is regulated in Switzerland [6].

Similar to our findings, the issue of cost was repeatedly mentioned, with a clear emphasis on the benefits of generic medications from a societal rather than an individual point of view. In a Finnish study, a majority of the participants approved the substitution, whereas a number of them concluded that only society benefited from this substitution [46]. A German survey showed that more than one-third of the respondents expressed general skepticism towards generics because of their lower price. This attitude was more frequent among those who felt that generic prescribing was "invented" to solve the financial crisis in the German health insurance system at the expense of patients [12]. This skepticism, related to generics as an instrument of cost containment, might include the idea of counterfeited drugs [47] that some of the respondents in our study referred to under the description of copy as a forgery. Substitution with generics might be in opposition to personalized medicine, a novel concept, emerging even in the media, in which developing medications according to the genetic profile of the patient is considered [48]. There is little existing evidence regarding the implementation of interventions aiming to inform the patients, including direct to consumers advertising [49]; however, efforts to empower patients to actively participate in the medication selection process may have the best effect on actual drug use [41].

The results of this study indicate that if patients consider generic medications as products that are no longer subjected to copyright, then these medications could be considered as ‘good’ and ‘usable’. However, if these medications are considered to be approximate second choice discount drugs, doubts and reluctance might be relevant and questions regarding generics as medications could arise; as a patient explains: “*Up to now*, *my doctors have never prescribed me generics*, *they’ve always prescribed me medications*” [Respondent 10].

Regarding contradictory opinions about generic prescription, it has been suggested that the tension between cost and value raises cognitive dissonance [50]. Dissonance is a negative drive state occurring when an individual simultaneously holds two cognitions which are psychologically inconsistent. To seek consistency between their expectations and their reality, individuals engage in dissonance reduction to bring in line cognitions and actions [51]. When inconsistencies are present, the dissonance can result in restoring consonance through misperceptions, rejection or refutation of the information, or through validation of one’s behavior [52]. These attempts to reduce dissonance lead the patient to justify the decision he/she has made by focusing on the positive features of the chosen alternative and the negative features of the rejected one. Such a strategy implies to make the two options subjectively less similar and the chosen alternative much better than the rejected one [52]. As this respondent indicates: “*The xx*, *which is called the Rolls-Royce in this field*, *is much more effective than the generic I had before… (…) If a medication has been invented and it’s satisfactory*, *I don’t believe an imitation can be as good as the real one*, *the original*” [respondent 16]. In such a model, positive cues for the generics should be identified and emphasized in order to make for a consistent choice.

The results of this study demonstrate the importance of negotiation regarding the prescription of generics. Patients might feel confident taking a generic medication in the following situations: when they have a representation of generics as the result of a R&D process that has become part of the public domain; when generics are prescribed and the information is provided by the physician (and supported by the pharmacist); when each prescription is discussed and negotiated regarding a generic or an original formulation, i.e., the patient is prescribed the generic of a given medication and not an ‘unidentified’ generic. Patients might feel reluctant to accept, or refuse, a prescription and/or a substitution: when they have a representation of generics as second choice discount drugs; when the prescription is perceived as unclear or the substitution as unexpected; and when the prescription or substitution is regarded as related to the demands of cost.

There are limitations to this study. The results provide an insight into the perception of a specific group of patients with non-specific disabling chronic musculoskeletal pain, most of whom suffered long-term pain. As in all qualitative studies, our study sample was small, and although the data showed variations, content analysis allowed reaching theoretical saturation. Transferability might be limited to clinical settings addressing patients suffering from chronic pain. Furthermore, this study was conducted in a Swiss setting, which is different from those of some other countries regarding the prescription, dispensation and reimbursement of generics. The importance of the setting was stressed in a US nation-wide study investigating generic substitution in various states implementing diverse policies in Medicaid patients; i.e., either mandatory or permissive substitution by the pharmacist with or without patient consent required for substitution [53]. It is noteworthy that the context in Switzerland parallels that of US states with permissive substitution policy after patient consent. In their study, Shrank et al observed that with patient consent requirement, such policy resulted in a 25% reduction in generic substitution [53]. Thus, substitution appears to be related more to the patient choice than to the regulations [53], therefore emphasizing the importance of considering patient representations.

Information, trust and experience are important aspects for patient adherence to a prescription for a generic medication; economic arguments *per se* are not sufficient motivation. The results of this study provide ample support for generic medication raising many concerns and much lesser perceived need. As noted above, the perceived value of a drug is not only a matter of objectively cost-effective choice [39]: the price means a cost to the patient—so that when the price increases, purchasing decreases; however, the price may also mean quality–so that when the price increases, purchasing increases as well. Cognitive dissonance theory predicts that those patients who have paid a higher price for their medication will then value it highly to avoid dissonance. Thus, those aspects of generic medications raising concerns need be addressed. At the same time, sources of cognitive dissonance also need to be considered so that positive cues can be stressed and negative cues can be de-emphasized—in particular lower price as an indication of lower quality, and generic status as contradictory with advocating individualization of medication.

## Supporting Information

S1 Text Full transcript of the interviews. (DOCX)

S1 Table Socio-demographic, pain and medication intake characteristics. (DOCX)
