# Supplementary material for: Prescribing Generic Medication in Chronic Musculoskeletal Pain Patients: An Issue of Representations, Trust, and Experience in a Swiss Cohort
Source: PLoS One. 2015 Aug 3;10(8):e0134661. doi: 10.1371/journal.pone.0134661 (PMC4523195; doi:10.1371/journal.pone.0134661)
Supplement: S1 Text — (DOCX) [file pone.0134661.s001.docx]

**Prescribing generic medication in chronic musculoskeletal pain patients: an issue of representations, trust, and experience in a Swiss cohort**

**S1_Text : Full transcript of the interviews of the 25 patients**

**Patient 1**

(Donc, euh, je vais vous demander à votre avis qu’est-ce-que c’est un générique ?)

Oh, c’est une sorte de copie de l’original par définition presque. C’est à dire qu’il y a un laboratoire qui a fait des études pendant des années et des années, des tests, euh le met sur le marché pendant un certain nombre d’années et ensuite euh, au bout d’un certain nombre d’années, le brevet tombe ce qui veut dire que euh d’autres sociétés, d’autres firmes peuvent fabriquer le même produit euh à moindre coût puisqu’ il y a pas les études, les tests euh qui ont été faits préalablement

( Vous pensez qu’ils ont la même efficacité ?)

oui

(oui d’accord. La même sécurité aussi ? les effets indésirables, les effets secondaires)

Euh moi j’ai le sentiment oui si ce n’est qu’au niveau sécurité ça dépend un petit peu de la manière dont il est conditionné

(Mmh)

C’est-à-dire que euh j’ai remarqué que suivant le type de de euh pastille que je prends

(ouais)

y en a certaines les génériques notamment qui sont plus difficiles à avaler peut-être

(d’accord)

et donc là il faut faire un petit attention euh à pas avoir de petit problème de coincement de euh (ouais) pastille au niveau de la gorge (ouais) car là c’est un petit peu pénible (d’accord) c’est la seule chose que j ‘ai pu remarquée

(d’accord donc vous avez déjà eu une expérience avec des génériques ?)

très peu

(très peu d’accord. Vous souvenez euh quel type euh quelle substance ?)

Alors pour l’instant donc comme je vous ai dit tout à l’heure je prends pas d’antalgique (ouais) mais je prends de euh de l’inhibiteur de la pompe à protons

(exactement) donc euh avant c’etait du Nexium que je prenais maintenant c’est de l’ omezol (d’accord) alors on m’a dit que c‘ était pas tout à fait la même molécule mais en fait le but c’était à peu près le même, moi je vois pas de différence

le conditionnement est différent euh je dirais même que euh la capsule qui est incluse dans l’omezol est plus facilement ingérable donc là c’est l’effet inverse

(d’accord ok mais sur l’effet vous avez pas remarqué de différence ?)

Non non il est tout à fait efficace

(d’accord ok et puis vous les tolérez aussi bien l’un que l’autre ?)

Oui oui

(d’accord et puis pourquoi vous avez changé ?)

… euh je pense que déjà au niveau, on m’a pas obligé en tout cas il y a pas eu de pression pour ce médicament-là, c’est euh oui je crois à priori c’est pour le dosage, comme je suis un adepte du microdosage le moindre dosage possible, le Nexium faisait un dosage à 20 mg bon est-ce qu’on peut comparer je sais pas vu que c’est pas la même molécule (oui) tandis que l’omezol a des conditionnements à 10

(ah d’accord)

voilà c’est donc tout simplement ça et il est deux fois moins cher

(ouais ouais et vous parce-que vous avez décidé que 20 mg c’était trop pour vous ou bien c’ est vous qui décidiez, qui aviez envie d’avoir un dosage plus faible ?)

Disons moins je prends de médicament mieux je me porte (ouais) c’est ce que je me dis (d’accord) donc c’ est sur cette base-là (d’accord d’accord ok) que je fais mon choix et aussi par rapport au coût c’est clair que si l’autre médicament celui qui avait un conditionnement avec le plus petit un plus petit dosage euh j’hésiterais si il coûtait deux fois plus cher quand même (d’accord) il y a quand même (d’accord) une petite conscience des coûts de la santé un petit peu quand même

(ouais ouais d’accord ok euh vous savez donc puisque vous l’avez dit qu’un médicament euh doit être autorisé à être mis sur le marché est-ce que vous pensez que les les ils sont soumis aux mêmes contrôles un générique que la molécule mère ?)

Alors je sais pas honnêtement je sais pas parce-que je me dis c’est quand on met un médicament sur le marché que le contrôle est fait (mmh mmh) euh si c’est un générique et qu’il s’agit exactement de la même molécule j’ai le sentiment que y a pas du tout de contrôle en fait (qu’on le laisse comme ça ?) mouais si c’est la même molécule (d’accord) par contre si c’est une molécule légèrement différente (oui) là je pense que oui rebelotte pour les contrôles j’imagine

(ouais ouais d’accord donc vous avez l’idée que c’est moins contrôlé les génériques ? on pourrait résumer comme ça ou ?)

Disons que comme ils ont été contrôlés préalablement lors de la mise sur le marché du médicament original y a quand même un contrôle mais en amont y a bien des années

Qu’est-ce qui vous feriez hésiter à prendre un générique ?

(d’accord ok d’accord ok qu’est-ce qui vous feriez hésiter à prendre un générique ?)

…………………

(par exemple si je reviens au traitement que vous avez vous avez de l’inderal hein il existe un générique est-ce que si votre médecin demain vous propose le générique vous allez le prendre sans autre ou est-ce que ?)

Euh je sais pas si c’est la même chose mais j’avais pris du beloc à un moment donné (mmh mmh) Et puis euh il semblerait que l’interaction est pas tout à fait la même avec le corps tout ça et il semblerait que l’inderal ait un effet sur les vaisseaux eux-mêmes, sur les varices et sur la possibilité de développement de ces varices donc euh si c’est exactement exactement le même produit et c’est là mon inquiétude en fait (d’accord) et c’est la question que je poserais au médecin (d’accord) si c’est exactement le même produit ça me pose aucun problème de changer (ouais ouais ) mais faut vraiment qu’on me dise c’est la même molécule avec euh la même terminologie euh biochimique si vous voulez

(ouais alors c’est le propranolol dans l’inderal on appelle ça euh la molécule le nom international c’est le propranolol et il existe du propranolol euh générique le Beloc zokc’est pas c’est autre chose) ok (c’est le metoprolol voilà euh donc euh si le medecin vous dit ça est-ce que vous ?)

Si c’est exactement la même molécule j’insiste c’est pas euh y a pas un petit peu euh euh ça me pose aucun problème

Comment en avez-vous entendu parler ?

(d’accord ok euh comment vous avez entendu parler des génériques parce-que vous êtes bien au courant euh euh comment vous avez entendu parler des génériques ?)

Oh c’est lors de la polémique euh qui a commencé à surgir il a quelques années sur les coûts euh des médicaments eux-mêmes pas seulement les coûts de la santé mais particulièrement les coûts des médicaments euh on été tous choqués il y a 15 ans 20 ans en arrière de savoir que le même médicament était vendu euh 2 fois ou 3 fois moins cher à l’étranger en France tout simplement alors qu’en suisse on payait le maximum donc euh on enfin moi je me suis dit euh qu’il y avait quelque part quand même un problème est-ce que la Suisse seule doit payer toutes les études pour ce médicament et pas le monde entier sous prétexte euh il y a les lois du marché qui s’appliquent et que donc euh c’est à nous de payer respectivement les caisses maladie et à ce moment là il y a eu un petit problème une prise de conscience plutôt politique alors à ce moment là si y a d’autres entreprises qui viennent se faufiler qui disent euh euh bah qu’on a des génériques qui coutent 2 fois moins cher bah y a pas de problème

Alors évidemment je comprends très bien que euh y a les y a toutes les études qui ont été faites pour mettre sur pieds ce médicament euh qu’il faut assumer mais pourquoi seul le consommateur suisse (d’accord) et en plus je sais pas si y a une TVA sur les médicaments mais si y en a une elle est plus basse que euh dans certains autres pays j’imagine alors je m’explique pas qu’un médicament produit en Suisse donc on paye pas le voyage on paye pas le transfert tout ça euh soit payé plus cher que en France pour exactement la même chose

(d’accord d’accord euh mais là c’est pas forcément les génériques en France par exemple l’inderal il est vendu tel prix en Suisse et le même inderal avec le même nom de marque est vendu moins cher)

Oui c’est ça que je voulais dire je sais pas si me suis mal exprimé, c’est en comparant exactement le même nom le même produit fabriqué par la même firme le prix n’est pas le même et la justification a souvent été faite oui mais les capacités euh financières de chaque pays ces capacités-là sont pas identiques alors pourquoi est-ce qu’en Allemagne c’est moins cher quand même qu‘on m’explique un petit peu voilà

(d’accord et c’est en vous intéressant à ça que vous avez découvert les génériques ?)

Ouais

(d’accord ok euh est-ce que vous êtes allé chercher par exemple des informations sur internet ?)

oui ,oui oui

(oui alors ça ça m’intéresse beaucoup comment vous faites quand vous allez chercher une information sur internet ?)

J’y vais pas souvent ça m’est arrivé une fois ou deux c’est très simple euh: google, génériques de inderal (d’accord ok vous l’avez fait pour) oui oui oui et puis je sais que euh je dois avoir quelque part dans mes favoris euh un site qui parle des génériques (d’accord) qui est consacré uniquement à ça ou en partie à ça donc on fait le lien entre les deux (oui) le seul souci comme je vous ai dit tout à l’heure c’est le fait d’avoir euh exactement la même molécule si elle marche avant pour euh l’ original j’aimerais bien que ce soit la même molécule

(ouais ouais d’accord euh si le médecin vous dit que c’est la même molécule ça vous suffit ou bien vous allez quand même voir euh sur internet ?)

quand même voir sur internet (rires)

(ok d’accord et puis par exemple pour l’omezol vous êtes allé voir euh sur votre site des génériques ?)

oui et c’est pas tout à fait la même chose d’après mon souvenir hein c’est pas tout à fait la même molécule et je me suis dit bon y a ce 10 mg moi j’ai été un peu simple dans mon raisonnement (oui) donc y a moins de substance c’est moins concentré c’est que 10 mg je vais quand même essayé quitte à revenir sur le Nexium je m’étais attaché à ce petit Nexium (ouais) parce-que efficace et puis ça marche aussi

(ouai c’est vrai c’est pas l’un est esomeprazol et l’autre omeprazol) voilà c’est ça (mais c’est un changement minime et en fait ils ont la même efficacité voilà)

Votre médecin vous en a-t-il déjà parlé ou prescrit?

(euh quand votre médecin vous a prescrit l’omezol par exemple qu’est-ce qu’il vous a dit euh ?)

… Alors le euh c’etait de savoir si il y avait une concentration un peu moindre c’était la question initiale il a regardé sur son Compendium et puis euh tout d’un coup il a pensé peut-être à un un il a dit j’ai entendu parlé d’un générique puis il a regardé sur le Compendium euh concernant le générique il y a du 10 mg pour ça et puis euh on a ouais ça a remporté la mise si je puis dire

(ouais d’accord ok euh donc vous avez tout de suite accepté quand euh ouais d’accord)

ouais avec un petit contrôle quand même avant et euh ouais on va quand même essayé

Votre pharmacien vous a-t-il déjà proposé de substituer le médicament original pour un générique ?

(ouais d’accord ok et est-ce que votre pharmacien vous a déjà proposé une fois de substituer un médicament pour un générique alors je sais pas moi pour un antibiotique ou pour un autre médicament qui vous serez venu ?)

Alors euh sur le principe jamais sauf une fois assez récemment comme je vous ai dis aussi hier euh pour le beloc vu que c’est un origine euh c’est pas un générique c’est un original euh on a juste attiré mon attention sur le fait que y a ces 20% de franchise ou de quote part qui il faut payer euh et puis voilà simplement sur ce plan là uniquement sur un plan financier vu que c’était à ma charge mais sinon cette pharmacie là n’est pas pas très active sur ce plan (d’accord)

(Et puis vous vous avez décidé à ce moment là de garder ce que le médecin avait prescrit et de payer 20% votre quote part euh à la place de 10 ?)

Oui oui tout à fait même si il y avait une petite euh petite inconnue à ce niveau là vu que j’atteins de toutes façons la quote part maximale qui est prévue dans la loi est-ce que quand même je dois payer en plus de cette quote part maximale ce 20 % là bon en tout cas la caisse maladie me l’a appliqué mais c’était 1 fr 50 euh donc euh j’allais pas faire des histoires pour 1 fr 50 non j’avais quand même garder ce générique-là pour une raison simple c’est que j’ai des problèmes au niveau des yeux c’est-à-dire que j’ai les yeux qui ont tendance à être secs à s’infecter etc donc je prends des gouttes et on s’était demandé si l’inderal ne provoquait pas ça vu que ça fait parti de la longue liste des effets secondaires et puis euh en prenant le beloc euh d’abord j’ai pris j’ai rien pris pendant 1 mois et j’ai pas vu de grands changements alors euh alors voilà ça a peut-être pas euh amélioré grand chose avec le beloc je l’ai quand pris euh pour cette raison là mais j’ai vu aussi dans les effets secondaires qu’il y avait un problème au niveau euh aussi oculaire, sécheresse oculaire ça change rien mais je l’ai quand même pris pendant un mois (d’accord) ça faisait pas de différence je suis revenu à l’inderal

(ouais si dans une comme ça en général euh dans la prescription les médecins peuvent écrire euh euh inderal et demander que vous l’ayez pour des raisons médicales à ce moment-là le pharmacien ne peut pas changer euh si le médecin écrit inderal sans spécifier euh le pharmacien peut vous proposer de changer pour le générique et puis le médecin peut aussi écrire euh propranolol par exemple et à ce moment-là le pharmacien choisit le euh nom de marque qui est le meilleur marché est-ce que vous euh de n’avoir pas convenu sur un nom avec le médecin est quelque chose de déstabilisant dans le sens si le médecin écrit bon je met propranolol et le pharmacien verra ce qu’il vous donne ou je met inderal mais le pharmacien pourrait vous proposer autre chose ou met inderal mais oublie de vous dire que le pharmacien pourrait vous proposer autre chose est-ce que vous à ce moment-là euh le médicament il y a un petit flottement euh c’est moins euh ?)

Si j’ai la certitude c’est la même molécule il y a plus de flottement donc le flottement réside vraiment là-dessus sur le principe le fait d’avoir un générique euh une sorte de pâle copie moi le mot pâle je l’enlève une copie pour moi si c’est une copie tout-à-fait fidèle pour moi ça me va

(d’accord et pour vous pour être rassuré sur le fait que c’est la même molécule euh il faut que le médecin vous le dise il faut que vous l’ayez aussi lu quelque part il faut que le pharmacien vous le dise il faut avoir une, deux sources pour être euh)

oui (oui d’accord ok)

(d’accord euh alors on va passer à la deuxième partie à moins qu’il y ait quelque chose d’autre que vous vouliez dire en général sur les génériques ?)

Oh je suis juste peut-être un cas particulier parce-que je vérifie euh tout bien les choses je me méfie beaucoup des des paroles comme ça je crois que la plus pâle des écritures euh est toujours plus forte que la plus forte des paroles et donc si c’est écrit quelque part sur un site officiel ou bien dans le Compendium euh je l’ai pas mais euh ma compagne l’a un ancien Compendium des années 2000 je me dis ça peut-être euh une source si on veut là ça me suffit (vous savez que le Compendium vous pouvez l’avoir online) oui oui (y a un site et) c’est juste (comme ça vous pouvez tout à fait vérifier et moi je suis je comprends tout à fait je pense que deux sources dont une écrite est beaucoup mieux)

(ok alors est-ce que vous avez une expérience personnelle avec des génériques antalgiques ? est-ce que par exemple vous avez pris une fois ?)

Oui souvent du paracétamol (voilà) alors avant c’était le panadol (voilà ça c’est la marque primaire) voilà et ensuite euh euh vu que j’en prends plus j’avais pris une grosse boîte de générique de panadol en tout cas c’était du paracétamol (ouais) et c’est très connu mais j’ai un blanc (je sais pas Dafalgan ?) oui Dafalgan voilà c‘était du Dafalgan (d’accord) ça allait très bien aussi (donc même effet ?) oui oui enfin ça a pas un effet euh antalgique euh pour des douleurs extrêmes mais si on prend suffisamment tôt d’après ce qu’on m’a dit ça ça marche (d’accord) oui oui c’est très bien

(et qui vous avait proposé le Dafalgan plutôt que le panadol dont vous aviez l’habitude ?) moi

(c’est vous d’accord de nouveau dans le souci de ?) là c’etait plus politique qu’autre chose je dirais ouais là je dirais que le panadol euh je me suis dit mais il font de la pub ça a l’air d’être très commercial tout ça euh euh pas pour le panadol lui-même mais pour le euh dolo ou je sais pas quoi ils ajoutent une petite substance avec et il y avait de la pub à la télévision pour ce médicament (dolospedifen ou quelque chose comme ça ?) y avait panadol plus un truc qui donnait un effet endormant (avec la codéine en fait ?) avec la codéine absolument oui il faisait la pub là-dessus là je me suis dit oui ça commence euh (ah d’accord c’était la même boîte pharmaceutique que le panadol) absolument c’était panadol avec un petit plus devant (ah d’accord peut-être panadol plus) je crois qu’ils n’ ont pas le droit de faire de pub si ça ne l’est pas par la caisse maladie alors ils font de la pub indirecte en ajoutant la substance à côté mais bon panadol avec le même logo je trouve un peu bon (d’accord donc c’est ça qui vous a fait aller vers) oh déjà peut-être un petit peu pour être franc avant mais euh ça ça a disons a fortifié mon idée que euh là on essaie de tirer un maximum de profits sur une marque euh qui a été rentable pendant quelques années et qui l’est moins maintenant

(d’accord euh ) ….

donc là c’était moi qui avait demandé euh au médecin (d’accord) donc euh si ça posait un problème pour lui non je met le Dafalgan

(d’accord ok et donc les effets antalgiques vous avez dit que c’étaient les mêmes et les effets indésirables vous avez pas remarqué d’autres effets indésirables une différence ?)

non

(d’accord ok là on a parlé éventuellement pour le futur du Tramal que vous avez eu une fois je crois euh) oui oui tout à fait

(euh il existe donc le nom international du Tramal c’est le tramadol et il existe différentes euh maintenant plusieurs génériques du tramadol donc euh ) avec la même molécule ? (avec la même molécule oui donc euh toujours il faut savoir qu’ils sont tenus de mettre la même molécule pour qu’ils soient acceptés comme génériques par contre ce qui peut changer c’est les excipients les colorants bah vous avez remarqué que les comprimés sont pas les mêmes) oui tout à fait

(donc ça ça peut changer mais pour être générique on doit avoir la même molécule et toutes ces molécules ont un nom international et le nom international pour le Tramal c’est le tramadol donc vous pouvez vérifier sur les boîtes hein en regardant le nom international il doit figurer voilà donc par exemple inderal c’est propranolol il sera sur toutes les boîtes ce sera marqué propranolol et ils n’ont pas le droit de marquer propranolol si ce n’est pas du propranolol dedans c’est un peu comme quand on va acheter du chocolat on regarde ce qu’ il y a comme composants c’est la même chose voilà ) d’accord

(donc pour vous si votre médecin vous propose un générique du tramadol vous seriez d’ accord de faire l’essai ?) oui oui

(d’accord)

c’est vous m’aviez parlé l’autre hier de de gouttes (oui tout à fait ) donc c’est clair que si euh le générique a aussi des gouttes euh qui permettent de doser euh de manière assez fine la même chose il y a pas de problème c’est ça c’est ça un petit peu aussi le euh confort d’avoir un petit peu aussi le euh la maîtrise sur le dosage et plus j’ai une maîtrise mieux c’est (alors ça je peux pas encore vous répondre savoir si il y des gouttes et comment elles sont dosées il faut que je regarde oui mais je vous proposerez le tramadol et puis en fonction des des galéniques des formes ) vous avez dit que euh donc tramadol vous dites mais c’est pas une marque déposée ? (non non la marque déposée qui était la première c’ était Tramal il ont fait une sorte d’abréviation du tramadol le composé la substance qui est active s’appelle le tramadol et la marque déposée s’appelle le Tramal et très souvent maintenant les génériques gardent le nom de de euh la molécule très souvent maintenant les génériques c’est tramadol et le nom de la firme après, mepha peut-être vous avez déjà vu ça euh pour le paracétamol je sais pas vous qui avez fait la liste y a avait ça ?) omezol c’est la substance (oui omeprazole c’est ) ah ils ont fait une contraction (oui ils ont fait une contraction aussi oui mais ils ont ajouté mepha donc mais c’est vrai que ça peut vous indiquer que c’est un générique quand il y a le nom de la firme à côté)

(voilà donc je vous remercie beaucoup on va arrêter)

**Patient 2**

(On va commencer de manière très générale et ma première question est de vous demander ce que à votre avis est un générique ?)

Ce qu’est un générique ?

(Ouais à votre avis c’est quoi un médicament générique ? Qu’est–ce que ça veut dire pour vous ?)

Pour moi un médicament générique c’est la reprise de formule d’un médicament qui a été mis sur le marche par une compagnie avec un nom d’une marque parce que la formule a été inventée par et ça a été repris par un fabricant alors y a en différents qui reprennent cette formule-là et qui refont le médicament aux dosages similaires avec peut-être un contenu, je dirai un emballage différent avec une enveloppe au niveau du médicament un panadol qui devient avec un autre emballage aussi au niveau du comprimé qui diffère c’est-à-dire aussi qu’ ils ont pas les coûts ce qui fait qu’ il est moins cher c’est qu’ il y pas les coûts de recherche de la substance

(Les coûts de recherche ?)

Bah je veux dire si euh je sais pas y a roche fait un médicament va le développer y a des coûts de recherche et développement qu’un générique a pas c’est aussi ça qui fait baisser les coûts parce que c’est un médicament qui existe depuis un moment donc qui est repris

(Qui est repris alors c’est la même substance de votre point de vu qu’il y a dans le médicament?)

Alors pour moi ouais c’est la même substance

(C’est la même substance)

Alors peut être avec des adjuvants et des choses peut-être différentes mais la substance médicamenteuse est la même

(Donc ça voudrait dire est-ce qu’il a les mêmes effets indésirables ou est-ce qu’il en a d'autres, est-ce qu’il en a plus, il en a moins ?)

Alors si on prend je sais pas peut-être que je me trompe mais si on prend que le dosage du médicament lui-même je pense que ca change pas grand chose mais après tout ce qui il y a dans le paquetage de la fabrication du comprimé il peut y avoir des choses différentes donc peut-être il peut y avoir des effets indésirables chez l’un et pas chez l’autre

(Et alors est-ce que vous diriez qu’il y a les mêmes contrôles administratifs, gouvernementaux sur les génériques que sur le médicament d’origine ?)

Alors exactement les mêmes je pense pas car quand un médicament est mis sur le marché au tout début il y a des tests cliniques qui se font donc je pense que le générique n’a pas besoin de faire puisqu’ ils ont déjà été faits par rapport a la substance donc ils gagnent aussi du temps donc il y a une nette réduction des coûts

(D’accord donc en somme ce serait la même chose à part la présentation ce qu’il y a autour de la substance j’ai bien compris ?)

Bah il y a toute la il y a tout le côté de la recherche et développement qu’il y a pas après au niveau de la finalisation du médicament je pense que l’effet souhaité par le produit pharmaceutique c’est le même

(C’est le même d’accord alors quand vous dites l’emballage enfin pas la boîte le le) je sais pas comment on appelle (très franchement moi non plus) l’enrobage

(Voilà on va appeler ca l’enrobage l’enrobage est différent alors vos diriez que ça change quelque chose au niveau de la prise du médicament ?)

…..alors par rapport à mon expérience alors moi je suis infirmier alors quand j’étais en pédiatrie et puis que je j’étais on était au début quand on commençait à avoir différents paquets par rapport au paracétamol y avait des fois on avait des contrats avec différentes boîtes on a eu le panadol, Dafalgan ça a changé et suivant la présentation du médicament les enfants avaient plus ou moins de peine à le prendre alors ça on remarquait (de peine à le prendre ?) pour l’avaler par rapport au goût par rapport à plein de choses donc si la forme est moins enrobée moins lisse car y a en a qui ont un enrobage plus lisse donc plus faciles à avaler si il est plus ovale avec moins des arrêtes des choses comme ça et puis au niveau psychologique aussi c’est plus difficile je pense qu’il y a un tout qui fait parce que le fait d’avaler quelque chose quand on voit certains qui avalent 20 comprimés d’un coup je pense le capacité d’avaler on la mais après je pense qu’au niveau psychologique il y a pas mal de choses donc après il y a tout un lien qui se fait

(D’accord donc ce ce serait pas forcement lié aux génériques mais ce serait lié à la manière d’enrober ce qu’on a appelé l’enrobage vous et moi finalement que ce soit un générique ou l’original l’enrobage compte c’est ça que vous dites)

Moi je pense ouais l’expérience fait que oui

(D’accord c’est pas spécifiquement les génériques qui auraient un problème à ce niveau là ou quelque chose de spécial ?)

non mais je pense qu’un générique il va peut être moins mettre d’argent dans l’enrobage parce que ça a un coût la recherche par rapport à ça, des couleurs, de la forme peut être que d’avoir un moule alors je sais pas comment c'est exactement parce que j’ai pas été voir mais je pense qu’il doit avoir surement des moules et des façons de pouvoir les créer donc peut être que si ils ont un moule standard ils vont tout faire sur le même et vont peut être pas mettre un spécifique pour chaque substance différente dans le sens ou c’est aussi une réduction donc c’est un tout

Qu’est-ce qui vous ferait hésiter à prendre un générique ?

(Vous même est ce que vous hésiteriez à prendre un générique ?)

Non parce que chaque fois qu’un médecin me prescrit je vais demander au pharmacien qu’il me donne un générique si ca pas été fait d’office

(Vous vous demandez d’office donc vous n’hésiteriez en aucun cas à prendre des génériques ?)

Non

(Y a pas des choses qui vous feraient hésiter ?)

Non

Comment en avez-vous entendu parler ?

(Comment est-ce que vous avez entendu parler des génériques ?)

Bah dans ma formation

(D’accord donc c’est professionnel ?) Voilà

(Vous avez été professionnellement amené à être en contact avec l’existence des médicaments génériques) Mmh Mmh (d’accord)

(est-ce que ça vous est arrivé d’aller chercher des informations à propos des médicaments génériques ?)

………..Après en discussion parce-que mon épouse travaille en pharmacie bon elle est pas pharmacienne puisqu’elle était assistante en pharmacie à l’époque donc elle était assez euh à rencontrer tous ces différents emballages qui arrivaient par rapport au prix par rapport à tout donc on a toujours pu en discuter

(donc vous êtes très largement informé ?)

Bah je pense que j’ai une connaissance euh une bonne connaissance

(oui ça vous est arrivé d’aller voir dans les médias je pense par exemple à internet ça vous est arrivé d’aller chercher des informations sur les médicaments génériques ou sur d’autres médicaments ?)

Bon dernièrement pas car je travaille plus en milieu hospitalier donc j’ai moins à administrer des médicaments donc je l’ai pas fait

(et pour vous-même ?)

J’ai pas je ressens pas le besoin d’avoir plus d’informations

(d’accord si vous alliez chercher des informations c’est quel genre d’informations que vous iriez chercher ?) Par rapport à ça ? (oui)

……………………………………………………….oh ça serait euh bah pour mon intérêt professionnel ça serait l’expérience des gens de savoir bah voilà comment ils vivent de prendre un générique puisque j’ai affaire à des gens donc c’est peut être ça qui m’intéresserait de savoir bah comme vous faites aujourd’hui de savoir euh comment eux le vivent de prendre un générique donc ça peut être euh par rapport donc moi je travaille avec des enfants donc c’est peut être différent mais on pourrait regarder suivant la population suivant la culture les tranches d’âge parce que je pense peut être les personnes âgées qui a eu l’habitude de prendre pendant des années le même aura peut être plus de difficulté à changer ça c’est quand même quelque chose de reconnu quand même dans les pharmacies de proposer un générique plutôt que de prendre le Brufen habituel : alors d’aller chercher ces informations je suis pas sûr que j’irai les chercher parce-que je pense que je les vis au quotidien donc euh je pense donc c’est peut être plus interessant de l’avoir des personnes directement plutôt que sur des forums ou autre oû il y a un peu tout et n’ importe quoi et on retrouve les mêmes choses qui sont mises et remises

(donc vous ce qui vous intéresserait de savoir c’est ce que les gens pensent des génériques )

Mmh Mmh (de manière un peu générale donc là on sort un peu de l’entretien c’est quoi votre intuition par rapport aux génériques ?)

………………………..je pense ça dépend de la catégorie socio-professionnelle socio- économique je pense que des personnes d’un milieu socio-économique moyen/supérieure a suffisamment d’information pour prendre du recul par rapport à ça et pas se faire embarquer dans tout le nappage publicitaire qu’il y a autour je pense qu’on arrive à le faire et peut être que des personnes en tout cas moi c’est ce que je vois sur le terrain des personnes qui accèdent à un certain niveau de moyens économiques mais qui est pas très haut ils sont toujours assez friands de tout ce qui est justement reconnu au niveau de l’image donc c’est des personnes assez sensibles à ça je pense

(donc ils seraient moins susceptibles d’accepter les génériques ?)

Je pense ouais peut être par manque d’information mais aussi par rapport à l’image que ça donne je fais toujours le parallèle avec l’alimentation dans l’alimentation c’est assez flagrant on préfère toujours prendre une marque chez certaines catégories de personnes

(d’accord parce qu’on pense que c’est mieux ?)

Alors ils peuvent penser ça mais c’est pas forcément parce que c’est mieux mais par rapport à l’image qu’on donne aux autres de pouvoir s’offrir le produit de marque ça donne une image plus positive par rapport aux autres alors est ce que sur les médicaments ça fait la même chose c’est peut être un produit de consommation moins visible que l’alimentation mais je pense que y a des personnes qui peuvent réfléchir moi je préfère prendre ça car c’est une grande boîte qui l’a fait qui l’a mis sur le marché on voit à la télévision

(d’accord effectivement ça semble tout à fait vraisemblable)

Votre médecin vous en a-t-il déjà parlé ou prescrit?

(Et vous-même votre médecin vous a-t-il prescrit des génériques ? Il vous a déjà parlé des génériques ?)

…………….Bon malgré les apparences je vais rarement chez le médecin ……donc j’ai eu mon médecin que j’ai eu je l’avais depuis que je suis gamin et le médecin qui a repris c’est Mme C là qui a repris le cabinet qui a à peu près mon âge je pense donc c’est une autre génération donc le vieux médecin lui il travaille avec des habitudes donc c’est pour ça que moi j’étais toujours à dire je veux des génériques

(Oui c’est pour ça je me disais c’était en somme vous qui lui en aviez parlé ?)

non je pense qu’il était au courant mais les gens souvent ils ont des habitudes et puis moi connaissant plusieurs médecins après il y a aussi dans les cabinets des fois un petit peu le côté mercantile qui arrive bah parce que voilà il y a des réalités de terrain avec les représentants et puis c’est comme ça et peut être pas tous les gens le voient et puis bah tous les médecins le font pas mais il y en a certains et puis bah le médecin actuel qui a une autre formation qui est plus jeune bah elle travaille vraiment avec les génériques

(Ok donc elle vous prescrit des génériques donc en général vous acceptez plutôt ?)

Bah oui

(Est-ce que vous avez un exemple précis, est-ce que vous .. ? euh qu’est-ce que elle vous dit votre médecin quand elle vous prescrit un générique ?)

Bah elle me dit rien parce que je pense qu’elle sait que je suis au courant

(Donc elle vous donne pas d’informations particulières ?)

Non parce que on sait l’un et l'autre qu’on est en terrain connu donc il y pas tellement de discussion autour de ça

Votre pharmacien vous a-t-il déjà proposé de substituer le médicament original pour un générique ?

(Est-ce que c’est arrivé que votre pharmacien vous propose de substituer le médicament original par un générique ?)

Alors certaines pharmacies oui et puis dans d’autres non ça c’est de nouveau. Je n’ai pas un échantillonnage suffisamment grand

(Non non mais quand on vous l’a proposé quand le pharmacien vous a proposé cette substitution de l’original par le générique est-ce que vous vous rappelez e qu’on vous a dit pour le justifier ?)

…………………….alors j’avouerai que c’est pas la majorité des cas parce que peut être eux aussi ils ont plus de marge sur un non générique les marges sont très différentes donc ils ont peut être pas non plus l’avantage même si ils devraient le faire par éthique mais ils le font pas de manière systématique il y en a certains qui le font mais mon expérience était plus parce que je connaissais peut être mieux j’étais client plus régulier ou ………..Parce que moi je le demandais ou je l’avais déjà demandé à la prescription je pense pas que j'ai assez de recul pour pouvoir répondre de manière claire

(Donc vous avez pas de souvenir précis de choses qu’on vous aurait dites très clairement)

Non alors peut être le prix peut être c’était ça par rapport au prix mais

(Il coûte un peu moins cher que l’original)

C’est ce qu’on aurait pu me dire mais j’ai pas de souvenir frappant

(De choses qu’on aurait pu vous dire)

Non

(Mais vous avez accepté la substitution)

De toutes façons j’ai pas trop été confronté à ça car je l’ai demandé au médecin

(Vous la demandiez vous même d’accord)

(Dites qu’est ce que vous prenez actuellement contre la douleur ?)

Rien

(Vous prenez rien du tout)

Non comme traitement médicamenteux aucun

(Rien du tout)

Non j’en ai essayé plusieurs mais maintenant j’ai arrêté parce que ça faisait pas d’effet

Enfin y a des effets mais pas suffisamment positifs par rapport aux effets secondaires

(Donc vous prenez plus rien depuis combien de temps ?)

Ouf bah j’ai fait des essais parce que là j’ai eu un épisode de douleur qui a recommencé cet été donc euh…. Le médecin m’avait proposé un nouveau pour moi mais qui était pas forcément nouveau mais que je connaissais pas qui était le zaldiar et comme je supporte peu les morphiniques je l’ai essayé deux fois et puis je l’ai pas pris

(Vous l’avez pris 2 jours) non je l’ai pris deux fois même pas deux jours parce que l’impression que j’étais plus moi même et que …….. Je préférais gérer la douleur autrement que d’avoir ces effets

(Donc là c’était votre dernier essai en somme est-ce que vous avez essayé d’autres médicaments ?)

Bah les anti inflammatoires euh Brufen et consort j'ai essayé ceux de marques pas ceux de marque…

(C’est la question que j’allais vous poser donc il y a des génériques pour vôtres traitement ?)

Bah entre les paracétamol et les euh c’est comment ibuprofène bah j'en ai essayé différents les effets étaient pour moi similaires

(C’était les mêmes effets ?)

Mmh mmh

(Les mêmes effets positifs les mêmes effets secondaires ?)

Alors ceux là ils me font pas d’effets secondaires donc j’ai pas pu les

(Pas d’effets secondaires)

Ils ont pas forcément d’effets sur cette douleur là pour d’autre chose oui je me suis cassé le doigt il y a deux semaines donc j’ai pu prendre euh anti-inflammatoires et paracétamol et là j’ai vu que ca faisait effet mais ça me fait pas d’effet secondaire j’ai pas de problème gastrique ou autre ça ça me fait rien

(Alors là vous avez pris un générique ?)

Oui j’ai pris un générique

(D’accord et c’est vous qui, ça vous a semblé normal qu’on vous propose un générique ?)

…………….quand ils m’ont fait l’ordonnance j’étais à Martigny et c’est une jeune assistante qui m’a fait l’ordonnance

(Alors ça c’est passé comment ?)

………Quand elle m’a prescrit ?

(Ouais)

Bah elle a fait sa prescription et puis il y avait déjà d’office dessus euh…..si je m’en rappelle bien le générique

(Donc c’est quelques chose dont vous aviez déjà l’habitude vous avez eu ni à demander ni)

Dans ce cas là non

(Non d’accord donc ça donc vous avez en somme une expérience personnelle avec les génériques antalgiques qui est déjà existante hein avec les anti inflammatoires, le paracétamol et c’est des médicaments que le médecin vous a déjà proposés en somme ?)

Oui

(ça vous a surpris qu’elle vous propose immédiatement un générique ?)

Non je trouve ça normal

(Et qu’est ce qui a fait que vous avez accepté qu’on vous propose un générique?)

…………..bah de nouveau pour moi c’est une évidence

(Donc c’était une prescription immédiatement de générique il y a pas eu besoin de changer de négocier ou quoique ce soit ?)

Non

(D’accord et vous avez pas eu d’effets indésirables ?) Non (et vous avez eu un effet bénéfique ?) oui (qui est celui que vous auriez attendu) de ce médicament-là

(D’accord c’est juste que pour votre douleur l’autre) celle que j’ai en chronique pour cette douleur là le paracétamol ou l’ibuprofène fait pas d’effet donc (d’accord et le zaldiar non plus ?)

Alors le zaldiar si sur la douleur mais ça me fait trop d’effets secondaires de somnolence de nausées qui fait que

(Donc c’est les effets secondaires qui font que)

Que j’ai pas envie de la prendre parce que ça me permet pas de fonctionner de manière correcte dans ma vie euh de tous les jours

(Donc là on est plus tellement dans générique/original si on vous avait prescrit le générique ça aurait été)

Oh non ça aurait été la même chose alors je sais pas si il existe ça fait un moment que je suis plus sur le terrain alors le zaldiar je savais pas si il existait un générique ou pas mais je cois que c’était une boîte de je sais plus quelle fabrique de générique blanc avec le

(Oui mais donc finalement c’est plus le problème du patient de savoir si ça existe ou pas en générique ?)

……………………………. (C’est plutôt celui du médecin ou du pharmacien ?)

Je pense que c’est une conscience de tout le monde parce que je veux dire euh……………dans ce cas là c’était un accident pour le doigt donc c’est l’assurance accident qui prend en charge donc j’ai pas de franchise donc à la limite je m’en fiche de savoir que ça va coûter plus ou moins cher après c’est une conscience personnelle de dire bah voilà euh je préfère prendre un générique par ce que au niveau des coûts de la santé ça va être moindre

(je vous pose la question parce que pas tellement en terme de conscience mais parce que finalement vous disiez que vous saviez pas tellement si il existait ou non un générique du zaldiar finalement le patient est pas forcément au courant)

Alors après il y a une connaissance des choses ouais

(Vous vous êtes particulièrement informé de par votre profession et de par la profession de votre femme mais un patient lambda)

Ah non il va aller avec son ordonnance et puis prendre ce que on lui a prescrit sur l’ordonnance ça c’est clair dans la majorité des cas je pense

(D’accord donc vous si on vous dit qu’il y a des patients qui refusent de prendre le générique parce qu’ils pensent que c’est pas assez fort ça vous surprend)

Non parce qu’il y a une méconnaissance et des habitudes, peur du nouveau donc ça m’étonne pas

(Ce qui est pas votre cas à l’évidence)

Non parce que j’ai suffisamment d’informations qui font que ça peut me rassurer

(C’est vrai qu’est ce qui de votre point de vue pourrait être inquiétant pour ces gens là ?)

………………………………………c’est ce que je disais avant ils vont peut être associer à une copie et puis je sais pas euh………….dans d’autres domaines les copies………souvent on peut, il y a des choses qui sont pas conformes, je sais pas il y a une émission à bon entendeur pour ceux qui ont regardé sur les cartouches d’encre de marques et de substitution en quelque sorte qui coutent moins cher car ce sont des copies et les résultats sont pas aussi bons dans les tests alors peut être les gens peuvent faire aussi des amalgames par rapport à d’autres domaines de consommation et peuvent dire on le retranscrit sur les médicament en ce disant c’est une copie de médicament et on peut ne pas avoir confiance

(C’est vrai on entend toujours parler de montre ou de chaussures de sport copiées alors ce serait la même chose mais de moins bonne qualité en somme ce serait la confusion entre la copie et l’idée du générique ?)

Mmh mmh je pense qu’il y a une grande confusion par rapport à ça pour certaines personnes

(Vous avez tout à fait raison c’est une piste très intéressante tout à fait)

**Patient 3**

(Je vais commencer par une question générale, à votre avis qu’est-ce que c’est pour vous un générique ?)

Un générique c’est un médicament que c’est moins cher que les autres mais pour moi il fait les mêmes effets que les autres médicaments

(Pour vous c’est la même substance ?) Oui pour moi c’est la même chose

(D’accord et c’est aussi efficace que l’autre médicament ?) Oui pour moi c’est efficace comme l’autre médicament

(ça fait les mêmes effets indésirables ?)Oui (vous l’avez expérimenté ?)Oui (d’accord et vous avez eu les mêmes effets et les mêmes effets indésirables ?)Oui ils sont moins cher c’est tout

(D’accord vous pensez que pour les mettre sur le marché c’est les mêmes contrôles que pour l’original ?)Oui c’est les mêmes contrôles (donc vous avez autant confiance pour l’original que pour le générique ?) oui oui (d’accord ça vous pose pas de problème) non non

(Vous avez l’impression que les génériques ils ont plus difficiles à avaler, à prendre ?) Pour moi non même si c’est des capsules ou des comprimés pour moi c’est bon (vous arrivez à tous les prendre mais vous avez pas remarqué qu’avec un générique vous auriez eu plus de difficultés ?) non pour moi non

(Vous savez qui fabrique les génériques ?) Non (et puis l’original ?)………………………non (non vous regardez pas les ?) non je regarde mais je fixe pas dans la mémoire mais je regarde tout le temps à chaque fois que j’ai des médicaments je regarde tout le temps mais je fixe pas dans la mémoire

Qu’est-ce qui vous feriez hésiter à prendre un générique ?

(D’accord donc est-ce que vous vous hésiteriez à prendre un générique ?) Tous les médicaments qui a un générique je le prends plus que les autres je vais le prendre je vais changer avec l’autre je prends plus de génériques que de l’autre (donc vous vous regardez toujours qu’il y ai le générique et vous prenez le générique ? donc vous hésitez pas ?) ah non j’hésite pas (donc vous avez pas de méfiance, de questionnement ?) non non même la première fois qu’il m’a posé la question

Comment en avez-vous entendu parler ?

(D’accord qui c’est qui la première fois vous en a parlé)

Ah c’est le docteur M

(Pour quel médicament vous vous souvenez ?)

Ah c’est le premier médicament oh maintenant je sais pas j’ai pris beaucoup de médicaments je me rappelle même pas mais c’est un médicament il a dit « c’est moins cher tu veux essayer » moi j’ai pris je me sentais bien et pas de différence avec les autres

(Donc vous en avez entendu parlé par le docteur M la première fois est-ce que avant vous en aviez déjà entendu parlé ?) Non, non non (c’était la première fois) c’était la première fois c’est pour ça après moi j’ai demandé pour les autres moi j’ai dit bon si c’est comme ça si c’est le même effet si ça va pour moi je le prends c’était il y a 5 ans

(il y a 5 ans ok est-ce que à part ce que vous a dit le docteur M vous avez cherché d’autres informations sur les génériques ? je sais pas au pharmacien par exemple ?)

Votre pharmacien vous a-t-il déjà proposé de substituer le médicament original pour un générique ?

non moi j’ai demandé au pharmacien pour ce que je prends maintenant pour l’estomac il a dit non non pour celui-là il y pas il faut prendre celui-là moi j’ai dit c’est bizarre après quand je suis de nouveau passée chez le médecin il a dit pourquoi tu prends pas celui-là ça c’est très cher j’ai dit moi j’ai demandé à la pharmacie qui me dit non il y a pas et après je demande de nouveau après trois semaines et elle me dit excusez moi je pensais pas qu’il y avait et après elle me le donne c’est pour ça que moi je demande

(Autrement vous avez demandé au pharmacien des renseignements sur les génériques ?)

ah oui les différences qu'il y a entre les autres et les génériques il y dit que c’est la même chose et un jour moi j’étais là j’attendais pour prendre les médicaments avec une ordonnance et j’écoutais une personne âgée qui avait déjà 88 ans par là et la pharmacienne lui dit « écoutez pour celui-là il y a un générique ; ah non non non moi je veux pas de toutes façons c’est l’assurance qui paie moi je préfère les autres et c’est pas la même chose le générique il fait pas le même effet c’est une grande différence » et après moi j'ai demandé et elle dit non non c’est écrit comme ça moi j’ai dit je le sais je le prends mais des fois il faut demander de nouveau

(D’accord donc vous avez redemandé à la pharmacienne parce que ce que a dit la patiente ça vous avait un peu…) oui je me dit pourquoi elle dit que c'est pas et elle elle me « dit non non elle a ça dans la tête et c’est comme ça »

(ca vous a un peu déstabilisée ?) Bien sûr ça c’est normal

(D’accord vous aviez lu quelque chose dans les journaux ou à la télé, entendu parlé comme ça)

La télé j’ai écouté parler des génériques

(Vous vous souvenez ce qu'ils avaient dit à la télé ?)

non moi je regarde pas beaucoup la télé parce que je reste pas beaucoup assise mais j’ai vu une fois je sais qu’ils parlent beaucoup des fois des génériques dans le télé. J’écoutais parler ils ont dit « si vous pouvez prendre un médicament qui coûte 100 francs mais le générique qui coûte seulement 20 pourquoi la différence ils ont le même effet » bon j’ai dit ça c’est vrai elle a raison « elle dit que l’assurance elle paie pas le médicament c’est cher pourquoi pas prendre le générique » moi j’ai dit d’accord mais j’ai pas beaucoup écouté la première fois oui j’ai écouté pour savoir tous les effets des génériques.

(Donc votre médecin le docteur M était le premier à vous prescrire un générique après les autres médecins prescrivent les génériques ?)

C’est tout le temps le docteur M et le docteur O

(Et vous avez toujours accepté quand ils vous prescrivaient ?)

Toujours j’accepte tout le temps j’ai une chance il dise madame vous prenez le générique je le fais tout de suite j’écris même pour ne pas oublier des fois elle dit non la pharmacie c’est ça que je comprends pas

(Parce que vous vous avez eu l’expérience que vous êtes allé avec l’ordonnance et le pharmacien ne vous a pas donné le générique ?)

Non c’est vrai j’ai amené le médicament il coutait 200 peut être 230 francs il est là c’est celui-là il a dit qu’il y avait pas (le Nexium) il m’a donné l’autre mais celui-là c’est très cher et 3 semaines seulement il m’a donné un autre pour trois mois et c’est un médicament qui est pas cher

(D’accord c’est l’omezol ?)

Je crois que c’est ça

J’ai trouvé bizarre moi j’ai dit je vous ai demandé combien de fois ? Ah non non il y a pas il m’a dit il y pas de génériques et maintenant vous me donnez ça c’est pas normal (et qu’est ce qu’ils vous ont dit ?) « j’ai pas fait attention » mais j’ai dit il faut faire attention moi si je vois ça me fait le même effet et c’est moins cher je le prends, ça me fait le même effet pour les douleurs moi je prends aussi pour la tension le générique et ça va bien c’est pour ça moi je trouvais bizarre la pharmacie

(Mais c’est la seule fois ou il ont pas donné le générique ?)

Oui oui

(J’ai vu que vous prenez du Dafalgan pour la douleur là il y beaucoup de génériques)

Ah bon (ça on vous a pas dit ?) non non (alors je sais que maintenant il y a pas une grande différence de prix entre le générique et l’original mais le Dafalgan n’est pas l’original l’original c’est le panadol) j’ai pris aussi beaucoup de temps mais après on m’a dit peut-être c’est mieux pour toi le Dafalgan c’est pour ça je le prends maintenant (pourquoi on vous a dit de prendre plutôt le Dafalgan ?)

Elle a dit le panadol c’est plus pour les douleurs de la tête ou des choses comme ça et l’autre le panadol aussi mais il fait aussi pour des douleurs aussi normales

(Parce que il y a d’autres génériques pour le Dafalgan il faut voir avec le pharmacien lequel est meilleur marché)

(J’ai vu que vous avez pris du voltarene aussi et là aussi il y a des génériques on vous les a proposés ?)

Ah oui voltarene aussi mais maintenant je le prends plus maintenant c’est juste la morphine, la cymbalta pour la tension et le temgesic qui me fait monter la tension ou l’apranax

(Alors là le temgesic il n’y a pas de génériques le transtec non plus la cymbalta non plus et l’apranax c’est un genre de voltarene)

(Donc en fait vous dès que les médecins vous ont proposé un générique vous étiez déjà convaincue que c’était une bonne chose)

Oui depuis que je l’ai pris la première fois

(Qu’est-ce que vous avez pensé la première fois ?)

la première fois je me suis dit la douleur est tellement forte je vais voir (vous aviez peur de quoi) de souffrir plus (d’accord que ce soit pas efficace) voilà j’ai dit bon je vais essayer je me sentais bien alors je me suis dit chaque fois qu'il y aura un générique je le prendrai (et quand il vous a prescrit le générique pour la tension là aussi vous étiez tout de suite rassurée) ah oui je comprends maintenant que c’est la même chose que les autres alors il faut éviter les choses qui sont plus chères

(si je disais que le docteur M il vous a prescrit la première fois le générique il vous a dit que c’était la même chose mais vous vous étiez pas tout à fait sure jusqu’à ce que vous fassiez vous l’expérience c’est juste ?)

oui oui je fais confiance à lui mais pour mes douleurs c’est ça c’est pas pour autre chose je veux pas souffrir plus mais je lui fais confiance

(donc ce qui vous a vraiment convaincue c’est d’avoir vous fait l’expérience que sur la douleur c’était la même chose )

voilà oui c’est ça

(Vous lui faisiez confiance sur le fait qu’il y a pas plus d’effets indésirables que c’est pas dangereux mais pour l’effet il fallait que vous voyiez)

Voilà c’est ça, c’est pas bien de parler comme ça mais c’est la vérité

(Non non c’est bien de savoir ça, c’est très intéressant et vous lisez les notices d’emballages ?)

Oui chaque fois mais je fais pas grande attention des fois je vais lire après parce que si je vais lire avant peut être je dit ah c’est vrai ça va faire tous ces effets, il y en a un j’espère qu’elle va me le changer pour autre chose

(Lequel j’ai pas compris) (le temgesic) oui ça c’est terrible ça fait transpirer et des petits boutons et je parle n’importe quoi et je sursaute (d’accord la dose est un peu forte peut être) mais c’est mieux pour les douleurs (et ça vous aviez lu la notice d’emballage) oui (d’accord et vous lisez aussi la notice d’emballage des génériques)ah oui je lis tout c’est pour ça je trouve ça bizarre qu'il y a pas celui-là et celui-là non plus des génériques je comprends pas car c’est très très cher chaque fois que je vais prendre c’est 3 boites c’est neuf cent francs et les autres c’est des génériques pourquoi il y a pas

(vous savez pourquoi on met un générique sur le marché ?)non (parce que on peut pas mettre tout de suite un générique sur le marché parce que la firme pharmaceutique qui a inventé le transtec elle a un brevet qui protège le médicament pendant 10 ans donc on peut pas faire le générique pendant 10 ans c’est pour ça vous trouvez pas le générique)ah voilà c’est pour ça (mais peut être dans une année ou deux il y aura)c’est mieux qu’ils mettent le générique (mais il y a pas encore) c’est pour ça qu’elle veut pas me le mettre (voilà elle peut pas vous le mettre encore en générique car ça n’existe pas encore )

(Il y des choses que vous voulez ajouter)

Je suis contente avec le générique oui c’est vrai qu’ils mettent plus de médicaments génériques car c’est autant efficace que les autres

(Dans votre famille tout le monde prend des génériques ?)

C’est seulement moi qui prends des médicaments

**Patient 4**

(Vous savez ce que c’est un générique ?)

Non

(Quand on vous prescrit un médicament on vous prescrit un nom de marque et il y a des médicaments avec le nom de marque et il y a des médicaments qui sont moins chers. Les médicaments quand ils sont mis sur le marché c’est une firme pharmaceutique qui fait ça et elle donne un nom à ce médicament qui est protégé pendant 10 ans après il y a des gens qui peuvent faire des copies et les copies sont en général moins chères. Peut-être vous avez eu par l’assurance ce médicament ils veulent pas le rembourser parce que il y en a des moins chers sur le marché ça vous est arrivé ?)

Oui

(Vous vous souvenez pour quel médicament ils vous ont dit ça ?)

C’était le glucophage et puis après j‘en ai parlé avec le médecin et il m’a donné le metformine

(Alors la metformine c’est le générique qui c’est qui vous a dit ça c’est l’assurance ?)

Oui l’assurance

(Alors vous en avez parlé avec le médecin qui vous a proposé la metformine et qu’est-ce qu’il vous a dit le médecin quand il vous a proposé la metformine, vous vous souvenez ?)

Non

(D’accord et vous vous vous êtes posé des questions vous vous êtes dit j’ai envie j’ai pas envie de prendre la metformine ?)

Non non j’en ai parlé au médecin il a dit je vais vous mettre ça et j’ai dit c’est bon quand même il a dit oui c’est bon quand même

(D’accord le médecin vous a dit que c’était la même chose ou bien vous vous souvenez ?)

Non il a dit que c’était bon aussi

(Et vous vous avez remarqué que c’était bon aussi ?)

J’ai pas trouvé de différence parce que même si j’ai diabétique moi je sais pas vous dire que j’étais malade de cette maladie moi j’ai jamais vu des de euh des (des signes ?) des signes

(Donc en fait vous avez pas pu vérifier vraiment si le générique metformine était aussi bon que le glucophage ?)

…….non

(Mais vous avez fait confiance au médecin ?)

Mais le taux de diabète c’est toujours à peu près le même c’est ça que on peut dire que c’était sur ça parce que quand je prenais le glucophage et on faisait le test ça revient 8.5-9 avec le metformine ça fait deux ans que je prends ça, ça restait environ la même chose

(Donc en fait vous faites autant confiance à la metformine que au glucophage ?)

Non moi je fais confiance au médecin (au médecin d’accord ça c’est bien mais pour vous vous savez que les médicaments ils ont un effet bénéfique il fait baisser le glucose dans le sang mais parfois ils peuvent avoir des effets indésirables les médicaments des effets secondaires ennuyeux est-ce que vous avez eu des effets ennuyeux avec la metformine ?)

Ou je sais pas si c’était ça j’ai eu comment dire des fois j’ai eu euh de l’acidité mais à l’époque je prenais le 1000 le plus fort et j’en ai parlé à le médecin et on baissé à le 800, 850

(850 effectivement et ça va mieux avec ça ?)

Après j’ai plus eu de problème non après j’ai eu mal à la gorge mais c’était pas ça c’était les les euh……..ah je sais pas comment ça on dit (une angine ?) l’angine

(Et vous pensez que la metformine et le glucophage ont les mêmes effets indésirables ennuyeux ?)

Non je crois que non

(Qu’est-ce que vous pensez que il y en a un qui est mieux que l’autre par rapport aux effets ennuyeux ?)

……………………….moi j’ai pas trouvé de différences et puis j’ai confiance au médecin parce que moi je suis je euh je prends pas de médicaments même pas un Dafalgan pour le mal de tête etc je prends le médicament quand ils me disent les médecins autrement je prends rien

(D’accord est-ce que la metformine était plus difficile à avaler que le glucophage ?)

Non

(C’était le même comprimé, la même grosseur ?)

Je crois que c’était plus petit

(C’était pus petit donc c’était plus facile même à avaler ?)

C’est pareil pour moi ou petit ou grand

(Pour vous vous hésitez pas si le médecin vous prescrit un générique vous le prenez c’est pas un problème ?)

Si il dit le médecin oui

(D’accord si c’est le pharmacien qui vous propose de changer ? par exemple le médecin il fait une prescription et le pharmacien il voit que dans sa pharmacie il a un générique moins cher est-ce que vous êtes d’accord de prendre sur l’avis du pharmacien ?)

Non (ok c’est clair)

Déjà je vous dit je prends pas je prends seulement si il me dit le médecin

(Ok est-ce qu’il vous est arrivé de chercher de l’information sur les médicaments est-ce que vous lisez la notice ?)

Oui je lis des fois

(Pour la metformine vous avez lu la notice ?)

…..oui (et le glucophage aussi ?)

…..oui mais je me rappelle plus (oui bien sûr c’est tellement compliqué, d’accord mais il y a pas quand vous avez lu la notice de la metformine des choses vous vous êtes dit tiens bizarre ?)

…………………..moi des fois je vais contrôler les effets contraires et puis…………quand c’est un médicament je prends toujours je ne lis plus

(D’accord ça vous arrive d’aller sur internet chercher des informations sur les médicaments ?)

C’est pas pour moi (c’est pas pour vous l’internet)

Même pas l’ordi quand j’ai besoin de quelque chose j’ai deux enfants et ils font eux

(C’est eux qui vont sur internet ?)

Oui

(Donc vous prenez aussi par exemple du felden ?)

Mais pas tout le temps vraiment c’est quand j’arrive pas à résister quand je le prends je le prends c’est le médecin qui m’a dit je le prends pour 4-5 jours et puis après j’arrête (oui c’est juste) parce que autrement il m’a dit qu’il faudrait la protection pour l’estomac (tout à fait) ça je le prends pas encore je veux pas rajouter encore un autre médicament parce que je prends déjà tellement ça fait………3 le matin et 4 le soir

(ça vous est arrivé de prendre d’autres médicaments contre la douleur le médecin vous a prescrit parfois d’autres médicaments que le felden ?)

Oui c’est le coDafalgan qu’il m’a prescrit une autre fois (d’accord et il y a d’autres choses ?)

Non

(Non d’accord) quelques sirops pour la toux pendant 10 jours ou 2 semaines (donc le felden quelques jours seulement et puis ....)

Je prends cipralex et zyprexa le soir, cipralex matin et zyprexa le soir, le matin j’ai pris janumet et aspirine cardio et soir janumet et sortis ça c’est ce que je prends tous les jours depuis des années

(D’accord donc vous avez pas tous les jours un médicament contre la douleur ?)

Non quand j’ai trop mal je rajoute le felden ou le coDafalgan

(Le Dafalgan ça vous arrive d’en prendre ?)

Non je ne l’ai jamais pris

(Le médecin vous a jamais prescrit ça ?)

Non

(Ou un autre le tylenol ou des choses comme ça ?)

Non

(Alors à l’heure actuelle vous avez pas de générique pour la douleur)

………………….je sais pas (je vous le dit)

J’ai dit ce que je prends d’habitude (oui tout à fait pour l’instant le felden c’est un nom de marque c’est pas le générique. Ok d’accord)

Mais ça calme hein ça calme

(Oui oui mais le médecin a raison vous pouvez pas en prendre trop)

Et parfois je fais pendant les douleurs peut-être c’est pas bon je fais je prends deux deux (deux d’un coup) deux d’un coup le felden et après je le prends pendant 4 jours (ah d’accord) je sais pas si c’est pas bon moi j’ai pas posé la question parce que il m’engueule il m’engueule tout le temps (il vous engueule ?) Bon m’engueule il dit que.. (il vaudrait mieux éviter en tout cas quand vous en prenez deux d’un coup c’est pas effectivement c’est pas très bien mais alors il faut vraiment bien boire le journée) mais ça calme après quelques heures ça calme (ça calme plus que le coDafalgan ?)

Mais le coDafalgan je l’ai pris quand j’avais pas de felden (ah d’accord) j’avais là j’ai donné un coup de fil et j’ai demandé si je pouvais le prendre et il m’a dit oui (ok mais ça fait le même effet que le felden quand vous l’avez pris une fois là ?)

Je crois que ça fait moins d’effet le codalfagan c’est un médicament générique comme vous dites ? (Non c’est pas un médicament générique c’est un médicament de marque mais il y a pas de générique donc l’assurance peut rien vous dire elle est obligée de rembourser le felden aussi il y a pas de génériques)

Parce que la caisse maladie elle fait des histoires avec les génériques ? (Non elle fait pas des histoires elle demande que vous preniez le générique pour qu’elle vous rembourse mais vous avez le droit de dire non mais à ce moment vous devez payer plus 20 % le médicament)

Si on prend pas le générique ? (Oui)

Pour moi je vous dit ça suffit qu’il dise le médecin et pour moi c’est bon un générique ou normal c’est bon mais il doit dire le médecin c’est lui qui décide les médicaments c’est pas le patient (d’accord et pas le pharmacien non plus)

Pas le pharmacien non plus

(D’accord c’est clair)

**Patient 5**

(Vous voyez ce que c‘est un médicament générique ?)

Moi j’ai vu seulement j’ai changé le Brufen vers l’Irfen parce que j’ai vu le médecin il m’a dit que c’est à peu près c’est la même chose même le pharmacien il m’a dit aussi que c’était comme ça et je le prends et pour moi ça va ça a pas changé de choses et ça aide aussi

(Quand vous dites c’est la même chose qu’est-ce que vous entendez par là ?)

Pour moi je vois pour ma douleur ma inflammation que j’ai à mon dos par exemple aussi mon inflammation que j’étais très crispé je voyais que ça me soulage

(ça vous soulage l’Irfen comme le …)

le Lyrica par exemple quand je l’ai pris il m’a changé beaucoup aussi parce que avant j’arrivais pas à gérer à 100% ma douleur et là avec le Lyrica ça a changé je sens que la douleur c’est moins mais après un certain temps je sens que la douleur ça va monter d’un coup parce que je vois comme je vous ai dit j’ai fait aussi l’attaque cérébrale avant c’était ma femme qui m’a fait comprendre les choses parce que mon côté mon gauche pour moi c’était presque pareil j’arrivais pas à serrer les choses avec ma main maintenant j’arrive à serrer un petit peu plus mais si c’est un truc très lourd il tombe et même si je suis avec un verre d’eau si je marche d’ici là-bas par exemple d’ici là-bas des fois il m’arrive que pour moi la main c’est serré mais il arrive que le verre il glisse par terre.

(D’accord. Alors le Lyrica c’est aussi un générique ?)

……ça je sais pas si c’est un générique c’est mon médecin qui m’a proposé ça il m’a dit parce que je prenais du mydocalm je le prenais 4 ou 5 par jour pour relaxer ma musculation il m’a dit je vous propose ça pour laisser ça parce que ça fait beaucoup de médicaments il m’a mis le Lyrica et ça m’a aidé un peu

(Et alors cet Irfen ça vous a aidé aussi ?)

Oui moi je trouve que le Brufen et l’Irfen c’était pareil

(Donc ça veut dire que l’Irfen et le Brufen ils contiennent la même chose ?)

Pour moi oui parce que je voyais ça faisait aussi ça soulageait aussi mon inflammation

(Votre inflammation était soulagée aussi. Ca changeait quelque chose au niveau des effets secondaires ?)

…..avec le Lyrica par exemple, Irfen même Brufen j’ai pas fait attention, je travaillais avec les échafaudages tout ça il faut faire très attention aussi même quand je prenais pas de médicaments c’est normal on travaille à 15 mètres 20 mètres des fois mais maintenant que je prenais des médicaments le mydocalm et tout ça je voyais que quand j’étais en haut de l’échafaudage et que je voyais que il bougeait un peu par exemple l’échafaudage là je peine à sentir moi même si vous voulez je prenais la main j’étais pas sûr pour marcher. J’ai pas trouvé de changement avec le Brufen ou l’Irfen mais avec le Lyrica oui beaucoup je voyais que à rester concentrer sur certaines j’arrive pas et même pour conduire la voiture et tout ça j’étais pas sûr de moi même sur la route, après quelques temps je prenais ma voiture je faisais très attention sur la route même avec mon beau-frère à côté et tout ça parce que lui-même il faisait très attention à moi parce que moi-même j’avais la trouille parce que comme je le disais avant ma famille c’est tout et on est là tous les trois parce que j’ai un fils de 18 ans pour moi on est tout et je suis très heureux de ce qu’on a fait moi et ma femme parce que c’est notre fils on est bien et je fais très attention à ma vie et à la vie des autres aussi parce que c’est un comment on dit en français respect envers les autres surtout, si je vois quelques personnes en difficultés je vais donner un coup de main aussi je respecte beaucoup les autres, j’aime bien du respect envers moi c’est pour ça je donne du respect

(Alors en somme pour vous ce qui fait le plus de différence c’est pas tellement si c’est le médicament original le Brufen ou le générique l’Irfen c’est plus de manière générale l’effet que vous font les médicaments ?)

oui l’effet que je me sens par exemple avec le Tryptisol, comme je disais l’Irfen et tout ça c’est la même chose pour moi il m’a proposé mon médecin et moi j’ai confiance en mon médecin et au pharmacien aussi comme je crois que quelqu’un il a confiance quand il me demande de faire une maison et moi je crois et vous avez étudié pour faire ça vous savez plus que nous comment il faut faire pour notre santé aussi et comme je le disais avec Brufen et Irfen j’ai pas vu de différence seulement que j‘ai je crois que c’était avec le Brufen je me rappelle pas j’avais seulement l’estomac qui gonflait beaucoup ça oui

(Et avec l’Irfen aussi ?)

ça oui

(Qu’est-ce qu il vous a dit votre docteur quand il vous a proposé de prendre plutôt l’Irfen ?)

Bah il m’a dit que ils avaient des médicaments génériques il a vu dedans la liste il a dit que les assurances et tout ça ils préféraient aussi parce que c’était moins cher moi j’ai demandé est ce que ça change quelque chose il a dit que non c’est la même chose seulement que il y a ça change le nom seulement ça et j’ai continué à le prendre

(Vous avez pas remarqué de différence, c’est pas plus difficile à avaler ?)

non par moments moi je me rappelle pas j’ai pas fait grand attention de voir changement (vous avez pas vu grand chose) non mais comme je le disais avec le Tryptisol là je le prends parce que mon médecin il m’a dit que après un certain temps un mois deux mois on voit le changement de la douleur tout ça mais je prends parce que j’ai confiance avec médecin mais je me sens la tête vide, la bouche sèche et tout ça et des fois j’ai l’estomac que ça gonfle beaucoup et ça il……… je sais pas comment dire quand je sens ma tête vide c’est pas moi que certains moments je suis en train de parler avec ma femme et ma femme me dit tu dis quoi ? Parce que je me trouve avec ma tête ailleurs qui est vide pour moi je me sens vide certains moments et……………. je le prends parce que ça va me soulager et j’ai beaucoup de confiance comme je l’ai dit au médecin et tout et je le prends et si je vois que d’ici à quatre mois ça va pas moi je dis aussi à mon médecin qu’on arrête

(C’est celui que vous voulez le plus arrêter le Tryptisol ?)

Oui parce que là je me sens, avec le Lyrica j’avais des trucs que je voyais à l’image quelque chose qui dure un instant c’est pas mais au début mais après mon corps je crois s’est habitué et je voyais que j’arrivais à faire des choses à conduire à faire des petits choses à être concentré ce que j’arrive pas maintenant avec le Tryptisol parce que je si je dois me concentrer sur certaines choses mais j’arrive pas à les suivre jusqu’au bout

(Vous pensez que c’est lié à quoi dans ce médicament ? vous pensez que c’est parce que c’est un générique ?)

…………..ça je sais pas

(Vous avez regardé la petite notice dedans, le petit papier avec les explications ?)

moi je l’ai regardé une fois et j’ai vu que il y avait ……….beaucoup de contre indications là et le médecin il m’a donné quelque chose quand j’ai commencé ma tension et que ça s’élevait un peu j’ai vu que ma pulsation cardiaque ça augmentait un petit peu les pulsations ça a duré 1 semaine 15 jours je crois et après ça commence à être meilleur de nouveau et là je vois que je suis bien avec ma tension et tout comme je vous l’ai dit mon problème je vois c’est la bouche très sèche

(Et la tête vide mais vous avez vu ça dans la notice aussi ils en parlent ?)

J’ai j’ai lu une fois seulement j’ai pas autant remarqué si il y avait ça ou pas j’ai dit à ma femme regarde toi si il y a quelque chose parce qu’elle comprends beaucoup plus le français que moi j’arrive pas à le comprendre et j’ai dit écoute moi je suis là et tu le sais ma femme le sait que je moi préfère sortir d’ici de ma maladie et faire quelque chose un petit boulot n’importe lequel l’important que j’arrive à le faire à le soutenir jusqu’à la fin

Avez-vous été chercher des informations à propos des génériques sur internet ?

(Est-ce que quand on vous donne un médicament vous allez chercher de l’information ?)

Oui

(Vous allez où la chercher ?)

C’est pas moi c’est ma femme c’est elle qui va s’informer de tout ça pour voir le médicament par exemple quand il m’a donné le Tryptisol on a regardé c’était un anti dépresseur et là moi j’ai dit je suis pas fou que je vais prendre un antidépresseur et après ma femme on a regardé ils donnent ça même pour la douleur et je le prends mais jusqu’à maintenant je vois pas de soulagement pour ma douleur je vois seulement des choses négatives

(Et alors votre femme elle va où regarder ?)

Sur internet elle recherche sur internet (sur internet) comment je l’ai dit j’ai beaucoup de confiance pour mon médecin parce que je sais qu’il a étudié pour être médecin et il est sûr de ce qu’il fait

(Bien sûr mais vous vous aimez bien en savoir un peu plus ?)

Bien sûr parce que c’est ma santé

(Vous avez tout à fait raison donc vous allez ou votre femme vous lui avez dit je prends l’Irfen le Tryptisol tout ça et elle va sur internet et elle regarde un peu ce qu’on dit elle a pu vous rassurer avec le tryptisol et vous dire oui c’est un antidépresseur mais on le donne aussi exprès pour la douleur et puis elle est allée voir l’Irfen et tout ce qui est pour les génériques ?)

Oui comme je l’ai dit moi je prends certains médicaments et aussi l’attaque cérébrale je veux pas dire que c’est seulement les médicaments j’arrive pas à rester concentré par exemple si je veux lire un truc il faut que je le relise 4 ou 5 fois parce que ça me fatigue et c’est pas facile à gérer et sur internet tout ça par exemple j’ai facebook j’arrive pas à rester sur l’ordinateur plus que 15 ou 20 minutes parce que après j’ai mal à la tête

(Donc vous allez chercher des informations ou votre femme et est-ce qu’elle cherche ailleurs que sur internet ?)

Non je pense c’est internet

(Et puis elle cherche quoi est-ce qu’il y a quelque chose de spécial ?)

C’est par exemple pour certains médicaments il y a des contre indications que moi aussi je dois regarder mon cœur et on va s’informer et regarder si il y a des contre indications pour mon cœur ma tachycardie que j’ai et j’ai beaucoup de confiance avec mon médecin et aussi on regarde bien pour être encore plus sûrs

(Et puis est-ce que pour les génériques c'est encore plus une question d’être sûr ou bien c’est comme pour tous les autres ?)

C’est comme pour tous les autres

Qu’est-ce qui vous feriez hésiter à prendre un générique ?

(Vous n’avez pas de souci spécial par rapport aux génériques?)

non non

(Si on vous donnait un générique comme médicament pour le cœur ?)

Non

(Non plus vous avez pas de souci)

Non c’est mon médecin si il est d’accord parce que moi je le crois

(Si votre médecin vous dit c’est la même chose)

C’est la même chose il y a pas de contre indication et après comme j’ai dit ma femme va contrôler aussi moi je le prends même que ma femme elle va pas contrôler moi je le prends parce que j’ai confiance à le médecin

(Donc ça veut dire que pour vous que ce soit l’original ou le générique la question est toujours la même est-ce que c’est bon pour moi est-ce qu’il y a pas de contre indications ?)

Oui oui bien sûr parce que je le prends parce que c’est mon médecin qui me l’a donné il sait plus que moi surement ce que j’ai besoin et comme j’ai eu l’attaque cérébrale et de là je vois j’ai eu de bonnes choses jusqu’à maintenant avec les médecins parce que avant j’étais quelqu’un qui était pas avec les médicament et tout j’évitais tout…..jusqu’au moment que je suis arrivé à rester bloqué j’ai pris un médicament et j’ai commencé à travailler comme je le faisais avant j’ai fait deux ans trois ans avec les médicaments j’arrivais à bouger après je suis resté bloqué tout ça ça fait 2008 je suis en arrêt de travail comme je l’ai dit et la même d’ici le mois d’avril ça s’arrête ma perte de gain et c’est pas facile à gérer les choses j’ai beaucoup de confiance avec les médicaments

Votre pharmacien vous a-t-il déjà proposé de substituer le médicament original pour un générique ?

(Et toujours avec ces médicaments est-ce que c’est arrivé que le pharmacien vous propose des génériques ?)

mon pharmacien il m’a proposé des génériques au tout début je crois c’était l’année passée tout ça ou quelques années en avant il m’a proposé l’Irfen il m’a dit c’est la même chose j’ai dit oui parce que des fois il avait des médicaments je me rappelle plus c’est pour la tachycardie euh le Beloc zok il m’a proposé un générique moi j'ai dit qu’est ce qu’ il a pris mon médecin c’est le Beloc zok moi je prends le Beloc zok après je vais demander à mon médecin si on peut le changer il m’a dit on laisse comme ça parce que on avait bien réussi à avoir mon cœur à avoir la même pulsation parce que avant c’était très haut aussi il m’a dit de rester comme ça

(D’accord donc c’est plutôt votre médecin qui choisit le générique et pas le pharmacien ?)

non le pharmacien il m’a dit une fois (il vous a proposé) oui moi j’ai dit écoutez je vois dans la comment on dit en français euh la recette je vois mon médecin il a écrit ça vous me donnez ça les génériques je sais pas ça je connais pas les médicaments même que vous me dites que c’est bien moi le médecin il m’a dit ça moi je prends ça et puis c’est tout ……si mon médecin il change le médicament et il dit que c’est la même chose de l’Atacand par exemple que je prends pour la tension si il dit que c’est la même chose je prends aussi, j’avais avant la pour le cholestérol très haut avant j’avais un médicament je me rappelle pas le nom il m’a changé avec un autre c’est vigi un truc comme ça je me rappelle plus le nom et il me l’a changé je prends ça et je vois que ça s’améliore je continue à le prendre

(C’est un générique ou c’est un autre médicament ?)

Je sais pas si c’est un générique si c’est générique c’est un autre médicament avant je prenais autre chose je me rappelle pas ah c’était euh ……..c’est simvastatine je crois pour le cholestérol et maintenant c’est vigi c’est une grosse boite je sais pas comment ça s’appelle désolé

(Donc là aussi pour le cholestérol c’est plus le médicament que le médecin vous donne que de savoir si c’est le générique ou pas ?)

je pose pas la question qu’est ce qu’il me dit mon médecin je prends parce que comme j’ai dit j’ai confiance et…………si ils disent que c’est un générique je ne sais pas parce que le Brufen qu’on a changé avec l’Irfen c’est mon pharmacien qui m’avait dit avant et le même médecin il m’a dit aussi après et je prends mais j’ai jamais demandé au pharmacien ou au médecin si c’est un générique parce que j’ai confiance ce qu’il me donne parce que il me donne je crois un médicament pour ma santé pour chercher à me guérir il va pas me donner quelque chose de mauvais

(à votre avis les génériques qui les fabriquent ?)

ça je sais pas je crois ……………qu’il y a des responsables des médecins et des comment on dit ça…..étudiants qui font des études pour les médicaments et qui cherchent d’autres choses qui ça va donner la même chose par exemple le Brufen et avec y a quelqu'un de responsable un médecin quelqu’un qui connaît plus les deux médicaments il voit les effets tout ça il pèse si ça fait la même chose ou pas je crois ils mettent pas des médicaments qui vont empirer la santé

(Donc ce n’est pas la même personne ?)

Je sais pas j’ai jamais posé la question j’ai jamais recherché c’est qui fait les médicaments tout ça je posais pas la question c’est qui qui fait les médicaments

(Donc ces génériques c’est une sorte de copie ?)

Moi Oui je crois il y a des copies du Brufen le Lyrica je sais pas je crois il y les mêmes choses, les mêmes doses dedans seulement il y a des autres façons de le faire des autres plantes je sais pas quoi qui ça coûte moins que les autres et on peut avoir le même médicament à moitié prix je crois ou que c’est moins cher et c’est la même qualité qui font la même chose que les autres

Moi comme je l’ai dit je suis pas quelqu’un qui est dedans qui a étudié tout ça mais je crois que c’est ça mais je vois on a changé le Brufen et c’est….

(sans changement) oui

**Patient 6**

(à votre avis qu’est ce que c’est un générique ?)

Alors un générique c’est une trouvaille qui a été faite avant les nouveaux médicaments c’est plus ancien et j’ai fait l’essai avec le Tramal j’ai le médecin m’a prescrit du Tramal et je suis arrivé à la pharmacie qui m’a dit on a plus pour le moment mais en revanche j’ai un générique c’est parfaitement la même chose et j’ai essayé ce générique et effectivement j’ai pas eu d’autres effets ni secondaires ni meilleurs ni moins bons

(Pour vous ça a fait exactement le même effet ?)

Même effet

(D’accord c’était sous forme de comprimés ?)

Non pas de comprimés, de liquide

(D’accord du Tramal en liquide ; donc pour vous un générique ça contient la même substance exactement ?)

à peu près oui

(Quand vous dites à peu près c’est)

à peu près c’est que comme il y a deux noms on croit qu’il y a une différence autrement ça ne se justifie pas

(D’accord et vous pensez que tous les génériques sont aussi efficaces que l’original ?)

Alors ça difficile de le dire puisque je n’ai pas essayé mais ….à en croire l’entourage oui c’est peut-être pareil

(D’accord donc vous avez pas de connaissance qui vous aurait dit oh bah moi quand on m’a proposé le générique c’était moins bon ?)

Non jamais entendu ça

(D’accord et puis du point de vu des effets indésirables pour vous c’est ?)

Non je n’ai pas eu d’effets secondaires

(D’accord et vous pensez que les génériques sont mis sur le marché et qu'ils ont le même contrôle ?)

Je pense que il y a eu une évolution dans la fabrication de……ces médicaments et que maintenant on peut en fabriquer beaucoup plus avec des machines qui vont beaucoup plus vite mais que le liquide il y a en a moins que dans les autres les anciens qui étaient plus chers

(Ah d’accord mais quand vous prenez du Tramal qui est donc l’original il y a plus de liquide que dans les génériques ?)

Non c’est 50 ml

(D’accord donc c’était bien avant qu'il y avait plus ?)

Oui

(Vous avez l’impression que même l’original ils ont diminué les quantités ?)

Ils ont diminué oui

(Et puis le générique aussi ils ont diminué ?)

Voilà

(Et puis vous pensez c’est parce que il y a les génériques qui sont venus sur le marché ?)

Probablement oui…….et puis euh on vit une époque ou c’est la mode on change les étiquettes et on augmente les prix et le coût de la vie par voie de conséquence

(Ouais c’est vrai d’accord. Quand vous avez eu des génériques vous avez eu l’impression que vous aviez plus de peine à les avaler ?)

Non absolument pas c’est un liquide qui a le même goût même

(Qui a le même goût ?)

Le même goût

(La même couleur aussi ?)

euh je ne saurai répondre parce que c’est dans un flacon de couleur brun foncé et on voit pas la couleur même si on y verse dans une cuillère pour l’avaler euh c’est blanc tout les deux

(Tous les deux sont blanc d’accord et tous les deux ont le même goût ?)

Tous les deux ont le même goût

(D’accord et il y en a pas un qui est plus pâteux que l’autre ?)

Non absolument pas en ce qui concerne donc le Tramal

(D’accord ok vous avez regardé si c’est la même firme pharmaceutique qui fabrique le original et le générique ?)

Non j’ai pas regardé qui fabriquait

(Vous avez regardé les notices si elles sont la même chose ?)

Non plus je les ai pas consultées parce que c’était écrit tellement petit qu'il me faudrait deux paires de lunettes pour pouvoir déchiffrer ce qui est inscrit

(Ok d’accord est-ce que vous vous hésiteriez à prendre un générique ?)

Non

(Non donc pour des problèmes cardiaques ou pour d’autres maladies ?)

Non c’est le spécialiste le pharmacien ou le médecin dit c’est la même chose je le crois

(D’accord ok comment vous avez entendu parlé des génériques ?)

Oh bien euh…par ………..Euh les les ………des amitiés prennent la même chose par exemple le chez les sportifs ils prennent tous le même produit donc euh…….c’est par mon entourage et par le pharmacien et par le médecin aussi

(Le médecin quand il vous a prescrit le Tramal en générique il vous a expliqué un peu ce que c’était ?)

Non pas le médecin c’est le pharmacien

(Et qu’est-ce qu'il vous a dit vous vous souvenez ?)

Que c’était pareil

(C’était pareil d’accord mais il a pas donné plus d’explications ?)

non non non

(D’accord est-ce que vous avez été cherché des informations sur les génériques ?)

Non

(Ok donc le médecin vous en a prescrit sans vous donner tant d’explications le pharmacien vous a juste dit que c’était la même chose) absolument (et que finalement ces informations là vous ont suffi pour faire confiance) absolument pour faire confiance au spécialiste

(D’accord pour les autres médicaments que vous prenez est-ce qu’on vous a proposé des génériques ?)

Non par exemple le Rivortil je sais pas si il y a un générique

(Je sais pas non plus euh qu’est-ce que vous prenez d’autre)

L’Allopur alors il y a un générique

(Oui le Zylorik on a vu, le Ventolin il y a un générique aussi)

Alors la Ventolin euh j’ai toujours pris la Ventolin on m’a jamais proposé autre chose

(D’accord si on vous proposait autre chose vous accepteriez ?)

……Bah j’essaierais je verrais bien si….C’est donc un médicament contre l’asthme pour favoriser la respiration j’essaierais mais je garderais en réserve quand même le (Ventolin) le Ventolin

(D’accord donc vous faites quand même un tout petit peu moins confiance pour un générique pour l’asthme par exemple)

Oui c’est à dire qu'il y en a eu beaucoup de médicaments pour l’asthme on s’en occupe seulement maintenant parce que il y a une grande pollution mais moi qui suis né en 23 avec de la côte de lait puis qui s’est transformé en asthme et bien il y a eu un tas de médicaments surtout qui sont venus d’Angleterre sous forme d’un liquide qu'on mettait dans un appareil vous injectez deux trois jusqu’à 4 euh…pulsions

(D’accord mais si j’entends bien vous me direz si j'ai bien entendu pour le Tramal vous avez pas hésité) non j’ai pas hésité (pour l’asthme vous êtes un tout petit peu plus précautionneux)

Absolument j’essaie et si je constate que c’est pareil je n’hésiterai pas à prendre le meilleur marché

(Mais est-ce que vous êtes d’accord est-ce qu'on pourrait dire que pour le Tramal vous avez fait confiance au médecin et au pharmacien mais pour l’asthme vous allez plutôt faire confiance) à moi même

(Ok d’accord et puis par exemple le Dormicum si il existait un générique vous seriez d’accord ?)

Oh oui parce que c’est pas la même chose pour dormir ou pour pas dormir euh bon bah on verra bien

(Ok d’accord donc il y a quand même certains symptômes certaines maladies ou votre expérience est plus importante ?)

oui il ya …….euh de la cortisone dedans que il y a on prend d’abord le Ventolin et puis après la comment ça s’appelle ça existe en disque( Seretide ?)

Seretide alors je sais pas si ça existe (je suis pas 100%sûre mais je crois que ça existe)

Alors pourvu qu’il y ait de la cortisone dedans je prendrai sans autres

(D’accord ok)

Parce que c’est la base

(Oui tout à fait)

Et ça a été découvert en 1933 j’ai lu ça dans le dictionnaire

(Oui tout à fait ça remonte à loin maintenant, d’accord je crois qu’on a répondu à toutes les questions que vous avez eu une expérience, que vous faites plutôt confiance sauf pour l’asthme)

Pour l’asthme je fais une réserve parce que j’ai pas essayé

(Oui tout à fait d’accord ok je vous remercie beaucoup)

**Patient 7**

(A votre avis qu’est ce que c’est un générique ?)

C’est l’imitation d’un produit, on utilise tout le savoir mis en place par d’autres pour le copier. (c’est donc une copie ?) oui

(A votre avis la copie contient la même substance ?)

A mon avis oui sinon on ne pourrait pas l’interchanger

(A votre avis la copie a la même efficacité ?)

Je ne peux pas me prononcer, j’en ai jamais pris, mais à mon avis oui

(A votre avis la copie a plus d effets indésirables ?)

Non c’est les mêmes effets

(A votre avis les génériques sont mis sur le marché et qu'ils ont le même contrôle ?)

Je ne sais pas vraiment, très certainement, ce n’est pas le genre de choses qu’ils laisseraient passer

(A votre avis la copie est plus difficile à avaler ?)

Je n’ai pas d’expérience mais je pense qu’ils essaient de reproduire jusqu’à la représentation du médicament pour ne pas troubler les patients

(A votre avis qui les fabrique ?)

J’ai aucune idée

(Hésiteriez-vous à prendre un générique ?)

Non pas du tout non, du reste je me demande pourquoi on me n’en a jamais proposé

(En avez-vous entendu parler ?)

Oui, les médicaments arc-en-ciel (sur les panneaux ?) oui sur les panneaux publicitaires et à la TV on est bien obligé d’entendre même si on ne veut pas

( Internet ?)

Non jamais (pour un autre médicament ?) non pas pour un médicament, mais pour une maladie que je ne connais pas et qui m’intrigue (comment faites-vous alors ?) je tape directement le nom (sur quel moteur ?) google (quels sites avez-vous lu?) je lis surtout wikipedia (pourquoi ?) c’est le site qui donne le plus d’informations précises

(Votre médecin vous en a-t-il prescrit ?)

jamais, mais je pense qu’il n’y a pas de génériques pour les médicaments que je prends (MST, Rivotril, Surmontil), sur les antibiotiques que j’ai pris là oui il y a des génériques mais il ne m’a pas proposé et j’ai pas demandé

(et votre pharmacien ?)

non plus il m’a jamais proposé et je pense qu’il ne serait pas tellement d’accord que je demande car les produit est moins cher et sa marge serait plus basse

(Etes-vous d’accord que l’on vous prescrive un générique ?)

oui, tout à fait, pour les antibiotiques qui sont tellement chers je vais demander la prochaine fois

**Patient 8**

(A votre avis, qu’est-ce qu’un générique ?)

C’est un médicament qui… est… un nouveau médicament, non pas un nouveau médicament, un ancien médicament qui… après de nombreuses années, est dans le domaine … commun, et donc c’est un médicament qui a les mêmes molécules, les mêmes propriétés qu’un ancien, mais moins cher parce qu’il n’a pas eu la recherche pour le faire… Il est moins cher parce qu’il a simplement repris les molécules telles qu’elles ont été dites et fabriquées une première fois par le premier médicament qui est non-générique… et le non-générique étant plus cher vu qu’il y a eu tout le travail de la recherche pour le faire… et après plusieurs années, voilà il y a un générique. Mais c’est le même.

(Le même?) ça veut dire qu’il a peut-être pas forcément la même couleur, la même forme ou j’en sais rien, mais que… à ce qu’on m’a dit… à l’intérieur la formule est la même.

(même substance ?) oui tout à fait.

(‘à ce qu’on m’a dit’?) le doute que j’ai… c’est mon papa… qui au début des génériques m’avait dit que ce n’est pas aussi simple que ça… qu’en fait il y avait toujours quelque chose qui n’était pas dit dans le médicament de base… par exemple dans quel ordre ils mettent la composition ou quelque chose dans le genre… et que, finalement, le générique n’était pas exactement le même parce que il y a avait la même composition, la même formule mais ça n’avait pas été dit dans quel… de quelle manière les mettre ensemble ou les composer. Ça c’est mon papa qui me l’avait dit. Mon papa m’a dit ça moi j’ai entendu que le générique était identique et… en fait, je me suis dit que si les médecins me disent que c’est la même chose, mon papa n’étant pas médecin, j’allais plutôt me fier au médecin.

(conclusion ?) ma conclusion, c’est que certainement c’est la même chose, mais il y a quand même ce petit point d’interrogation qui veut dire que mon papa a soulevé quelque chose… qui veut dire que… parce que il paraîtrait que certaines personnes quand on leur donne le générique, elles disent que ça leur fait pas le même effet que quand elles prennent le médicament de base… il paraîtrait que pour certaines personnes ça ne convient pas, alors je ne sais pas si c’est dans la tête ou d’où ça vient… ou si c’est cet ordre-là de formulation qui fait que c’est différent, moi en tous cas j’ai pas du passer d’un… j’ai des génériques mais je n’ai pas passé d’un original à un générique donc je ne peux pas dire… si ça fait une différence, mais selon moi c’est la même chose. Mais, comme vous l’avez senti, il y a quand même en moi cette idée de me dire ben si certaines personnes sentent une différence et ne supportent pas le générique et ils disent que c’est l’original qui est vraiment mieux, est-ce que mon papa avait peut-être une petite raison (rit) de dire ce qu’il a dit, mais… voilà, moi-même en fait je crois aux génériques… je pars sans a priori même si j’ai ça dans la tête.

(‘j’ai ça dans la tête’, change qqch?) euh… pour moi non, parce que justement j’ai pas passé d’un à l’autre …. Est-ce que ça aurait changé si j’avais eu un original et qu’on m’avait dit ben on va changer maintenant pour un générique… je sais pas si ça ça aurait participé inconsciemment au fait que peut-être j’aurais dit oup ça ça fait pas le même effet… je ne sais pas, mais moi je n’ai pas eu ce cas-là, et sans avoir eu ce cas-là, en ayant un générique, je trouve qu’il va très bien, donc c’est vrai que je dirais aux gens.. ben, dans ma tête, pour moi , le générique est très bon.

(quels génériques ?) alors j’ai par exemple l’Irfen, j’ai mepha… euh.. j’ai… je retiens plus… j’en ai d’autres… déjà un antidépresseur… je retiens jamais son nom… et j’ai pas connu la… la formule de départ.

(recette de chef?) c’est un peu ça, ce qu’il m’a expliqué c’est un peu ça. En fait il y a noté les ingrédients de la recette, mais pas dans quel ordre et de quelle manière ou par où commencer… c’est ça !

(efficacité ?) oui, c’est le même, la même efficacité

(effets secondaires ?) je suppose ça devrait faire les même effets secondaires…

(copie ?)… copie… pas vraiment parce que… dans copie, pour moi, c’est un peu négatif, … c’est comme les tee-shirts, il y a des copies, on le fait plus ou moins pareil, donc une copie, ce serait justement de prendre la même forme, la même couleur, pour qu’ils soient pareils, et puis ça veut pas dire qu’il y a exactement… enfin une copie… il faut qu’il ressemble, mais ça veut… Ouais, pour moi c’est pas une copie. Le mot copie pour moi n’est pas approprié parce que pour moi c’est plus qu’une copie parce que c’est vraiment… c’est le même… mais… pas de la même maison… donc c’est pas réellement une copie.

(MD a parlé de génériques et prescrit ?) oui… ( ?) Il m’a dit que je n’avais aucun souci à me faire, qu’il y avait les mêmes contrôles… que c’était un médicament comme un autre, enfin je veux dire que c’était… ils avaient les mêmes surveillances et que c’était pas comme ces médicaments qu’on fait venir de Chine ou n’importe quoi… que c’était des médicaments qui restaient… qui venaient des laboratoires qui sont reconnus et qu’il n’y avait aucun souci à se faire, que c’était à moi de dire si ça faisait de l’effet, mais ce serait la même chose pour un autre médicament… et que le générique avait le grand plus, c’est que il est moins cher et que par rapport aux coûts de la santé, c’était bête de pas les utiliser s’ils existaient parce il y avait assez de médicaments où il y avait pas de générique, donc quand il y avait cette possibilité-là, il fallait en profiter.

(Qu’est-ce qui vous ferait hésiter?) … euh… à part si quelqu’un me disait ‘écoute j’ai pris ce générique, et franchement ça m’a rien fait ou j’ai eu des boutons…’ là si quelqu’un m’en parlait, là peut-être ça me ferait douter, mais sur quelque chose que je ne connais pas, sur ceux que je connais, on pourra pas maintenant me dire que c’est pas bien.

(pharmacien propose substitution?) il paraît que normalement il devrait le faire et que…. Le mien non. D’ailleurs une fois le médecin avait marqué le nom d’un générique et puis il l’avait pas et puis il m’a dit on va vous donner l’original et moi j’ai dit ‘écoutez c’est pas… c’est pour continuer, j’aimerais à tout prix continuer avec ce générique, j’en ai pas besoin dans la minute qui suit, vous pouvez le commander’, en fait eux ils étaient même prêts à me donner l’original.

(si pharmacien propose ?) … euh… j’accepterais, peut-être que je demanderais juste si mon médecin est d’accord par rapport aux… je devrais pas avoir plus confiance dans mon médecin que… dans le pharmacien qui sait son métier mais… c’est vrai que par rapport au reste de mes médicaments, qu’il sache… voilà quel générique il met… pour les interactions, pour… enfin tout ! j’aurais bien aimé avoir juste le feu vert, je serais d’accord mais je dirais est-ce qu’on peut juste appeler le médecin pour qu’il dise si lui il est d’accord. ( ?) c’est vrai que je serai contente que la pharmacie me dise qu’il en existe, que si mon médecin l’a loupé, qu’ils me le disent, mais c’est vrai que j’aimerais bien que mon médecin, juste donne le feu vert.

(comment vous renseignez-vous ?) … par rapport aux médicaments que je prends ou par rapport aux médicaments en général ? ceux que je prends, il y a les notices. Je saute la partie des effets secondaires parce que j’ai compris qu’ils ont tous les mêmes effets secondaires, donc ça servait à rien de me rendre malade en disant je risque ça et ça… Je lis, j’essaie de voir ce qu’ils disent dans la composition, des fois ils disent que c’est pas bien de prendre avec un antidépresseur… c’est vrai que j’ai déjà demandé à mon médecin, ils disent de pas prendre avec un antidépresseur, est-ce que… et pi là il me rassure en me disant que c’est… lui il prend sur lui, c’est lui qui a décidé, il y aura aucun souci d’interactions, en fait j’ai confiance au médecin, donc je ne suis pas médecin, je lis, je regarde des fois aussi sur internet un peu, c’est comme ça d’ailleurs que j’avais vu certains nouveaux médicaments qui existaient, certaines choses… je regarde un peu ce qui existe, mais après c’est mon médecin… je viens avec ma photocopie d’un médicament, je viens avec ma notice, mais je viens vers mon médecin. J’ai toute confiance en lui, s’il me dit c’est bon, c’est ok, j’y vais.

(internet ?) je regarde un peu ce qui existe, ce qui se fait… c’est là que j’ai regardé un peu les médicaments, notamment les antidépresseurs… que j’avais vu que j’ai fait un petit peu le tour de ce qui existe… enfin qu’il existe pas beaucoup d’autres choses, parce que je comprenais pas que mon médecin me trouve pas plusieurs antidépresseurs pour remplacer ceux que j’avais pris et c’est là que j’ai vu un peu… qu’on a fait le tour. Je l’utilise… mais un peu comme mme tout-le-monde, c’est-à-dire que j’ai pas un programme de médecin, je suis une simple… utilisatrice ( ?) Google pi j emets soit ma question soit les mots-clés puis je pars comme ça pi après en fait d’un mot je pars sur autre chose, je fais des chaînes… et j’arrive pas… en fait finalement j’arriverais pas à les refaire certainement parce que c’est tout d’un coup quelque chose que me fait tilt et je pars… et puis tout à coup j’ai ma réponse ou j’ai mon médicament et… j’ai tout à coup quelqu’un qui dit quelque chose sur ma maladie… c’est aussi par rapport à ça… je l’utilise mais je suis un peu déçue… parce que… c’est sûr c’est une machine, c’est très impersonnel… et je trouve que des fois pour nous ça complique les choses… c’est comme par rapport aux molécules, quand on cherche à savoir quelque chose… il y a trop de mots qui arrivent dans ma tête et au lieu de m’aider ça a tendance à me desservir… je retiens plus les mots, je retiens plus rien, j’imprime certaines choses pour les lire mais finalement après je sais même plus où mettre ces papiers…ça m’a servi pour certaines choses mais pour d’autres ça me rend plus nerveuse, plus stressée, plus… c’est difficile de trier… il faudrait avoir un programme pour les malades, ou je sais pas… pour que ce soit en même temps facile d’accès et en même temps pas tout bête, idiot… pour quelqu’un qui comprend sa maladie ou qui comprend les choses, qu’il puisse avancer quand même, que ce soit pas trop banal… mais pas trop pointu comme pour quelqu’un qui a fait médecine, qui sait ce qui se cache derrière certains mots, parce que nous on sait pas forcément…

(internet pour génériques ?) au tout début, j’ai vu… parce que je comprenais pas pourquoi j’avais mepha alors qu’il existait d’autres génériques et puis finalement j’ai compris… je suis allée j’ai vu qu’il y en avait d’autres sur internet… j’ai vu que c’était la même chose pi j’ai demandé à mon médecin pourquoi mepha pi mon médecin m’a dit ‘parce que j’ai l’habitude de prescrire mepha, et que ça s’est bien passé, mais si vous préférez autre chose, je vous prescrit un autre générique’, j’ai dit bon ben non, mais y a-t-il une raison particulière, et c’est parce qu’il avait l’habitude… et je sais pas pourquoi il avait cette habitude-là… si les gens de mepha sont passés chez lui… lui faire comprendre qu’ils étaient un générique très bien ou… suite à ça il prescrit mepha, j’en sais rien… c’est vrai que je m’étais posé la question, parce que je comprenais pas pourquoi ce générique-là et pas un autre et il m’a expliqué… quand on avait l’habitude d’un certain générique, on avait l’habitude normalement de continuer dans le même… de pas changer chaque fois de générique… il voyait pas le… à quoi ça allait aider, ils étaient tous dans la même gamme de prix, donc voilà !

(explications à autres patients ?) …. Que certaines fabriques qui font les génériques… font les mêmes médicaments… mais moins chers surtout à cause de la recherche… parler en fait qu’un médicament reste plusieurs années sans générique parce qu’il y a eu beaucoup de recherche… beaucoup de travail pour pouvoir trouver ces molécules, trouver ce médicament et que… au bout de plusieurs années… pas ils sont rentrés dans leurs frais, enfin ils ont réussi à retrouver… tout l’argent qu’ils ont mis dans la recherche, ils l’ont retrouvé, ils peuvent continuer de rechercher, c’est là qu’il y a certains autres… autres maisons… laboratoires, qui eux ne font pas de recherche… mais permettent à ce que les prix, le médicament se retrouve sur le marché avec un coût normal, enfin plus normal, qui ne compte plus les recherches parce que la recherche a déjà été rétribuée…

( ?) moi j’ai l’impression que pour beaucoup, le médicament, en fait l’original, justement est l’original, est le meilleur, donc il y a que des copies après… mais ils se rendent pas compte en fait que s’il est plus cher… aussi parce que pour beaucoup de gens quand c’est plus cher, c’est mieux… je sais que pour beaucoup de gens, par exemple dans l’alimentation, payer un peu plus cher, ils pensent que la qualité sera de toutes manières meilleure, ce qui n’est pas forcément le cas. J’ai l’impression qu’il y a beaucoup de gens qui se disent l’original il est plus cher, mais il est aussi meilleur… il est plus cher parce que c’est le meilleur, le mieux, qu’un copie donc voilà. Ils se rendent pas compte qu’il est plus cher parce qu’il y avait eu cette recherche, voilà c’est pas les ingrédients qui sont les plus chers, c’est le travail qui l’a rendu plus cher et que celui qui est moins cher, il y a pas eu tout ce travail là… que l’ingrédient après il reste le même, donc le même coût, mais il y a pas le savoir.

(explication reçue ?) non, je crois pas qu’on m’a expliqué ça au début… c’est petit à petit… non, au début on m’a dit qu’ils étaient moins chers ! mais on m’a pas expliqué pourquoi… ça c’est moi… ! (qu’est-ce qui vous a convaincue vous ?) ce qui m’a convaincue c’est de me dire qu’il était moins cher et que mon médecin me dise qu’il était le même, ça m’a suffi, moi… moi la parole du médecin… je peux pas concevoir de me faire soigner si j’ai pas totale confiance dans la personne qui me soigne ! c’est lui qui a le savoir de ce côté-là donc si lui me demande que c’est bon, moi ça me suffit… mais je sais que ça va pas suffire à tout le monde.

**Patient 9**

(Vous voyez ce que c‘est un médicament générique ?)

C’est un médicament de substitution mais qui n’est quand même pas le même parce que les composants il y a une différence autrement ils auraient le même nom et puis…

(D’accord. Donc les composants, quels composants ? Qu’est ce que vous entendez ?)

Ben les matières qu’on emploie pour fabriquer le médicament il doit y avoir une différence quelque part

(Donc pour vous, il y a quoi comme matières dans un médicament ?)

Ben il y a certainement ben tout dépend du médicament déjà et puis je ne sais pas je ne me suis jamais vraiment renseignée, mon grand-père était pharmacien j’sais pas mais il y a beaucoup de choses il doit y avoir des composants chimiques (oui, oui, d’accord, ok), je suppose mais à part ça…

(Disons par exemple, le Panadol c’est le médicament qui était le premier donc c’est le princeps et puis le Dafalgan, peut-être que vous avez eu ?)

Oui, ça j’ai eu

(Qui est le générique, un des nombreux génériques.

Pour vous, quelle différence y aurait entre le Panadol et le Dafalgan ?)

J’ai pris les deux alors je vous dirais que de mon point de vue il n’y en a pas parce qu’ils ne m’ont pas fait d’effets ni l’un ni l’autre mais je suppose qu’il y a une différence parce qu’autrement ils devraient avoir le même nom mais par exemple moi je vais vous parler du Ponstan

(D’accord)

C’était quoi Méfénacide ?

(Voilà, exactement)

Alors ça ne me fait pas les mêmes réactions

(Non ?)

Non

(Quelles sont les différences ?)

C’est-à-dire que moi je réagis mieux au Ponstan qu’au Méfénacide, Méfénacide ça ne me fait strictement rien tandis qu’un Ponstan ça m’aide

(D’accord, donc ça vous diminue les douleurs ?)

Voilà oui, oui mais alors ce sont des autres douleurs ce n’est pas au niveau cervical c’est quand j’ai eu mal partout

(D’accord, alors que le Méfénacide ne fait pas du tout d’effets sur les douleurs ?)

Non

(Est-ce-qu’il fait des effets indésirables ?)

Pas que j’ai remarqué j’en ai pas pris assez pour en avoir

(Mais le Ponstan vous fait des effets indésirables ?)

Non, non plus j’ai très peu d’effets indésirables en fait je suppose

(Donc, pour vous un générique ça ne contient pas la même substance ?)

Pas tout à fait

(Pas tout à fait, d’accord , ok.

Et puis, alors la question suivante est-ce-qu’il est toujours aussi efficace que l’original ?)

Ben ça dépend, mais moi je crois que tout dépend de la personne

(D’accord)

Parce que l’on peut peut-être mieux réagir à un générique qu’à un autre mais faut essayer

(D’accord, ok. Puis les effets secondaires ?)

Ca doit être la même chose

(Ca doit être la même chose, d’accord. Vous pensez que le médicament princeps et le générique sont soumis aux mêmes contrôles par les autorités d’enregistrement ?)

J’espère

(D’accord)

Je suppose je n’en sais rien j’avais posé la question mais je suppose que oui, peut-être moins parce qu’ils sortent plus vite il a peut-être moins d’essais

(D’accord. Est-ce-que vous pensez qu’il est plus facile ou plus difficile d’avaler un générique que le médicament princeps ?)

C’est la même chose

(C’est la même chose pour vous)

Pour moi

(Vous vous souvenez, vous avez donc le Dafalgan, le Méfénacide ?)

Pour moi c’était la même chose

(Vous savez qui les fabrique ? Les génériques)

Mais les sociétés ça doit être les mêmes, elles gagnent des deux côtés

(D’accord, ok)

(Est-ce-que vous hésiteriez à prendre un générique ?)

Non mais dans mon cas peut-être plus j’hésiterai à changer, parce que chaque fois que je change quelque chose il faut tout remettre et alors j’aime pas changer parce que ça recommence, plus de douleurs, plus de…parce qu’il faut de toute façon que je me réhabitue à ce que je prends et puis ça peut être bon comme ça peut-être mauvais mais je n’ai pas changé beaucoup, je peux pas vous dire, vous vous savez peut-être si j’ai du prendre des génériques… à part Ponstan et Méfénacide c’est tout ce que je me souviens

(L’Inderal, vous avez l’Inderal autrement la méthadone, il n’y a pas ça non plus et puis)

Metfine ?

Non j’sais pas

(Pardon ?)

Le Metfine, non…

(Oui, là c’est un générique oui)

Mais je n’ai jamais essayé que ça donc je peux pas vous dire je maigrirais peut-être plus

(Vous avez eu un antidépresseur, je ne me souviens plus lequel ?)

Oui, ah moi non plus

(Le Triptyzol, vous avez eu ?)

Non

(Le Saroten ?)

C’était quoi encore, c’est loin ça

(Bon c’est égal, ok)

Je ne sais plus mais je crois j’ai du changer

(Oui on avait essayé deux mais je crois que c’était des molécules différentes. D’accord, donc en fait vous n’hésiteriez pas plus de prendre un générique que de prendre un autre ?)

Non

(D’accord)

(Comment vous avez entendu parler des génériques ?)

Comme tout le monde je crois par les journaux tout ça à la pharmacie souvent mais moi il ne m’en propose pas beaucoup je dois dire puisqu’il n’aime pas changer, il change jamais ce qu’on me donne quoi… ils ne m’ont jamais présenté autre chose, pour mon mari peut-être mais lui il est contre

(Il est contre les génériques ?)

D’avance, il n’est pas du tout comme moi et puis…

(Votre médecin vous en a parlé ?)

Je ne me souviens pas non pas vraiment on n’a pas vraiment discuté de génériques quoi il m’a peut-être dit je vous prescris le générique ça c’est possible mais bon moi je ne dis jamais rien avant d’avoir essayé parce que c’est moi qui doit tester et puis c’est pas en lisant ce qui est dedans que je peux dire si ce sera bon ou mauvais pour moi parce que je n’y connais rien donc

(D’accord. Vous en avez entendu parler à la télévision ?)

Oui, ça oui, télévision les journaux à la pharmacie

(C’est des panneaux publicitaires ?)

Oui peut-être certainement disons on voit et on ne fait même plus attention parce que qu’en on connait ben c’est fini quoi on connait

(D’accord. Vous avez l’impression que vous en avez entendu parler en bien, en mal ?)

Un peu des deux, à la pharmacie toujours en bien parce que je suppose qu’ils sont presque obligés de vous le proposer, un moment en tout les cas c’était ça, y a eu un moment ou ils essayaient de proposer mais moi je disais non vous ne changez pas c’est le médecin qui change si jamais parce que ça je veux pas commencer à faire des essais avec euh surtout ce sont des jeunes en général donc euh et puis on n’insistait pas franchement, mais je crois qu’ils étaient plus obligés il y a quelque temps que maintenant ou alors c’est parce qu’elles me connaissent bien et puis qu’elles ne me le proposent plus ça c’est possible…euh chez les gens y a des deux comme mon mari qui est contre mais on n’en discute pas vraiment, mais lui il est contre les médicaments il n’aime pas les médicaments il doit en prendre pour l’emphysème et ça lui suffit alors il prend aussi ce qu’on lui a donné, je suis certaine qu’il a des génériques mais qu’il le sait pas donc je n’en parle pas puisque je dis qu’il est contre autant pas lui dire

(Vous savez pourquoi il est contre ?)

Il est assez comme ça parce qu’il a décidé qu’il était contre et puis ben moi j’en ai pas beaucoup parlé parce que je parle que de moi en fait je ne pense qu’à moi j’irais jamais conseiller à quelqu’un quelque chose parce que ce n’est pas à moi à le faire… ce que je puis dire c’est si on me dit tu as pris ça c’était bon ? je peux dire oui à moi ça fait de l’effet mais c’est tout et puis c’est rare que j’ai jamais parlé de médicaments j’crois pas

(D’accord)

(Est-ce-que vous avez été chercher des informations sur les génériques sur internet par exemple ?)

Non ça non franchement

(Et puis est-ce-que vous allez parfois sur internet pour.. ?)

Oui je vais mais pour voir la maladie plus que les médicaments ça je vous fais confiance ben oui parce que je n’y connais rien donc y a tellement on nous dit tellement de choses sur internet qu’on se perd encore plus et puis je fais confiance pour l’instant je n’ai besoin de rien d’autre

(Vous êtes allée chercher quoi sur internet ?)

Ben j’été aller voir l’emphysème ce que c’était et puis la syringomyélie pour avoir un petit peu de complémentaires parce que le professeur m’avait bien dit mais on oublie…mais bon j’ai pas insisté plus que temps parce que j’ai bien vu qu’il y’avait beaucoup de choses mais c’est pas toujours exacte bon mais ou alors y a tellement de choses qu’on mélange un petit peu quoi

(Comme ça par curiosité, comment vous allez chercher ?)

Je mets le nom de la maladie

(Donc syringomyélie sur google par exemple ?)

Euh je suis sur quoi oui sur google je suis et puis euh je suis pas très bonne et puis ce qui sort et puis là je regarde un petit peu comme ça…mais j’ai plus regardé pour l’emphysème que pour la syringomyélie par contre là j’avais vue qu’on pouvait faire un vaccin pour mon mari puis j’en ai parlé au médecin et il lui a fait le vaccin

(Ah d’accord, donc ça quand même eu une influence une fois ?)

Mais c’est la seule chose, oui une fois y a eu de l’influence j’étais plus rapide

(D’accord très bien ok)

(Donc votre médecin vous a déjà prescrit des génériques ?)

Euh Méfénacide oui

(D’accord et il vous avait dit que c’était un générique ou bien vous ne vous souvenez plus ?)

Je ne me souviens pas…oui je suppose parce qu’avant j’avais du ponstan puis il m’a dit ah oui on va donner ça…bon j’ai supposé mais il m’a pas dit c’est un générique ni pourquoi

(Pourquoi il changeait ?)

Mais je ne suis pas très curieuse je vous dirais

(D’accord oui d’accord. Donc il ne vous a rien dit de particulier vous vous avez accepté ?)

Moi j’ai pris et puis j’ai remarqué que ça faisait moins d’effets et je lui ai dis et la fois suivante il m’a represcrit du Ponstan en mettant que c’était celui qu’il fallait me donner

(D’accord donc vous n’avez pas eu de problèmes avec votre assurance ?)

Non non

(Votre pharmacien vous a déjà proposé de substituer le médicament original ?)

Mais là j’avais toujours dit non sauf si le médecin

(La Metfine c’est votre médecin qui vous l’a prescrit ?)

Dés le départ je n’ai pris que ça oui……et puis là j’ai perdu tout de suite mais après je ne perds rien c’est dommage

(Donc vous n’avez pas fait de modifications proposées par le pharmacien ?)

Non

(Que le médecin n’aurait pas…)

Non je n’oserai pas je vous dirais franchement parce que j’ai trop peur dés j’avais remarqué que dés qu’on change un petit quelque chose moi ça recommençait tout le processus donc j’ai pas envie

(D’accord. Si par exemple pour un des médicaments que vous prenez s’il existait un générique, vous aimeriez qu’on vous le propose d’office ou bien ?)

Non parce que j’aurais peur de devoir tout recommencer (d’accord) j’ai plus envie d’avoir mal comme j’ai eu mal ça non ça franchement ça m’arrive encore mais disons c’est plus ou moins géré je sais comment le gérer tandis que là il faudrait recommencer trouver des autres solutions ça j’ai plus envie c’est fatigant ça ça fatigue au bout des années on en a je crois que c’est ça aussi dés qu’on avale et puis tant que ça marche ça marche et puis on verra si ça marche plus alors là d’accord, si je vois qu’il y’a plus d’effets par exemple ou bien alors là je suis d’accord de changer parce que…

(Si par exemple on vous prescrivait un antibiotique, vous seriez d’accord qu’on vous prescrive d’office un générique ?)

Oui

(D’accord. C’est vraiment pour la douleur que vous faites très attention, pour le reste diabète vous avez accepté ?)

Oui oui ben de toute façon comme je vous le dis je ne suis pas curieuse je ne le demande pas et en général mon médecin ne me dit pas c’est un générique je crois qu’il me connait donc il me dit rien et puis si ça marche ça marche

(D’accord)

Il sait que je suis comme ça si ça marche ben que ce soit n’importe quoi parce que dans le fond moi c’est le résultat qui compte

(Ca c’est sur, ok)

Je crois que tous les malades ça devraient être ça

(Donc vous avez eu une expérience personnelle avec un antalgique et puis donc c’était le médecin qui vous a proposé en tout cas le méfénacide mais le Dafalgan qui vous l’avais proposé ?)

C’était lui aussi

(D’accord, il vous avait dit pourquoi ?)

Non c’était ben on avait commencé comme ça et puis…

(D’accord parce que vous aviez eu du Panadol avant ?)

Oui je crois que le Panadol j’avais pris moins et puis quand j’ai demandé qu’il me prescrive il m’a mis Dafalgan en me disant c’est la même chose et puis voilà…si je me souviens

(Donc le Panadol vous aviez été l’acheter sans ordonnance ?)

Oui sans ordonnance mais avant que je sois malade déjà

(D’accord)

Une fois quelqu’un m’avait dit prend un Panadol parce que je n’avais pas d’aspirine parce qu’en général je prends de l’aspirine pour ces choses-là

(D’accord, que vous achetez sans ordonnance ?)

Sans ordonnance oui

(Est-qu’on vous a proposé un générique de l’aspirine à la pharmacie ?)

Non

(Non, jamais ? Donc vous avez toujours pris de l’aspirine ?)

Non parce que je prends ma boite puis je la paie donc

(Ah oui, vous vous servez vous-même)

C’est comme ça

(Elles sont vraiment devant le comptoir)

Maintenant oui ou je demande vous me donnez cette aspirine-là mais ils ne me proposent pas autre chose comme on paie nous même je suppose que là ils nous proposent pas

(Oui tout à fait d’accord)

(Donc vous avez remarqué que l’effet de certains n’est pas pareil ?)

En tout les cas pour moi

(Pour vous, mais les effets indésirables c’est la même chose ?)

J’en ai pas beaucoup moi ou alors si j’en ai, je les mets sur le compte de mon état et pas nécessairement sur un médicament puis je me dis ben tant pis il faut y aller comme ça quoi si on me fait du bien pour ce que je recherche j’accepte les effets indésirables

(D’accord)

Par contre maintenant je transpire énormément alors depuis que je suis venue vous voir j’sais si c’est mais c’est effrayant j’ose pas me tourner dans le lit déjà j’ai remarqué c’est pour ça que je me demande si c’est pas mon cœur

**Patient 10**

(Comme je vous ai dit on va parler un peu des génériques. Il s’agira dans votre situation de me faire part de votre point de vue, de votre expérience. Ce qui nous intéresse c’est vraiment votre point de vue, comment vous voyez les choses, ce que vous en avez compris dans votre situation, et par rapport aux douleurs que vous ressentez. Dans votre cas si j’ai bien compris, vous souffrez de fibromyalgie. C’est bien ça ?

Oui tout à fait

- (Alors ce sera en référence à votre problématique, comme vous le voyez y’a pas de bonne ou de mauvaise réponse, ce qui nous intéresse c’est vraiment votre avis. Alors ma première question ça va être de vous demander ce qu’est à votre avis un générique.)

- Pour moi un générique est un médicament qui a été commercialisé par un laboratoire pendant x années et qui est repris sous un autre nom. Voilà.

- (Ok. Ca veut donc dire, si je vous demandais, est-ce que c’est la même chose que l’original ?)

- Alors là je le pensais, et suite à des visites chez les médecins, certains m’ont dit que ce n’était pas exactement le même médicament, que c’était en effet la même molécule mais que les deux n’étaient pas tout à fait identiques. Mais c’est l’avis de certains médecins, là je ne sais pas

- (Qu’est ce que vous en pensez vous ?)

- Vis a vis des génériques, j’ai pas eu de euh.. je veux dire je les ai supportés, pour moi c’est les même médicaments. C’est les médecins qui me mettent sur une fausse piste, je ne sais pas pourquoi.

- (Donc pour vous c’est la même chose ?)

- Oui mais suite à une visite chez le médecin je me suis posé des questions.

- C’était pour quel médicament que il y avait un doute, quel type de médicament ?

- C’était relatif à ma maladie… je sais plus lequel c’était…bon c’était un médecin très porté sur l’homéopathie, …c’était pour quel médicament… un antidépresseur…ah oui c’était un générique du prozac, voilà c’était ça. Il m’avait dit qu’il valait mieux prendre le prozac que le générique du prozac…alors je ne sais pas.

- (Et qu’est ce que vous en avez pensé ?)

- Bah je me suis interrogée. Je suis allée chez mon médecin et lui a dit ce que m’avait dit l’autre médecin. Il m’a dit « bah si vous voulez on vous donne du prozac au lieu du générique du prozac. De toute façon comme j’ai changé de médicament voilà

- (Donc de votre point de vue, est-ce que le générique est aussi efficace que l’original ?)

- Pour moi oui, pour moi oui, je vois pas pourquoi on nous raconterait des histoires.

- (Donc, vous avez l’impression qu’on nous dit que les génériques c’est la même chose et que pour vous c’était la même chose jusqu’au moment où ce médecin a évoqué un doute.)

- Voilà ouais, mais moi ça m’empêche quand même pas de prendre des génériques.

- (Quelles sont les raisons pour vous d’utiliser des génériques ?)

- Bah moi si on me le conseil, c’est que y’a une raison. Voilà, ne serait-ce que financièrement par rapport à la Sécu, parce que je suis en France, voilà si on me les conseille c’est qu’il y ‘a une raison. Alors je suis pas qualifiée dans le domaine pour savoir la raison exacte, mais bon j’écoute, je suis bête et disciplinée, je me pose pas trop de question.

- (Est-ce que pour vous y’a les mêmes effets secondaires avec un générique ?)

- Oui je pense, oui.

- (Donc ça ne fait pas de différence ni en termes d’effets attendus ni en termes d’effets secondaires.)

- Non, pour moi non.

- (Ce sera du même ordre, d’accord. Est-ce que vous savez qui les fabrique ces génériques ?)

- Non.

- (Vous pensez qu’ils sont contrôlés comme les originaux ?)

- Oui, ce que je prendrai pas par contre, ce serait des médicaments qui viennent d’autres pays que j’achèterai sur internet, ça je prendrai jamais.

- (Quelles sont les différences pour vous entres les génériques et les médicaments que vous trouvez sur internet ?)

- Bah les génériques ça passe par une pharmacie, alors que sur internet on peut vous vendre n’importe quoi, voilà. Moi y’a des choses que j’achèterai jamais par internet, dont les médicaments. Une pharmacie pour moi c’est une référence.

- (C’est une référence par rapport à quoi ?)

- Bah une référence, par rapport à leurs études, à leur métier, par rapport à leur euh...il sont quand même, ils sont pas comme les médecins, mais ils ont certaines obligations, donc on doit les croire comme les médecins.

- (Donc en somme, selon vous on doit distinguer les copies comme les génériques…)

- Pour moi les génériques ne sont pas des copies. Parce que un générique est vendu dans une pharmacie, et n’est pas vendu sur internet. Enfin j’ai jamais cherché, mais je ne pense pas.

- (D’accord. Donc en fait, sur internet, ce serait des copies dans le sens d’une fraude si je comprends bien, ou d’une contrefaçon ?)

- Ou d’une contrefaçon plutôt, ouais ouais, je verrai ça comme ça, mais pas un générique. Pour moi un générique c’est l’identique.

- (Donc ce serait la même chose que le médicament avec un autre nom, versus les copies sur internet qui seraient des versions abusées.)

- Oui oui pour moi oui.

- (Ok, est-ce que vous pensez qu’il y a des problèmes quelconques à l’utilisation des génériques.)

- Je n’en vois pas, je n’en vois pas

- (Ni en terme de leur substance, ni en terme d’aspect ou de taille ?)

- Non non, je ne vois pas de différence

- (Vous même est-ce que vous auriez des difficultés à prendre un générique ou est-ce que vous prendriez un générique sans aucune difficulté)

- Alors jusqu’à ce que j’ai la maladie, sans aucun problème, mais maintenant j’hésiterais plus, parce que j’ai tellement de douleur que j’ai peur d’avoir un changement…vous voyez, mais c’est pas rapport à ma maladie

- (Quand vous dites que vous avez peur d’avoir un changement, vous pourriez préciser ?)

- Alors un changement, quand à l’effet euh…voilà, parce que jusqu’à maintenant les médecins m’ont jamais prescrit de générique, ils m’ont toujours prescrit les médicaments.

- (Alors avant la maladie, vous auriez accepté sans vous poser la question ?)

- Mais j’en ai pris

- (Vous pensiez à quel type de médicament qui vous poserait problème ?)

- Par exemple un médicament que je prends à base de dérivé morphinique, parce que là les dérivés morphiniques entrainent déjà des effets secondaires, alors je me dis que si déjà les effets secondaires sont plus importants, ce serait plus dérangeant pour moi, voilà. Dans l’antidépresseur que le prends, le Ixen, on dit qu’il y’a une molécule qui est bonne pour la fibromyalgie, donc moi je prendrai pas un générique puisque voilà…

- (D’accord, donc pour votre maladie, pour la fibromyalgie, vous ne prendriez pas de générique ?)

- Non.

- (D’accord, même si c’était la même substance ?)

- Oui parce que avec cette maladie j’ai beaucoup trop de doute étant donné qu’il y’a pas de traitement pour ça, alors je me dis autant rester dans le système et pas me poser de questions supplémentaires, j’ai assez de problème comme ça pour m’en créer d’autres, c’est surtout cet aspect là.

- (D’accord.)

- Pour pas me rajouter un souci une question, si un jour je suis vraiment très mal, si c’est le médicament, voilà, je veux pas m’ajouter de problème.

- (Donc un générique c’est la même mais, y’a des circonstances où on peut préférer l’original ?)

- Bah disons, les médecins ils nous donnent l’original. Donc voilà, si le médecin me donnait le générique, bah je prendrai ce que le médecin m’a donné voilà. Mais jusqu’à maintenant ils m’ont pas donné le générique.

- (Il y’a peut-être des médicaments que vous prenez pour lesquels il y’a pas de générique hein, ça peut aussi arriver. Est-ce qu’il y’a d’autres circonstances où ça ne vous poserai aucun problème de prendre un générique ?)

- Oh oui, moi je vous dit avant d’être malade, quand j’allais à la pharmacie, on me disait « vous voulez un générique », je disais « oui sans problème », je n’ai jamais refusé un générique.

- (Mais c’est depuis votre maladie que les choses ont un peu changées ?)

- Oui.

- (Vous avez ce problème depuis quand ?)

- 1 an et demi.

- (D’accord. Ok et vous prenez quoi comme médicaments en ce moment ?)

- Euh j’ai mon ordonnance, je vais vous dire… alors je prends de (11 :16) du l’Ixen, du Laroxyl, du Topalgic, du…euh alors attendez…j’ai du Xprim… voilà c’ est ce que je prends.

- (Ça vous aide ?)

- Oui oh oui oui, et j’en prends un autre le soir, du Tétrozépam, un nom comme ça…

- (Hum hum, pour mieux dormir ?)

- Oui. Ah oui et je prends aussi du Zolpidem, mais par contre je crois que le Zolpidem c’est déjà un dérivé du Stilnox, mais je ne suis pas sûre. Voilà ce que je prends tous les jours.

- (D’accord, donc vous avez quand même une expérience des médicaments contre la douleur qui est importante. Est-ce que quand vous prenez un médicament, comment vous faites pour vous renseigner sur ce médicament ?)

- Je me renseigne pas. Non. Quand je rencontre des personnes, des amis qui ont cette maladie, je leur demande ce qu’elles prennent. C’est tout.

- (Donc vous ne lisez pas la notice ?)

- Ah si, je lis toujours la posologie, pour les effets secondaires.

- (Donc la petite notice qui est avec les médicaments vous lisez toujours ?)

- Oui, toujours la posologie. Parce que j’ai eu quelques surprises certaines fois et maintenant je le fais systématiquement, oui. Je regarde la posologie parce que je me dis si on a des effets secondaires, bah voilà, il faut faire une relation avec les médicaments.

- (Est-ce que ça vous est arrivé d’aller chercher des informations sur internet propos des médicaments ?)

- Non, Non jamais.

- (D’accord. Si vous preniez un générique, vous liriez aussi la notice d’accompagnement ?)

- Oui, oui, la posologie surtout euh je veux dire, la composition je ne regarderai pas, mais je regarderai toujours les effets secondaires oui.

- (D’accord, c’est le plus important pour vous dans…)

- Pour moi oui.

- (Dans la notice)

- Oui.

- (D’accord. Est-ce que votre médecin vous a déjà prescrit des génériques ? Vous m’avez dit qu’à la pharmacie il est déjà arrivé qu’on vous en propose, mais est-ce que votre médecin vous a déjà une fois ou l’autre pour une raison ou pour une autre, prescrit un générique ?)

- Euh non.

- (Non)

- Non c’est souvent la pharmacie qui demande, mais pas le médecin.

- (Et la pharmacie vous a donc déjà proposé une fois ou l’autre un générique, vous vous souvenez pour quel type de médicament ? Ou pas du tout ?)

- Je crois que c’était un médicament pour dormir, mais j’en suis pas sûre.

- (Et comment vous avez réagi à ce moment là ?)

- J’ai pas eu de problème, je veux dire oui, pour moi ça n’a pas fait de différence.

- (Et ensuite il y a eu cette proposition dont vous m’avez dit qu’on vous a proposé le générique du Prozac ?)

- Oui.

- (Et là vous avez réagi comment ?)

- Bah je l’ai pris. Et c’est après quand je suis allé voir un autre médecin qu’il m’a dit « il faut surtout pas prendre de générique parce que ce n’est pas la même composition que le vrai médicament » et sur la base de quoi c’est là où j’ai eu voilà ça m’a posé un doute, et je me pose toujours la question d’ailleurs, pourquoi ce médecin dit ça ?

- (Mais ça c’était pas votre médecin n’est-ce pas, j’ai bien compris ?)

- Non, non non c’est un médecin que j’étais allé voir pour la fibromyalgie parce qu’il faisait de l’acupuncture des choses comme ça, mais ce n’est pas mon médecin traitant.

- (Vous avez pu parler avec votre médecin traitant de cette histoire de ce doute ?)

- Non, mais par contre comme je vais régulièrement, parce que je fais de la mésothérapie toutes les semaines, je pourrai lui poser la question oui.

- (Ça vous a fait du souci cette, ce doute qu’il vous a mis ?)

- Oui, oui ça m’a dérangé. Oui, parce que je me dis, pourquoi un médecin dit ça ?

- (Vous avez imaginé quelque chose de précis ?)

- Bah j’ai imaginé que dans les gélules évidemment il y’avait la molécule, mais comme y’a pas que la molécule, ils auraient modifié un autre ingrédient, je sais pas, mais qui couterait moins cher, tout ça pour réduire les coûts.

- (Ça voudrait dire que ce serait de moins bonne qualité ?)

- Oui.

- (Donc en somme ça vous a mis un doute sur la qualité…)

- Des génériques.

- (Des génériques oui. Et donc à partir de là ça vous encouragerait moins à en prendre.)

- Pour ce traitement là oui.

- (Et c’est donc à partir de ça aussi que vous pour soyez pour le traitement de la fibromyalgie, que vous soyez très attachée à ce qu’on ne vous change pas les …)

- Oui je veux garder ce que j’ai oui.

- (D’accord, bon si ça fonctionne bien, y’a pas..)

- Oui on passe tellement de moments difficiles.

- (Sachant à quel point c’est difficile de trouver un traitement hein.. D’accord…euh…donc…pour votre traitement de la fibro, est-ce que vous savez si il y aurait un générique ?)

- Non, je ne sais pas, j’en ai jamais entendu parler. Même à la pharmacie, ils me proposent pas, ils me donnent toujours le médicament que me donne le médecin.

- (Et vous vous n’auriez pas aimé qu’on vous en propose ?)

- Ah non

- (Comme vous avez dit là, sur ça on touche pas.)

- Voilà, par contre sur d’autres maux que j’aurais pourquoi pas.

- (Si vous aviez une grippe ou…)

- Oui là j’hésiterai pas, non.

- (Est-ce que là vous auriez le même type de doute…je vous embête un peu là…mais est-ce que vous auriez le même type de doute sur le fait que c’est la même substance mais un produit d’accompagnement moins cher, et de moins bonne qualité, comme vous disiez ?)

- Bah il faudrait que je connaisse exactement la composition, de visu, et que ce soit marqué la même chose.

- (Vous voyez, je vous pose toutes ces questions parce que, comme vous le dites très bien, cette remarque de ce médecin a insinué un doute…)

- Oui

- (… et vous dites peut-être que on met quelque chose d’un peu moins bien dedans pour diminuer le coût donc c’est d’un peu moins bonne qualité. Donc moi je me dis en vous écoutant, mais si vous aviez un autre problème de santé ou une infection, et qu’on vous proposait un générique, est-ce que il n’y aurait pas le risque que vous vous disiez, « ah mais est-ce que c’est d’aussi bonne qualité que si j’avais l’original » vous voyez ?)

- Bah non, en dehors de ma maladie, je me dis bah pourquoi pas essayer, si ça me convient ça me convient.

- (Donc là vous n’auriez pas ce souci là ?)

- Non, non, je ne l’aurais pas, mais par contre par curiosité je regarderais la composition. Et puis à la limite je me la ferais expliquer par le pharmacien.

- (D’accord donc là vous demanderiez à ce qu’on vous certifie que c’est vraiment bien la même chose et pas comme on dit parfois une pâle copie ?)

- Voilà oui.

- (Parce que à vous entendre j’ai l’impression que on pourrait pour certains de ces génériques dire que ce n’est pas simplement une copie mais une pâle copie, est-ce que c’est juste ?)

- Non, pas pâle copie quand même. Non y’aurait une modification dans la composition qui modifierait non pas le médicament, mais le coût du médicament puisque en fait il est vendu moins cher. Si il est vendu moins cher ce n’est pas à mon idée à cause de licence – je dis licence comme ça – mais que y’a quelque chose qui change quand même le coût voilà.

- (Est-ce que vous même vous avez déjà pris des génériques contre une douleur quelconque ?)

- Non.

- (Ni en terme de paracétamol, comme le Dafalgan ou autre, ce genre de médicaments, et bien sûr pas pour votre maladie. Donc vous avez jamais pris d’antidouleurs qui seraient des antalgiques ?)

- Non

- (C’était toujours des originaux. On vous a jamais proposé vous avez dit, et c’est finalement une proposition qu’à l’heure actuelle vous n’accepteriez pas.)

- Pour ce qui concerne ma maladie. Pour d’autres maux oui, pour un rhume tout ça oui j’accepterais.

- (Est-ce que vous connaissez des gens qui ont pris des génériques contre la douleur ?)

- Non, non je connais pas.

- (Est-ce que vous avez l’impression que c’est quelque chose dont on parle beaucoup ou peu ?)

- Peu je trouve. On en parle pas assez, donc y’a un manque d’information. On a l’impression que c’est fait de façon, je dis pas sournoise, mais le médecin vous les prescrit pas, vous allez à la pharmacie et « écoutez ça vous dérange si je vous donne un générique ? ». Pourquoi le médecin le prescrit pas tout de suite ? ça c’est une question pour moi sans réponse.

- (C’est une question importante pour vous ? Pourquoi c’est pas le médecin qui vous le prescrit ?)

- Bah oui, pourquoi il se donne pas la peine de prescrire un générique de suite. Pourquoi c’est au pharmacien de proposer ?

- (A votre avis, pourquoi le médecin ne prescrit pas le générique ?)

- Peut-être qu’il y’aurait des effets secondaires différents, et que le patient pourrait, je dis pas se retourner contre lui, mais pourrait lui dire « bah écoutez pourquoi vous m’avez pas donné le médicament au lieu du générique » voilà, je sais pas.

- (Et puis alors, si on regarde le point de vue du pharmacien, à votre avis, qu’est ce qui ferait que le pharmacien proposerait le générique plutôt que l’original ?)

- Alors je me dis est-ce que c’est pas une question commerciale ?

- (C’est à dire ?)

- Pour réduire les coûts de la sécu en France, ou peut-être qu’ils ont une marge plus importante sur les génériques, je ne sais pas.

- (Quand vous dites « on manque d’information » qu’est ce que vous aimeriez avoir comme information ?)

- Bah du type, euh, à la télévision y’a un slogan qui était arrivé il y’a quelques années maintenant « les antibiotiques c’est pas automatique », pour les génériques on pourrait nous dire « prenez des génériques, c’est la même chose que… » mais là aussi à mon avis c’est une question de coût pour les laboratoires, donc ils peuvent pas. On est en manque d’information moi je trouve.

- (Ça m’intéresse car je crois que là on est vraiment au cœur de ce qu’il se passe avec les génériques, quand vous dites que vous avez l’impression que l’on passe un peu en catimini avec ces génériques, vous pouvez préciser ce que vous entendez par là ?)

- Que c’est quelque chose qui n’est pas entre guillemets officiel, vous voyez, que c’est pas quelque chose qui est approuvé par la médecine en général.

- (Mais si c’est pas approuvé, ça voudrait dire que c’est pas tout à fait le même médicament ?)

- C ‘est que y a une raison. Laquelle je ne sais pas, mais c’est que y a une raison.

- (Moi quand je vous écoute, si je résumais, je dirais que vous avez quand même des doutes sur les génériques.)

- Oui maintenant oui, et c’est ce médecin qui m’a crée le doute.

- (Donc c’est sur cette base là que le doute est venu.)

- Oui oui.

- (Donc si on vous avait pas dit ça à propos du Prozac, vous auriez éventuellement pris des génériques pour votre maladie).

- Oui oui. Exactement.

- (Donc quand vous dites « il faut une information », il faudrait qu’on explique au patient exactement, ce qui est pareil, pourquoi c’est la même, de façon à ce qu’il y ait une version tout à fait officielle.)

- Voilà exactement.

- (Et qu’on ne fait pas les choses en catimini.)

- Voilà comme ça. Là on a l’impression que c’est le pharmacien qui est derrière son comptoir et qui dit en chuchotant « bah écoutez vous voulez pas un générique à la place ». C’est ce sentiment que j’ai. Et ça c’est pas rassurant. Quand j’ai entendu l’autre médecin dire ça, je suis pas rassurée que ce soit les pharmaciens qui le proposent.

- (Donc pour que vous soyez rassurée, il faudrait que ce soit les médecins qui le proposent ?)

- Oui oui.

- (D’accord. Si votre médecin discutait ça avec vous, ce serait une information plus importante ?)

- Oui parce que je lui demanderais quelle différence y’a-t-il ? Expliquez moi la différence, et après c’est à moi de juger. Pas de juger, de me faire expliquer, mais oui.

- (Donc ce que vous aimeriez comme information vous aimeriez en quoi c’est semblable, et quoi c’est différent, et une information donnée par le médecin.)

- Oui.

- (Est-ce qu’il y aurait une autre voie d’information qui vous semblerait appropriée ?)

- Bah non parce que par les médias c’est pas possible, parce que à mon avis y a de trop gros enjeux financiers. Bah oui, pour moi, la meilleure des informations serait par le médecin.

- (Ça vous arrive de chercher des informations de santé sur internet ?)

- Euh je suis allé voir une fois au début de ma maladie et puis j’ai arrêté. Parce que y’a tellement de versions, d’individus qui réagissent différemment, je dis « chacun vis sa maladie à sa façon ».

o BLANC SUR LA CASSETTE DE 27 :02 à 28 :18

- (Votre information c’est essentiellement via votre médecin ?)

- Oui, et le centre antidouleur, oui, les informations c’est là que je les prends oui.

- (Donc pour les génériques c’est info par le médecin sur le contenu, les différences et les effets secondaires ?)

- Oui, pour moi c’est la base.

- (Est-ce qu’il y a d’autre chose pour les médicaments qui sont de votre point de vue importantes à savoir, à dire ?)

- Bah non, je pense que c’est la base, pour moi c’est surtout les effets secondaires le plus important dans un médicament. Parce que en voulant vous soigner avec l’un quelque fois avec les effets secondaires de l’autre vous êtes encore plus malade.

- (Ça c’est votre expérience ?)

- Oui c’est mon expérience, oui une fois j’ai eu un problème d’hypotension, on m’a donné un médicament qui m’a fait avoir un urticaire sur tout le visage. Bon.

- (D’accord, d’où votre inquiétude sur les éventuels effets secondaires des génériques ?)

- Ah oui, bah génériques ou autres médicaments, sur les effets secondaires je suis très précautionneuse.

- (Avant le déclanchement de cette fibromyalgie, vous preniez d’autres médicaments ?)

- Non, non puisque j’ai pas de cholestérol, pas de tension, pas de problèmes. Non des fois je prenais pour dormir parce que j’ai du mal pour dormir. Mais non, pas de médicaments, juste si j’ai un maux de tête de temps en temps ou si j’ai la grippe, mais non pas de médicament, pas de suivi.

- (Donc c’est vraiment une question que vous avez eu à vous poser au déclanchement de votre maladie il y a un an et demi. Les médicaments en général, qu’ils soient génériques ou pas.)

- Oui oui tout à fait.

- (Est-ce qu’il y’a quelque chose que vous souhaiteriez ajouter à ce que l’on a dit là ?)

- Non parce que je pense que vous m’avez parlé donc de l’information, c’est vrai que c’est des médecins qu’elle doit venir pour moi. Voilà. Et puis vous qu’en pensez vous des génériques ?

- Alors écoutez, je vais arrêter l’enregistrement.

COUPURE

MEDECIN : La patiente ajoute encore après que j’ai coupé le micro qu’elle se demandait pourquoi on n’utilisait pas QUE des génériques quand il y en a si c’est la même chose mais qu’on continuait à proposer les originaux alors que l’un des deux est plus cher que l’autre. Et elle insiste encore une fois sur l’importance du rôle du médecin dans la prescription du générique en relevant par ailleurs que ce n’est pas au pharmacien de changer le médicament pour un générique mais que c’est au médecin de proposer un générique. Elle revient encore une fois sur le fait que quand elle a pris le générique du Prozac elle se sentait exactement comme avec les Prozac, les mêmes bénéfices et les mêmes effets secondaires, et que quand ce médecin phytothérapeute lui expliquait qu’il fallait pas prendre de générique jamais, et surtout pas générique du Prozac, elle a eu un doute énorme comme elle l’a dit qui s’est manifesté, en dépit du fait qu’elle avait senti elle qu’il n’y avait pas de différence selon qu’elle prenne le générique ou le médicament original.

**Patient 11**

(A votre avis, qu’est-ce qu’un générique ?)

Bon ça, on doit répondre à une question de pharmacologie (fait une formation de secrétaire médicale) c’est un médicament qui a été fait euh, d’après une longue euh… d’après les originaux, ont été prouvés… ont fait leurs preuves et d’après… avec les mêmes excipients, ils ont fait des génériques.

(Même chose ou différent ?) Personnellement, je dois dire que, pour moi, l’Irfen, c’est différent… parce que, au début, j’avais du Brufen et je ne le supportais pas, alors que l’Irfen je le supporte relativement mieux, mais c’est les effets secondaires qui sont pas marrants, pour les reins.

(où serait la différence pour vous ?) dans certains excipients qu’il peut y avoir ou dans les effets secondaires qu’il peut y avoir.

(pas mêmes ES ?) pas personnellement

(différence ?) que avec le Brufen je faisais beaucoup plus de brûlures d’estomac quel l’Irfen, je le supporte, je fais pas de brûlures d’estomac… et ma fille, ça la faisait vomir, le Brufen, et l’Irfen elle le supporte mieux, sans ces nausées.

(pas mêmes excipients ?) euh… qu’est-ce qu’on a expliqué… si c’est le… la … le médicament est le même mais il y a peut-être certaines petites choses qui changent… oui je crois que c’est ça, l’excipient…

(efficacité ?) c’est pareil, l’efficacité est pareille.

(substance ?) peut-être à quelques petites choses près, oui.

(copie ? juste ?)…..non, je pense quand même pas parce que c’est un médicament en lui-même… non je pense pas que ce soit des copies, c’est… c’est pas une copie… c’est… c’est un bis repetita quoi…

(copie péjoratif ?) oui c’est ça (pour vous, pas de raison ?) voilà tout à fait… mais par contre j’ai du Glucophage et j’avais du Metfin… alors le Metfin, je le supportais pas du tout mais le Glucophage, je le supporte… aussi à cause des effets secondaires… et le Glucophage est plus efficace que le Metfin d’après moi, ça m’a fait baisser plus la glycémie.

(ici original plus efficace – ??) aucune idée, je sais pas… parce que au début quand je prenais du Glucophage, ma glycémie descendait bien, mais avec le Metfin, moins. Mais pourquoi, ça…. aucune idée.

(copie= ??) ouais… ce serait…. Comme un clone qui à chaque passage est moins bien… enfin vous voyez… non, copie c’est effectivement quelque chose qui joue pas vraiment, alors que le générique est… ouais, est un bon médicament.

(qui les fabrique ?) …. C’est une bonne question… ben, c’est d’autres … d’autres fabriques… d’autres industries de la pharmacie

(contrôles ?) j’espère… parce qu’alors là, on n’a qu’à se tirer une balle dans la tête… si on peut plus avoir confiance dans les médicaments, c’est grave.

( ?) parce que bon, j’ai des douleurs d’arthrose, enfin des douleurs générales dans le dos, dans les lombaires depuis 52 ans, j’ai des malformations, j’ai des tas de problèmes d’arthrose depuis le pied jusqu’à la nuque… et je sais que même si on ne peut pas guérir, ça peut soulager, un anti-inflammatoire peut soulager quand même.

(avantages génériques ?) bon, de toutes façons, avec le prix… quoique qu’avec le glucophage maintenant il y a pas beaucoup de différence entre le Metfin et le glucophage, mais c’est vrai que les omeoprazide, omeoprazole, comparé au Nexium, ça fait quand même une sacrée différence de prix. Je pense que c’est surtout pour ça puisque les assurances préfèrent qu’on prenne un générique qu’un original.

(moins cher ?) parce que d’après ce que j’ai compris, euh… c’est sur le prix de la recherche qu’on effectue sur un nouveau médicament, le prix de la recherche vient plus cher alors que le générique a déjà été testé, fait ses preuves, été testé et puis il y a que la fabrication… si mes souvenirs sont bons. [Elle s’intéresse beaucoup au ‘médical’, était aide-soignante, fait formation pour secrétariat médical, ses sœurs et sa fille sont aides-soignantes]

(Qu’est-ce qui vous ferait hésiter?) …. Non, je pense pas… il faut voir les effets secondaires éventuels qu’il peut y avoir, mais en principe, non.

(MD a prescrit génériques ?) oui…. ( expliqué?) non, ça je le savais d’avance.

(refus, doute ?) non, à part le Glucophage.

(pharmacien ?) oui… le Glucophage effectivement… il a proposé le Metfin, j’ai dit non, que je supportais pas le Metfin. [diabétique depuis des années]

(comment vous renseignez-vous ?) je lis… ben j’ai loué à la bibliothèque un gros bouquin avec les médicaments génériques parce que ça m’intéresse toujours de comparer… bon, c’est le prix aussi qui est attrayant euh… bon, de toutes façons c’est pas moi qui paie mais les assurances font une grosse tête si on prend l’original, donc je me renseigne surtout sur la littérature qu’il peut y avoir sur les médicaments.

( ?) aussi les posologies [=les notices]

(internet ?) ça m’est arrivé, oui, sur le compendium… ( ?) bon, je l’ai pas chez moi, mais quand je suis au cours, ça m’arrive de regarder internet. ( ?) une fois qu’on a pigé le truc, c’est simple. ( ?) avec le livre, parce que bon ça fait pas longtemps que j’utilise internet et pi je m’y ferai jamais vraiment. (autres infos que médics ?) non, j’ai pas encore eu le temps et puis je sais pas vraiment comment le faire, pi j’ai pas envie de demander.

(génériques la première fois ?) ça fait longtemps, mais… justement à cause du Glucophage, ça fait longtemps qu’on en parle… (souvenir ?) non, mais ça m’a pas gênée plus que ça, je me suis même pas posé la question, je me suis dit, si c’est un générique, ça doit être un bon médicament.

(quoi dire aux patients pour leur expliquer ?) les génériques ? parce c’est vrai qu’il y en a des qui sont crochés sur leurs médicaments… oui c’est pas évident ! en leur disant que c’est la même chose, alors pourquoi pas prendre les vrais médicaments… sur la question prix, ‘de toutes façons, c’est pas moi qui paie, c’est l’assurance’… euh c’est difficile de convaincre quelqu’un qui est couché sur quelque chose…

(autres médicaments génériques utilisés ?) je sais pas si l’Oxycontin est un générique maintenant ? … donc il y a l’omeprazole, et le Dafalgan, co-Dafalgan, que j’ai utilisé il y a… le Zoldorm et puis l’antidépresseur… je sais plus le nom…. Je sais plus le nom, mais je crois que c’est aussi un générique…. à part les nausées du matin, c’est la même chose que ce soit un générique ou pas…. ( ?) non, j’ai essayé que le générique. ( ?) si je le prends le jour, évidemment que je serai mal fichue, que j’aurai des nausées, bon ça passe, d’accord, mais quand on doit faire des cours, c’est pas amusant de dormir ou de courir aux toilettes ! c’et pourquoi je le prends le soir, mais on m’a dit de pas le prendre le soir, que ça éveillait, donc je le prends quand même parce qu’il me semble que… ah oui !avec le Zoldorm, c’est le générique du Stillnox. Mais le Stillnox que j’ai pris à l’hôpital, je le supportais pas du tout, ça ne me faisait pas dormir et le matin, ça me rendait malade, alors que le Zoldorm ça va.

[fait la liste de ses médicaments, dont l’AD dont elle ne se souvient plus du nom et des gouttes de Surmontil le soir pour dormir] C’est sûr que tous ces médicaments là n’auront pas grand effet si on a la tête pleine de négatif… si on a des pensées joyeuses, oui ça peut aller, c’est psychologique, mais si on en a ras le bol, de la dépression, du… on pourra essayer ce qu’on veut, on pourra pas dormir [xxx]. Pour l’antidépresseur… c’est vrai que j’ai plein de problèmes, je suis dépressive, mais je sais au fond de moi , le dr house l’a dit hier soir, que l’antidépresseur aide mais ne guérit pas… c’est un support, un soutien, pi comme bien des médicaments qui sont contre la douleur, contre l’arthrose il y a pas grand-chose à faire, donc c’est plutôt des soutiens qu’un médicament qui guérira.

**Patient 12**

A votre avis, qu’est-ce qu’un générique ?

Pour moi un générique est un médicament dont la molécule a été prouvée scientifiquement pour son efficacité et il y a tellement de temps alors par contre au niveau de la durée je sais pas trop mais après y a un moment au niveau des droits d’auteur ou des choses comme ça c’est tombé dans le domaine public en fait je sais qu’il y a une histoire de domaine public et donc ce qui fait que cette connaissance scientifique grosso modo si je simplifie à l’extrême appartient à tout le monde et que donc on peut utiliser après si on rentre dans…ça sort de la question. Donc voilà pour moi c’est ça un peu c’est une molécule guérisseuse qui est tombée dans le domaine public au niveau de ses droits d’auteur au niveau de..par rapport à sa création sa conception et c’est en tout cas prouvé scientifiquement pour son efficacité

(Donc on aurait une molécule de départ ?)

Alors cette molécule d’où elle vient alors à partir de là je me suis jamais vraiment trop posé la question mais pour moi théoriquement il y a deux possibilités, c’est que où cette molécule existe telle qu’elle dans la nature elle a été identifiée elle est reprise telle qu’elle dans un médicament, est-ce que il est susceptible qu’une molécule ait été créée en laboratoire soit pour imiter quelque chose qui existe dans la nature soit pour, est-ce qu’on peut prétendre créer en laboratoire une molécule nouvelle qui n’existerait pas dans la nature, mes connaissances scientifiques elles vont pas jusque là à priori je pense que tout existe dans la nature donc je pense pas qu’on puisse créer quelque chose de nouveau en laboratoire mais euh…mais voilà donc on reconstitue comme on parle des parfums de synthèse par exemple on reconstitue en laboratoire une molécule et puis il s’avère que cette molécule a des vertus particulières pour soigner tel ou tel mal

(Donc on aurait, quand on dit d’un médicament qu’il est générique c’est par opposition à médicament original, d’accord ?)

Euh…original ce serait quoi la définition d’original ? Ça serait si je reprends ma définition ça veut dire que ça serait une molécule qu’on viendrait seulement de parvenir à reconstituer en laboratoire et que enfin c’est très récent quoi donc y a eu y a une notion de nouveauté y a une notion de récent…qu’est ce qui pourrait y avoir d’autres comme notion…à priori je ne pense qu’à ces deux là

(Vous-même vous prenez des médicaments génériques ?)

Autant que possible par défaut c’est générique que je prends

(Par exemple ?)

Là par exemple dans ma médication on m’a prescrit du Tramal et dans la réalité je prends du tramadol…euh y avait quelque chose mais là je me souviens plus forcément je vais peut-être pas forcément être fiable au niveau des noms au niveau anti-inflammatoire je prends de l’Irfen mais l’original c’est…

(Brufen ?)

Brufen voilà le nom original c’est Brufen et le générique c’est Irfen donc voilà moi je prends l’Irfen…euh qu’est-ce-que j’ai d’autres ? euh épisodiquement du Sirdalud très honnêtement j’en sais rien si c’est un générique ou un original en tout cas j’sais qu’à la pharmacie moi je leur ai demandé un générique ils m’ont donné du Sirdalud mais dans la réalité je sais pas ce que c’est…euh du Dafalgan pareil je dois reconnaître à priori je dirais que c’est un générique mais j’en suis pas sûre à 100%...euh qu’est-ce-que je prends d’autres après c’est plus épisodique j’ai changé le médicament euh l’Omézol ah pareil l’Omézol ça je suis quasi sûre c’est un générique mais là je suis en train de changer maintenant je suis passé au Riopan mon médecin trouvait que c’était moins agressif euh…et le Riopan j’dois reconnaître que je sais pas j’ose espérer que c’est un générique mais j’dois reconnaître que je sais pas voilà à peu près les six médicaments que je prends

(D’accord. Pour vous, un générique ça a les mêmes effets secondaires pardon ça a les mêmes effets attendus et les mêmes effets secondaires que l’autre, que le médicament on va dire d’origine ?)

Ben en fait une chose que moi je n’arrive pas à comprendre et j’ai jamais pris le temps de faire des recherches là-dessus et malheureusement j’ai jamais trouvé d’articles suffisamment scientifiques pour être sérieux mais suffisamment vulgarisé pour que je puisse comprendre…euh pour savoir à partir du moment où il y a un médicament générique pourquoi on s’enquiquine à en faire un autre là y a quelque chose qui m’échappe. Je me dis si on en fait un autre ça veut dire que bon on retrouve la même molécule mais est-ce-qu’on y ajoute enfin est-ce-qu’il y a une différence et si y a une différence laquelle et ça je dois reconnaître que je ne sais pas mais j’ai jamais eu l’occasion de lire un article suffisamment scientifique et suffisamment vulgarisé donc je dois reconnaître que j’en sais rien. J’ai jamais compris dans la réalité pour moi en tant que néophyte c’est que l’original il est plus cher que le générique et je pense que je suis un citoyen qui est un petit peu soucieux des comptes tant de la confédération du canton de la ville et de mon portemonnaie également et je me dis sous entendu aussi indirectement des caisses maladies de dire tant qu’à faire autant prendre un médicament qui a prouvé son efficacité et qui revient moins cher à tout le monde quoi à la collectivité alors je peux comprendre que..j’ose espérer en tout cas que le médicament original la différence de prix ne va pas qu’augmenter les salaires des bénéficiaires mais c’est de l’argent qui va être reversé à la recherche au sein même du laboratoire qui va permettre de faire de la recherche pour d’autres médicaments j’ose espérer que ça fonctionne comme ça..là j’ai aucune preuve pour et j’ai aucune preuve contre donc j’en sais rien mais par défaut dans le doute je prône le générique parce que nos systèmes de société on va dire occidentales pour simplifier n’ont pas prévu leur efficacité en termes justement de rôle en termes de régulation et en termes de bon usage de l’argent donc que moi en tant que citoyen on me demande un effort pour participer financièrement à la recherche ça me paraît quand même cohérent mais dans ce cas-là il faudrait qu’il y ait un système de contrôle qui ait fait ses preuves pour montrer que cet argent ne tombe pas dans des scandales comme on a pu le montrer pour la recherche pour le cancer ou à un moment il y a des détournements de fonds et puis voilà qu’en y a qui disent il y a 15 millions qui ont disparu et c’est parti dans la poche de untel ou untel plutôt que de partir au bénéficiaire de la cause de l’association..euh tant qu’il y’aura pas de système un peu plus efficace ou tant que l’homme ne sera pas plus honnête qu’on puisse espérer qu’il soit dans le doute je me dis non il vaut mieux qu’il y’ait des investissements directs que des investissements indirects ça me semble peut-être plus efficace au moment au niveau des contrôles..donc je reviens à ce que je disais il y a deux minutes c’est que effectivement je n’ai jamais compris à partir du moment ou on a un médicament générique pourquoi on fabrique un original pour servir la même maladie…est-ce-qu’il y a des différences entre les deux en tout cas de ce qu’on m’a dit autour de moi de la part de médecins ou de pharmaciens à priori non donc de ce fait là je comprends pas il y a quelque chose qui m’échappe quoi et si j’essaie d’être deux secondes moins naïf ce qu’il y a c’est que je peux voir que le côté négatif quoi c’est de manipuler un petit peu le public et lui faire croire en disant mais celui-là c’est un vieux un petit peu désuet la maladie elle a un petit peu changé donc là on nous apporte un produit neuf quoi et celui-là il sera plus efficace que l’ancien alors qu’en fait c’est rigoureusement le même et les personnes qui seraient moins ou qui seraient plus fragiles peut-être émotionnellement pourraient se laisser peut-être je dis bien tout ça avec beaucoup de peut-être et beaucoup de guillemets se laisser avoir ou se laisser plus embobiner par euh ce genre de discours parce que c’est vrai que les discours publicitaires au jour d’aujourd’hui ils sont assez cruels ils usent et abusent de la naïveté des gens quoi

(Donc pour vous c’est la même chose, la même molécule ?)

Oui

(Vous me dites même molécule. Mêmes effets ?)

Oui

(Mêmes effets secondaires ?)

A priori oui

(D’accord)

(Est-ce-que à votre avis ce médicament, ces génériques sont aussi efficaces…)

Que l’original ? oui

(Est-ce-qu’ils sont soumis aux mêmes contrôles ?)

Oui parce que je pense là mes connaissances elles sont aussi un petit peu limitées mais les lois sont les mêmes pour tous donc que ce soit des lois fédérales ou internationales

(Donc si je vous demandais qui est-ce qui les fabriquent ces génériques à votre avis ?)

Qui les a fabriqué puisqu’effectivement ils sont très anciens alors…ça tombe dans le domaine public au bout de combien de temps, pour la musique c’est 70 ans…euh pour les livres euh…là j’avais fais la recherche parce qu’en plus ça m’intéressait mais ça me revient pas et on parle des médicaments pour les médicaments je dois reconnaître que j’en ai aucune idée…admettons que ça soit comme pour la musique 70 ans on fabrique un nouveau médicament en 2000-2010 donc ça veut dire qu’il a été créé dans les années 30 et 40 pour moi les années 30 et 40 je vais être très cliché mais je peux pas m’empêcher de penser à Curie à tous ces gens qui travaillaient en laboratoire un peu seuls avec des subventions à l’époque j’sais pas d’où elles venaient quoi mais elles pouvaient venir ou de laboratoire plus grand ou d’investisseurs privés ou de l’état ou je dois reconnaître que je sais pas trop en fait d’où ça venait…euh les méthodes de contrôle dans les années 1940 indéniablement n’étaient pas les mêmes que celles d’aujourd’hui j’imagine que c’est devenu de plus en plus sévère et de plus en plus rigoureux..j’ose imaginer aussi qu’à partir du moment on a mis des règles en matière de contrôle de plus en plus sévère on les a appliqué aussi aux médicaments qui étaient déjà en circulation j’ose espérer d’un point de vue hygiène sanitaire enfin hygiène publique ça me paraitrait cohérent… donc ces contrôles ont prouvé leur efficacité donc ils ont le même niveau de contrôle qu’un original qui sortirait au jour d’aujourd’hui je dois reconnaître que là par contre j’ai oublié votre question, euh votre question qui était où ils ont été fabriqué

(Qui est-ce-qui les fabriquent ?)

Qui est-ce-qui les fabriquent…en laboratoire moi j’dirais en laboratoire par des gens qui avaient suffisamment ou de connaissances ou de désir de plaisir à chercher de tester euh voilà qui avaient cette euh oui cette curiosité intellectuelle de se dire voilà ça y a un problème ça me passionne ou ça me soule qu’il y ait ce problème ou ça me passionne et je vais essayer de tout faire pour pouvoir apporter quelque chose une réponse

(Tout à fait. Alors en somme, visiblement vous cherchez toujours beaucoup d’informations ?)

Je suis quelqu’un assez curieux de nature

(Quand vous prenez un médicament ou quand le médecin vous prescrit un médicament, comment est-ce-que vous faites pour vous renseigner sur ce médicament, quelle type de source est-ce-que vous utilisez ?)

Ben bon déjà systématiquement je lis en intégralité la notice qu’il y a dans le paquet et je les gardent parce que quelque fois ça m’arrive comme là je l’ai refais y a pas longtemps pour le Sirdalud que j’avais arrêté depuis neuf dix mois ou je me suis dit tiens là j’ai tellement mal je sais que c’est un décontractant musculaire je veux aller me coucher est-ce-que ça peut m’aider à dormir c’est vrai qu’avant de le prendre comme il était onze heures du soir je pouvais pas téléphoner à mon médecin euh je l’ai d’abord relue pour voir parce que mon souci c’est de savoir éventuellement les incompatibilités avec les autres médicaments que je prenais donc ça été ça…euh si l’information me paraît suffisamment nette euh j’en reste là euh en règle générale de toute façon déjà y a une information qui est donnée par le médecin…euh à la pharmacie c’est assez rare je dois reconnaître qu’on me donne des informations euh si je la demande on me la donne si je la demande pas on me la donne pas forcément…euh on va simplement vérifier que je connais bien la posologie en fait….euh je suis en train de me poser la question est-ce-que j’ai déjà fait des recherches sur internet par exemple

(Oui, c’est la question que je vous avais posée)

Puis moi j’étais en train de me poser la question est-ce-que je l’ai fais ?....je pense pas l’avoir fait, je l’ai déjà fait pour des choses que par exemple moi je vais acheter sans ordonnance euh j’vais vous donner un exemple il n’y a pas tellement longtemps j’ai acheté de la pommade à la consoude en tant qu’anti-inflammatoire et c’est vrai que je suis allée faire une recherche sur consoude pour voir un petit peu ce que c’était…euh ça m’a confirmé effectivement que c’était un anti-inflammatoire etc et étant donné que dans la pommade qui était la pommade du Dr Andres qu’on m’a proposé j’ai essayé de voir si y’avait d’autres références avec moins de conservateurs ou moins de comment est-ce-que l’on dit ? des adjuvants ? enfin des additifs avec moins d’additifs quoi eh bon il s’avère que j’ai pas trouvé donc c’est vrai que de ce fait-là j’ai suivi le conseil qu’on m’avait donné j’ai demandé de nouveau confirmation à la pharmacie et puis donc j’ai acheté cette pommade dont j’ai effectivement été satisfait quoi..pour les autres médicaments non parce que c’est comme pour aussi des raisons de ma situation de santé actuellement j’ai fais très très peu de recherches sur internet j’en ai fais un petit peu quand même au début euh pour être sûre de bien comprendre les termes qu’on utilisait euh le terme tout bête comme hernie etc j’ai pas de formation de médecin même si j’ai fais un peu d’anatomie etc…je voulais être sûre de la définition je voulais être sûre mais le souci que j’ai c’est de trouver des sites internet fiables c’est ça un peu le problème et c’est vrai que ça s’rait bien que si on pense alors aux organisations les plus faitières du style OMS ou après au niveau fédéral euh spontanément il n’y a rien qui me vient à l’esprit mais qu’il y’ait peut-être comme ça un ou deux organismes réputés pour leur sérieux et réputés pour leurs connaissances scientifiques puissent effectivement créer un site internet qui serait irréprochable au niveau de l’information et de la connaissance tandis que là actuellement il y a un peu de tout et un peu

(A boire et à manger)

Voilà il y a à boire et à manger et donc j’ai tellement peur de tomber sur quelque chose qui soit pas fiable quand je vais sur des choses hyper pointues je vais très peu sur internet à l’aveuglette parce que j’ai peur de la fiabilité du site pour les médicaments encore plus et éventuellement pour les médicaments il faudrait quoi il faudrait deux types de site il faudrait 1) le site du laboratoire mais en même temps forcément le laboratoire il sera subjectif mais ça peut être intéressant déjà de voir un petit peu le laboratoire voire un peu l’histoire le pourquoi comment etc…ça dans l’absolu c’est susceptible de m’intéresser et puis après de voir si éventuellement par rapport aux étapes de contrôle que je reconnais ne pas connaître très très bien de voir un peu l’organisme de contrôle qui a étudié ce médicament de voir un petit peu lui sa version des faits au niveau des vertues et au niveau des effets secondaires et puis après pourquoi pas un autre site de de..on va dire peut-être plus de témoignage au niveau effectivement des effets secondaires au niveau de l’association de médicaments au niveau peut-être un côté un petit plus pragmatique pour moi l’idéal ce serait un petit peu ça

(Quand vous dites témoignage ce serait d’autres patients ?)

Oui éventuellement d’autres patients ou d’autres médecins oui ça peut aussi bien être des patients que des médecins des praticiens parce qu’un médecin peut-être que ça existe dans votre corporation vous en interne parce que je sais qu’il y a des journaux qui des publications etc ou éventuellement un médecin pourrait dire comme d’habitude « j’ai prescris ce médicament pour telle pathologie et puis pour la première fois depuis dix ans que je prescris ce médicament j’ai eu tel effet secondaire » alors la question se pose se dire ah oui tiens la particularité de ce patient c’est qu’il avait tel autre médicament peut-être que ça m’étais jamais arrivé avant ou est-ce-que la situation physiologique pathologique de ce patient était un petit différent des autres ce que je vois par rapport à ma situation de dos c’est hallucinant quand j’en parle autour de moi c’est hallucinant le nombre de personnes si tu veux je peux te donner les coordonnées d’un physio qui est génial ou les coordonnées d’un médecin qui est génial etc mais je pourrais réécrire un bottin avec toutes les coordonnées qu’on m’a donné et puis c’est génial pour quelqu’un et c’est pas génial pour moi parce qu’effectivement la pathologie n’est pas rigoureusement la même et cela au mois d’août de cette année donc dans trois à quatre mois cela va faire deux ans que j’ai ces soucis même si je vais nettement vers le mieux et y a certains aspects de ma pathologie que je commence seulement à comprendre maintenant c’est assez dingue quoi par rapport à ce que j’ai pu lire ou les questions que j’ai posé et les réponses qu’on m’a donné par rapport à mes hernies…euh par rapport aux hémangiomes ou par rapport à ces trucs-là j’ai l’impression d’arriver avec un peu plus de précisions et un plus de détails sur je vais dire le mot diagnostic qui est un mot trop professionnel pour que je puisse l’utiliser à titre personnel mais il y a un petit peu de ça quand même quoi… faut dire que c’est vrai qu’il y a des choses au niveau des symptômes que je pourrais donner avec plus de précisions maintenant que je n’ai pas pu donner il y a dix-huit mois en arrière parce qu’il y avait certains endroits où la douleur était tellement forte qu’elle cachait en fait un certain nombre de symptômes maintenant cette douleur hyper aiguë elle a énormément diminué ce qui fait qu’il y a d’autres endroits d’autres zones qui s’expriment et que seulement maintenant j’entends en fait

(D’accord ce qui veut donc dire que les informations que vous pourriez vous donner ou les informations que vous pourriez souhaiter avoir pourraient aussi évoluer dans le temps ?)

Oui indéniablement oui

(Alors si je vous demandais par rapport aux génériques, qu’est-ce-que vous aimeriez avoir comme information ?)

Moi je me sens un peu satisfait par rapport aux informations qu’on a sur les génériques tout à l’heure au tout début de l’entretien quand on commençait l’enregistrement j’me disais à mon avis y a un problème de communication à la base c’est que moi je suis sidéré quand j’ai vu la communication..elle a commencé quand en Suisse..j’ai commencé à l’avoir il ya deux j’sais pas c’est assez récent j’dirais trois ans en arrière où on commençait à parler des génériques à la télévision un petit peu comme pour démystifier en disant bon écoutez on a quelque chose un peu, on a un peu peur de vous dire quelque chose mais vous savez il est pas méchant celui-là effectivement il est un petit peu vieillot mais tout aussi efficace..j’avais l’impression que le message publicitaire essayait de se déculpabiliser ou euh il y avait un côté un petit peu timide moi j’aurais vu plus un message que j’ai trouvé très pertinent en France là c’est beaucoup plus vieux peut-être une petite dizaines d’années en arrière ou le message c’était par rapport à ces quoi ces médicaments quand on dit qu’on est sous (antibiotiques ?) sous antibiotiques « les antibiotiques c’est pas automatique » j’ai trouvé ça génial comme message parce que je trouvait que c’était un message simple et puis on comment dire ça ? y avait une très très grande sérénité dans le message tandis que là dans le message qui est passé depuis trois ans sur les génériques pour moi y a pas de sérénité qui est passée (De sérénité, vous voulez dire ?) ben de sérénité c’est que pour moi ce serait de simplifier à l’extrême de dire voilà les antibiotiques c’est pas automatique les génériques c’est automatique voyez de dire mais attendez vous consommateurs…vous avez vraiment trop d’argent…enfin de trouver une autre source je suis pas publiciste publicitaire non plus mais de dire c’est une question de revenir à une notion de bon sens en fait en plus on est en période de crise actuellement où les gens font attention à leur portemonnaie et se dire vous voulez faire des économies ben déjà commencé par des génériques…générique c’est automatique voyez ?

(D’accord, donc ce serait l’autre message ?)

Oui ce serait l’autre message, c’est de un message simple tandis que là on commence à dire oui mais bon vous savez alors effectivement y a ça mais ça s’rait cool quand même si vous pensiez aux génériques et il y a comme un doute pour moi c’est comme ça que je le ressens c’est un débat que je n’ai jamais eu avec mes amis mais grâce à cet entretien je pense que la prochaine fois que je vais voir des potes je vais en parler avec eux pour voir un peu comment eux le ressentent et moi je sentais un peu comme de la culpabilité un peu dans le message publicitaire j’ai senti que on n’assumait pas le propos puis on se disait ouais on avait un peu peur de déranger quoi alors qu’il aurait fallu un message mais très naturel très simple

(Alors une patiente m’a dit, je ne sais pas si ça rejoint un peu ce que vous dites là, elle m’a dit « j’ai un peu l’impression qu’on essaie de les faire passer en catimini »)

Y a un message qui n’est pas assumé voilà tout à fait

(C’est un peu…)

Y a un peu de ça ouais alors en catimini c’est pas rigoureusement ça l’idée je la trouve assez intéressante effectivement c’est que moi je le sens pas parce que en catimini c’est que on essaie de le faire le plus discrètement possible puis on n’assume pas complètement tandis que moi en plus du catimini j’dirais que j’ajoute un sentiment de culpabilité de la part du promoteur de ce message en fait et je l’ai jamais comprise en fait cette culpabilité jamais

(Alors vous votre médecin, il vous a déjà proposé si je comprends bien)

Le médecin jamais

(Le médecin ne vous en a jamais proposé ?)

Non non c’est ça qui est drôle il me dit vous prendrez du Tramal vous prendrez du Brufen etc donc moi j’y connais rien sur les noms donc euh ok puis après je vais à la pharmacie en disant vous me mettez svp que des génériques puis c’est là que j’apprends que le Tramal s’appelle tramadol que le Brufen s’appelle Irfen et puis c’est vrai qu’après le rdv suivant moi j’ai déjà oublié les noms des médicaments originaux donc vous prenez quoi actuellement puis ben spontanément je reprends les mots que moi j’utilise alors au niveau du tramadol ben j’sais j’en suis là là là puis ah oui le Tramal et à c’est à ce moment-là que je me rappelle en fait parce qu’entretemps j’oublie quoi j’attache pas trop d’importance mais moi j’ai pu voir sur les deux, j’ai eu deux médecins généralistes ces 18 derniers mois, et à chaque fois c’est euh quoique peut-être plus celui que j’ai actuellement la Dre que j’avais avant euh…j’ai un souvenir mais alors là il faudrait que j’aille creuser dans ma mémoire parce que j’ai vaguement le souvenir qu’un moment par rapport on parlait de médicaments elle a sorti son gros bottin des médicaments et est-ce-qu’elle m’a dit alors qu’est-ce-qui existe en générique puis d’aller vérifier de voir le médicament elle avait bien le titre original dans sa tête mais par rapport au générique elle avait un doute elle est allée vérifier mais est-ce-que c’était bien ça parce que de temps en temps elle allait aussi vérifier si c’était par rapport aux tailles des boîtes mais grosso modo enfin dans..les médecins ont plutôt tendance à me proposer des médicaments originaux que des génériques quoi

(Est-ce-que c’est quelque chose dont vous avez parlé avec le médecin ?)

Non jamais non parce que je me dis que j’ai pas envie de rentrer dans ce débat avec lui euh il a son point de vue ça le regarde euh à la fin c’est moi qui aurais le dernier mot de toute façon puisque c’est moi qui vais les chercher à la pharmacie donc voilà donc euh je sais que j’aurais le dernier mot

(Donc ce n’est pas la pharmacie qui vous propose le générique, c’est vous qui demandez le générique ?)

Alors il est arrivé une ou deux fois que j’oublie à la pharmacie de demander des génériques je donnais l’ordonnance et euh elle partait dans ses tiroirs et puis du tiroir me dit euh mais je vous donne celui-là ou je vous donne un générique puis je dis ah merci de le demander oui je prendrais un générique svp donc à la pharmacie très souvent si moi j’oublie de demander et qu’elle a un doute ou même dans son ordinateur parce que je suis fidèle je vais toujours à la même pharmacie dont maintenant tout est listé et puis c’est déjà arrivé de dire ah oui vous vous prenez le générique et euh mais à la pharmacie oui ils sont plus attentifs à ça en tout cas

(Ca vous semble une réaction appropriée de proposer le générique ?)

Oui oui

(A la pharmacie comment réagissent-ils quand vous demandez le générique ? vous avez l’impression qu’ils pensent eux aussi que c’est approprié que vous posiez la question, vous avez l’impression que ça les surprend ?)

Surprend non

(Qu’ils sont habitués ?)

Oui oui non je sens que ça ne choque pas ça ne les surprend pas mais j’ai pas souvenir d’avoir perçu une émotion quelconque d’approbation ou de désapprobation par rapport à ça…j’ai pas non j’ai pas souvenir mais eux en tout cas ils m’ont posé la question assez naturellement

(Donc ça ne vous est jamais arrivé d’hésiter à prendre un médicament parce que c’était un générique ?)

Non jamais

(D’accord, ça vous est arrivé d’hésiter à prendre un autre médicament, je vais dire à prendre un médicament je vais le dire comme ça, ma question est très mal formulée. Vous n’avez jamais hésité à prendre un médicament parce que c’était un générique, est-ce-qu’il vous est arrivé d’hésiter de prendre un médicament ?)

………euh quelque soit le fait qu’il soit générique ou pas générique ? euh alors là spontanément j’ai pas d’exemple qui me vient à l’esprit………alors voyez par exemple y a un médicament que très très ponctuellement et dieu merci que j’utilise de moins en moins mais que je n’aime pas prendre comment est-ce-qu’il s’appelle c’est celui pour l’asthme (Ventolin ?) le Ventolin c’est un médicament que j’aime pas prendre mais en même temps que je prends en cas de crises d’asthme parce que je dois reconnaître son efficacité euh c’est un médicament que j’aime pas par rapport à ses effets secondaires euh…mais voilà c’est le seul qu’on me propose donc euh

(Mais s’il était un médicament, s’il y avait un générique ça changerait quelque chose ?)

Ah non non

(On est d’accord, c’est parce que c’est le médicament lui –même ?)

Oui c’est le médicament lui-même alors c’est vrai que par rapport à celui-là je dois reconnaître que je sais pas si c’est un générique ou pas

(Ok mais je peux pas vous le garantir)

(Donc est-ce-que, de manière générale, votre pharmacien quand vous lui demandez le générique ou quand éventuellement vous n’y avez pas pensé et qu’il vous le propose, est-ce-qu’il vous donne une information sur le fait que c’est un générique ou pas ?)

Ben à partir du moment où c’est moi qui lui demande peut-être que j’ai l’impression de lui donner le message je n’ai pas besoin d’informations donc non il ne m’en donne pas puis je ne lui en demande pas non plus

(D’accord. Donc en somme si je comprends bien, pour vos médicaments contre vos douleurs les génériques c’est vous qui les avez demandé et c’est pas, il n’y a jamais eu de proposition de votre médecin pour un générique et c’est toujours vous qui les avez demandé, j’ai bien compris ?)

Alors je dirais… (jamais ?) complètement voilà c’est ca (on va dire de manière générale) la plupart du temps j’ai jamais parce que je fais pas attention

(De manière générale c’est plutôt votre proposition, c’est votre idée de toujours demander le générique plutôt qu’on vous le propose)

Voilà c’est ça j’ai jamais vérifié quand le médecin quand il écrit l’ordonnance lui demander si c’est un générique parce que comme je sais qu’à la pharmacie il n’y aura pas de problème…si la pharmacie causait un problème attendez sur votre ordonnance c’est ça qui est écrit alors là je serais beaucoup plus vigilant en amont dans ce cas-là je dirais au médecin ok mais vous me mettez des génériques parce qu’à la pharmacie sinon ils vont me faire des problèmes parce qu’il y’a substitution de médicaments mais comme la pharmacie ils font pas de problèmes moi je laisse le médecin faire son métier et puis après à la pharmacie je dirais maintenant vous me prenez la traduction simultanée et puis voilà

(D’accord. Je suis assez attentive à la manière dont les gens parlent de ces génériques, vous vous avez utilisé le mot traduction simultanée, y a des gens qui ont dit copie ou pâle copie. Qu’est-ce-que vous pensez de ça ?)

Ben je trouve que c’est erroné pâle copie non c’est copie identique mais ça rejoint ce qu’on disait effectivement tout à l’heure quelle est rigoureusement la différence entre les deux moi à priori je dirais aucune maintenant s’il y en a une j’aimerai bien savoir laquelle mais je ne sais pas

(D’accord, ok)

Tant qu’on m’a pas prouvé qu’il y a une différence par défaut je dis qu’il n’y en a pas

(Alors c’est intéressant parce que vous avez le point de vue, on pourrait entendre le point de vue tout à fait inverse, « tant qu’on ne m’a pas prouvé l’identité j’attends de voir »)

Non cette remarque là serait absolument pertinente quoi mais en même temps on commence malgré tout même si j’ai pas aimé cette pub euh le message qu’on fait passer c’est que il n’y a pas de différence donc alors ces personnages je leur dis qu’est-ce-qu’ils vous faut de plus comme preuves pour qu’on vous prouve que ça a pas de différence sinon c’est une publicité mensongère c’est attaquable donc ces personnes-là je pense que ça m’est déjà arrivé à la pharmacie d’entendre effectivement un patient à côté qui disait non non moi c’est celui-là que je veux alors que le pharmacien lui proposait un générique et il a refusé y’a pas eu débat entre les deux le pharmacien ou la pharmacienne n’a pas cherché à négocier ou chercher à comprendre ben voilà peut-être parce qu’elle est échaudée et que chat échaudé ben voilà vous savez elle s’est dit moi ça fait vingt ans que je bosse des patients comme monsieur au début oui dans ma jeune carrière j’ai tenté et puis maintenant je laisse tomber parce que je suis épuisée ça m’a fait perdre des heures et des semaines de boulot inutile à essayer de convaincre un patient et puis peut-être que ça n’est pas mon boulot que c’est le rôle du médecin de prescrire directement le générique peut-être sans même le dire au patient sauf si le patient lui demande voilà est-ce-qu’il y a un médicament un peu plus récent mais c’est peut-être le boulot dans ce cas-là du médecin quoi de prescrire directement le générique

(Qu’est-ce-que vous en pensez vous ?)

Je pense que ça pourrait être pas mal parce que de ce fait parce que si le médecin ne connait pas son patient euh de cet aspect-là en tout cas le doute du patient euh c’est vrai que ça peut perturber des patients de dire attendez là c’est mon médecin il m’a dit qu’il fallait que je prenne ça mais là vous êtes en train de tout changer vous êtes pas médecin quoi vous êtes pas mon médecin donc ouais mais il faudrait voilà une personne qui serait un petit peu dans cette position de vraiment de mal à l’aise en disant mais attendez moi j’fais 100% confiance à mon médecin s’il a prescrit ça c’est parce qu’il veut absolument que je prenne ça là vous me changez le truc non non vous m’avez pas ausculté vous êtes pas mon médecin je vous fais moins confiance vous me mettez ce qu’il y a sur l’ordonnance alors peut-être ça pourrait répondre à X% de patients ça serait intéressant effectivement de voir si tous les médecins prescrivaient systématiquement des génériques euh combien de patients une fois arrivé en pharmacie diraient bon écoutez là il m’a prescrit de l’Irfen mais vous me mettez du Brufen à la place ça serait effectivement intéressant de voir si y a des patients qui réagiraient comme ça tandis que là si d’emblée on dit ben c’est de l’Irfen qui est prescrit donc je vous donne de l’Irfen….le stress a pas lieu d’être tandis qu’on lui dit Brufen non non mais je vais vous mettre de l’Irfen parce que c’est un générique ouais mais alors effectivement si c’est dans cette logique mais c’est une pâle copie c’est un truc un peu vieillot recette de grand-mère pas très fiable y a plein de poussières dessus non non moi je veux le truc neuf qui sort du labo peut-être que certains patients réagissent comme ça je ne les connais pas suffisamment ces patients-là mais donc euh oui ça pourrait être intéressant d’obliger les médecins à prescrire directement des génériques parce que les médecins en même temps sont démarchés par des visiteurs médicaux ou je sais plus comment on les appelle qui viennent faire leur publicité….est-ce-que c’est normal qu’au niveau de la santé il y’ait de telles démarches…ça mériterait débat je trouve parce qu’effectivement ça beau être un médicament ça rentre dans notre société un peu capitaliste de démarche commerciale mais là on tient à la santé de patients en plus les caisses seraient hautement excédentaires peut-être qu’on pourrait se permettre mais là maintenant de toute façon à la pharmacie ils m’ont dit que les caisses ne remboursaient qu’à hauteur du générique et tout ce qui étaient frais supplémentaires au générique de toute façon c’était à la charge du patient mais de nouveau si le patient après faut voir quelle est sa démarche intellectuelle ou émotionnelle euh du pourquoi il est prêt à payer 20 ou 25% plus cher si c’est uniquement parce qu’il s’est fait manipuler je trouve ça un peu dommage quand même et euh oui je trouve ça un petit peu dommage donc……..la démarche du visiteur médical c’est déjà arrivé à une époque ou financièrement j’étais vraiment juste juste juste c’est arrivé que mon médecin me donne des échantillons qu’il avait reçu par exemple et…alors moi j’était content parce qu’effectivement ça me faisait faire une sacrée économie et puis après je me suis dit mais c’est pas je sais pas si c’est vraiment sain en fait parce que les visiteurs ça veut dire laissent des échantillons ces échantillons après les médecins en font un petit peu ce qu’ils veulent euh voilà qu’est-ce-qu’ils en font exactement là on a vu un des côtés éventuellement positifs de dire ben tiens on va donner un petit coup de pouce à tel ou tel patient parce qu’actuellement financièrement c’est un peu just donc ben autant qu’ils en profitent est-ce-qu’il peut y avoir d’autres euh je sais pas détournement abus excès ça veut dire que les gens qui travaillent dans cet endroit-là peuvent s’automédiquer avec les échantillons qu’ils ont reçus ça leur fait faire des économies à eux aussi pourquoi pas ça peut-être un des avantages de la profession s’ils ont ce petit avantage là bon je suis pas contre l’idée ça m’est un peu égal à vrai dire mais il peut y avoir aussi un intérêt quoi c’est ça qui me plait pas quoi

(Donc en somme vous, si je devais résumer, vous dites que vous êtes prêt à militer pour les génériques en somme c'est-à-dire ce que vous appelez une traduction simultanée en termes de molécule, en termes d’effets, en termes d’effets secondaires et qui en plus se fait à moindres coûts, c’est bien ça ?)

Oui

(Et votre expérience à vous c’est globalement une expérience très positive, pour autant bien entendu que le médecin soit efficace mais pas de différence dans les expériences que vous avez pu faire quelque soit les médicaments antalgiques ou non pour lesquels vous avez eu des originaux ou des génériques, c’est juste ?)

Oui tout à fait

(J’arrive au bout des questions que je voulais vous poser, est-ce-que vous voulez ajouter encore quelque chose par rapport à cette thématique des génériques ?)

Ben y a juste une expression qui vient de me revenir alors ça va être redondant avec ce que j’ai déjà dis c’est par rapport quelle est la différence de l’original par rapport aux génériques donc vous connaissez mon point de vue et ma réponse là-dessus et parce que j’ai un petit peu l’impression que l’original c’est un peu comme inventer l’eau chaude quoi ou réinventer l’eau chaude ou réinventer le fil à couper le beurre on utilise l’expression qu’on veut et c’est vrai qu’il y a une sorte de dynamique comme ça un petit peu que j’ai toujours pas comprise quoi donc euh c’est la seule chose je vous dis c’était par rapport à une histoire de placer ces deux expressions-là mais complètement redondant avec ce que j’ai déjà dis

(D’accord, on va dire que ça illustre votre propos tout comme l’expression de traduction simultanée qui me semble tout à fait intéressante à relever)

Fin de l’enregistrement

**Patient 13**

(A votre avis, qu’est-ce qu’un générique ?)

C’est un médicament qui a la même fonction pratiquement qu’un médicament connu mais qui est moins cher, alors je ne comprends pas pourquoi on laisse les médicaments chers sur le marché juste parce qu’ils sont connus

(Qu’est ce que vous entendez par pratiquement la même fonction ?)

En discutant avec mon mari qui a eu un générique pour des problèmes de thyroïde ça n’a pas eu exactement le même effet, et j’ai un ami qui a les cancer lui non plus ça n’a pas été exactement la même chose. Ça dépend des médicaments, parfois ils remplacent très bien, d’autre il faudrait voir les composants

(Qu’est ce que vous entendez par médicament connu?)

Connus par ce que le nom du médicament est plus répandu et les gens le connaît par exemple avant il y avait le Ponstan et maintenant c’est le méfénamide mais les gens connaissent mieux le Ponstan

(Vous pensez que dans le Ponstan et méfénamide il y a la même substance ?)

Je pense que oui, cela fait longtemps que je ne le prend pas, je pense que oui ça devrait être la même chose

(Pour vous, il y a eu une différence entre le Ponstan et méfénamide?)

Pour moi oui je n’ai pas vu de différence. J’ai aussi eu la fluocime et la fluoxétine et j’ai pas fait de différence sincèrement

(Pensez-vous que les génériques ont plus d’effets indésirables ?)

Je ne crois pas, je pense pas, pour moi c’est identique

(pensez-vous que les génériques sont soumis aux mêmes contrôles ?)

Je pense que oui du moment qu’ils sont sur le marché, vous nous aviez dit que les tests durent longtemps, je pense que oui

(Quelles sont les différences ?)

C’est-à-dire que moi je réagis mieux au Ponstan qu’au Méfénacide, Méfénacide ça ne me fait strictement rien tandis qu’un Ponstan ça m’aide

(Est-ce-que vous pensez qu’il est plus facile ou plus difficile d’avaler un générique que le médicament princeps ?)

Non

(Vous savez qui les fabrique ? Les génériques)

Non…les mêmes peut-être, mais je ne vois pas le but…c’est un peu compliqué tout ça, si c’est les mêmes qui font les médicaments connus qui au bout d’un temps d’années peuvent être copiés alors pourquoi la même entretprise ferait les deux, c’est pas logique

(Est-ce-que vous hésiteriez à prendre un générique ?)

Non si on m’assure que l’effet est le même, c’est injuste de payer plus cher, pourquoi si c’est la même chose

(Qui est « on » ?)

Le médecin et le pharmacien, en principe les pharmaciens sont quand même des médecins, s’ils les proposent je fais confiance

(Comment vous avez entendu parler des génériques ?)

Par le pharmacien, le médecin pas spécialement, le médecin prescrit et c’est le pharmacien qui propose un générique il me dit est-ce que cela vous gène de prendre un générique ?

(Media ?)

Non pas spécialement, plutôt le pharmacien

(Est-ce-que vous avez été chercher des informations sur les génériques sur internet par exemple ?)

Non

(Et puis est-ce-que vous allez parfois sur internet pour une info sur la santé ?)

Oui sur la fibromyalgie j’ai vu beaucoup de chose, ça m’a éclaircie et j’ai trouvé des livres que j’ai été acheté et que j’ai lu

(Sur des forum ?)

J’ai fais mais ça m’a pas apporté grand chose

(Sur des médicaments ?)

Non pas des médicaments, en principe il y a la notice

(votre médecin vous a déjà prescrit des génériques ?)

Non

(Votre pharmacien vous a déjà proposé de substituer le médicament original ?)

En principe il dit est-ce que je peux vous donner un générique, c’est la même chose amsi moins cher.

(Vous lui posez des questions ?)

Pas tellement de temps de demander et qu’il nous explique, il y a trop de monde et je ne suis pas sûre qu’ils savent les informations pour répondre

(Pourquoi ?)

parce que ça vient de plus haut, on leur dit de le faire

(en haut c’est qui ?)

Des chefs, des réunions entre collègues

(Quelle est votre expérience personnelle ?)

Avec le Ponstan et le méfénamide j’ai pas vu de différence sur l’effet et les effets indésirables

(Avec le Tramal ?)

Au début j’avais pris le Tramal en gtt de mon mari je ne sais pas si c’est le produit ou si j’en ai pris trop mais j’ai eu des hallucinations et j’ai arrêté. Après le médecin m’a prescrit des comprimés mais tout de suite des génériques et je les ai supportés

(Avez-vous un commentaire ?)

Si le médecin prescrit une chose et que le pharmacien donne une autre chose alors c’est la confusion, le doute pour certains ,c’est comme lorsqu’on est à la Migros il y a un produit et à côté le produit M budget alors on se demande la différence pourquoi un est moins cher. Il faudrait que tous les médecins prescrivent les génériques.

**Patient 14**

(A votre avis, qu’est-ce qu’un générique ?)

Comment dire, c’est une copie d’un médicament, d’un médicament breveté, c’est une réplique sous un autre nom.

(Est-ce la même substance dans les deux ?) c’est la même substance il n’y a pas de différence

(même efficacité ?) oui tout à fait

(même effets indésirables ?) oui, oui c’est la même substance

(Est-ce-qu’ils sont soumis aux mêmes contrôle que l’original?) aux même contrôles oui…mais je ne sais pas….je fais confiance à mon pays

(Plus difficile à avaler ?) non

(Qui est-ce qui les fabriquent ces génériques à votre avis ?) ….une firme concurrente ou la même je ne sais pas….ça fait un moment qu’on en parle plus des génériques

(Qu’est-ce qui vous ferait hésiter de prendre un générique ?)

Non j’hésiterai pas pourquoi…ça ne me gêne pas car j’en ai parlé avant avec mon médecin, mais s’il ne me dit rien alors j’en prends pas. (Si votre médecin vous en parle pas ou vous en prescrit pas alors vous hésiteriez ?) oui je fais confiance qu’au médecin, il décide

(avez-vous entendu parlé des génériques ?)

Par la presse écrite, surtout « le Temps » (Qu’avez-vous retenu ?) …..des histoires de gros sous, mercantiles, intimement liées avec les compagnies d’assurance….un vrai casse-tête financier. (Souvenez-vous des campagnes d’affichage en ville ?) non pas du tout

(Internet ?)

Non ni sur les génériques, médicaments ou santé

(Votre médecin parlé ou proposé ?)

Il m’en a parlé un peu mais pas prescrit. (Que vous a-t-il dit ? ) en fait c’est plutôt moi qui ai posé la question, c’est plutôt moi qui parle de ce problème, mais il m’en a jamais prescrit.

(et votre pharmacien?)

Il m’en parle systématiquement, mais je n’ai pas pris car c’est d’abord mon médecin qui décide (vous refusez alors la substitution proposée par le pharmacien ?) oui je refuse, je ne prends pas d’initiative personnelle

(Savez-vous qu’il existe un générique pour votre traitement (Tramal et Dafalgan)?) Non, mais j’imagine que oui car le pharmacien me l’a proposé pour le Dafalgan, mais j’ai pas aimé ça c’est le médecin qui est responsable par rapport au choix du médicament. Je change que si le médecin le décide.

(Avez-vous une expérience avec un générique ?)

Oui avec un anti-inflammatoire mais je me souviens plus du nom (Médecin vous l’avait prescrit ?) oui il avait mis le nom et m’avais dit de prendre le générique à la pharmacie.

(Comme cela vous le prenez un générique ?) Bon moi je suis pour les marques et pas les copies, je préfère acheter un vrai Vuitton qu’une copie, mais là le médecin, oui ça a suffit pour me rassurer

(Effets ?) plutôt bien, enfin chez moi ces médicaments ne marchent pas, ils font rien sur les douleurs

(Effets par rapport aux autres de marque?) même effets j’ai rien noté de particulier, de négatif

**Patient 15**

(A votre avis, qu’est-ce qu’un générique ?)

Bon il y a les médicaments officiels et puis les génériques qui sont pas tout à fait pareils. (on peut dire que c’est une copie ?) non pas tout à fait une copie, une copie partielle parce qu’il y a des autres substances. (pourquoi pensez-vous que des génériques sont mis sur le marché ?) pour faire des économies, ils sont moins chers et puis des firmes étrangères peuvent alors les vendre en Suisse (vous pensez que les firmes étrangères ne peuvent pas vendre des médicaments ‘officiels’ en Suisse ?) non c’est que des médicaments suisses

(Est-ce la même substance dans les deux ?) oui c’est la même substance mais avec d’autres

(même efficacité ?) oui

(même effets indésirables ?) non les autres substances peuvent faire des effets indésirables en plus (comme quoi ?) des allergies

(Est-ce-qu’ils sont soumis aux mêmes contrôle que l’original?) je ne sais pas oui je l’espère

(Plus difficile à avaler ?) non

(Qui est-ce qui les fabriquent ces génériques à votre avis ?) je ne sais pas des firmes pharmaceutiques mais pas les mêmes que les médicaments officiels

(Qu’est-ce qui vous ferait hésiter de prendre un générique ?)

Non j’hésiterais pas si mon médecin me dit de le faire. (Si votre médecin vous en parle pas ou vous en prescrit pas alors vous hésiteriez ?) euh non je fais confiance à mon pharmacien que je connais depuis longtemps

(Avez-vous entendu parler des génériques ?)

Par les gens autour de moi, à la pharmacie, à la TV (Souvenez-vous des campagnes d’affichage en ville ?) euh oui

(Internet ?)

Non pas sur les génériques, mais il m’arrive d’aller voir pour des mots que je ne comprends pas (comment faites-vous) je tape sur google le mot et je regarde, mais je n’ai pas toujours la réponse, hier je suis aller voir sur un problème de l’os qui est décrit dans la radio que je viens de faire, mais il parlait de ça dans le dos mais pas dans le genou alors je n’ai toujours pas la réponse

(Votre médecin vous a parlé ou proposé des génériques?)

Non je ne sais pas si les médicaments que je prends il y a des génériques (que prenez-vous actuellement ?) du MST, du Celebrex, du Tryptizol et du Dafalgan (le Tryptizol et le Dafalgan sont des génériques) ah je ne savais pas

(Et votre Pharmacien?)

Non

(Avez-vous une expérience avec un générique ?)

Je ne savais pas que j’en prenais, mais je n’ai rien contre, je suis d’accord de faire des économies. (avant il vous est arrivé de prendre du Brufen ?) oui (pour ce médicament il existe de nombreux génériques, en avez-vous pris ?) je ne sais plus

**Patient 16**

(A votre avis, qu’est-ce qu’un générique ?)

Bah un générique d’après ce que j’ai pu comprendre c’est un médicament qui a les mêmes bases que le médicament normal entre guillemets mais qui est meilleur marché. Voilà pour moi vulgarisation du mot générique.

(d’accord alors quand vous dites qui a les mêmes bases qu’est ce que vous entendez par là ?)

C’est à dire qui a les mêmes possibilités de guérison, il a les mêmes molécules d’après ce qu’ils expliquent, qu’il y a rien de différent du médicament réel mis sur le marché par les laboratoires pharmaceutiques.

(d’accord donc ce serait le même médicament ?)

probablement oui d’après ce que je comprends

(est ce qu’il y aurait des différences ?)

on m’en a jamais parlé

je lis souvent donc le mode d’emploi j’ai pas vu de différences au contraire ils disent que c’est identique au médicament père si vous voulez mais qui est fait de façon qui revient meilleur marché

(et à votre avis pourquoi est ce que ça revient meilleur marché ?)

bonne question. Est ce que les matières première ils arrivent à les obtenir meilleur marché. Est ce que ils ont moins d’employés. Est ce que euh… honnêtement je sais pas pourquoi il est meilleur marché c’est une bonne question.

(est ce que ca aurait des conséquences le fait qu’il soit meilleur marché ?)

je crois pas. Sur le porte monnaie oui. Mais est ce que…. Je sais qu’il y a des gens qui croient aux génériques et d’autres qui y croient pas. Moi mon médecin docteur B quand il y a la possibilité il me donne un générique, les antibiotiques par exemple il donne un générique et je pense pas que le générique soit mauvais puisque le résultat était excellent après.

(donc vous avez eu un bon résultat vous avec les génériques)

avec les génériques oui. Il y en a eu ou c’était pas terrible par exemple le comment il s’appelle ponctium ?quand on a une hernie hiatale ? pour les remontées d’acide (le Nexium ?) Nexium qu’on appelle la rolls dans ce domaine me fait beaucoup plus d’effets que le générique que j’avais avant je me souviens plus son nom je sais pas si c’était zierca ou quelque chose d’autre mais je sais qu’il y en a un c’était un générique mais ça m’a pas convenu mais depuis que je prends le Nexium 40 ca va nettement mieux

(à votre avis elle vient de quoi alors la différence ?)

j’aurais du poser la question à mon beau frère qui est ( ?????) avant de venir

(c’est votre avis qui m’intéresse)

est ce qu’ils arrivent à obtenir la même composition que l’original mais meilleur marché…c’est ça à mon avis mais comment ils le font je l’ignore je pense que la base d’un médicament la composition d’un médicament si c’est les mêmes molécules qui sont dans l’un ou dans l’autre doivent couter au départ le même prix alors qu’elle est la différence qu’ils arrivent à le vendre nettement meilleur marché très franchement je l’ignore

mais j’ai jamais creusé la…comme je fais confiance au médecin j’ai pas été plus loin là-dedans

je prends un autre cas j’ai utilisé du Flector pour mes douleurs euh le flector a un générique bon j’ai pris une fois le générique je l’utilise plus, il colle moins bien et puis le je sais pas il j’ai pas l’impression qu’il me donne à 100 % satisfaction alors vous voyez il y en a qui sont pas terribles, il y en a d’autres par exemple les mepha qui sont bons et la pharmacie me demande souvent quand je vais est ce que vous voulez des génériques ? parce que je sais que les assurances les poussent pour les génériques alors est ce que ils les poussent par économie à mauvais escient ou est ce que ils les poussent par ce que c’est aussi bien ca c’est une question que moi je vous poserai et à laquelle vous pourriez me répondre

(à votre avis)

à mon avis si on a inventé un médicament qui donne satisfaction je crois pas qu’une imitation soit aussi bonne. A mon avis je suis pas un scientifique vous savez je suis un enquêteur et pour moi dans mon métier vous voyez on relevait l’ADN comme on relevait les empreintes mais c’est clair que je sais pas honnêtement je ne sais pas peut être que le générique n’est pas aussi bon que le vrai, l’original

mais alors je pose la question pourquoi les grandes sociétés comme XX et autres qui gagnent des milliards y a qu’à voir le salaire du directeur général pourquoi ils font pas les efforts pour qu’en suisse on paie meilleur marché les originaux comme c’est le cas à l’étranger parce que un original à Genève est plus cher qu’un original que j’ai acheté en France et vous trouvez normal ?

(alors maintenant si vous je vous demande vous dites en somme vous utilisez le mot imitation alors si je comprends bien les génériques seraient une imitation ?)

je pense c’est une imitation mais comme on a pas le droit d’imiter à 100 % un produit puisque je fais aussi les contrefaçons dans mon métier il doit y avoir quelque chose de différent qui empêche d’être attaquer en justice par les grandes sociétés pharmaceutiques et je pense qu’il doit y avoir quelque chose d’autre légèrement différent pour pouvoir le vendre

(donc en somme on serait plus proche de quelque chose d’une imitation come vous dites contrefaçon que comme quelque chose de véritablement identique ?)

oui mais on pas le droit de faire une copie identique on a pas le droit d’imiter à 100% un objet surtout si il est protégé donc il doit y avoir un petit truc une petite chose qui diffère et j’ignore laquelle c’est mais elle doit exister je pense

(il y a des gens qui disent que les génériques c’est des pales copies ? est ce que vous seriez d’accord avec ça ?)

……….écoutez si je me base sur l’antibiotique que j‘ai eu en générique c’était pas une pale copie puisqu’il m’a fait j’avais des problèmes au colon il m’a fait un excellent travail donc ça peut pas être une pale copie au contraire ça m’a ça a pris le temps exact comme un original pour me me me …..euh guérir des problèmes de colon donc ça veut pas dire que les génériques sont mauvais non plus ……mais il y a quelque chose que je sais pas est ce que ils ont le droit d’imiter à 100 % un produit X et en faire un produit Y l’appeler simplement autrement je sais pas ………

(on y reviendra après)

oui je me renseignerai auprès de mon beau frère

(alors justement vous avez votre beau frère est ce que vous avez d’autres moyens de vous renseigner ?)

à la pharmacie je peux poser la question

(vous poser la question ailleurs encore ?)

à mon médecin bien sûr, mon médecin, la pharmacie je vois pas à qui d’autre

(est ce que vous avez d’autres moyens pour vous renseigner ?)

euh……..téléphonez chez vous peut-être à l’hôpital et poser la question

(je pensais je sais pas par exemple est ce que vous lisez la notice ?)

lire la ?

(la notice d’emballage)

oui bien sûr et faire une comparaison entre les deux médicaments

(donc vous quand vous prenez un médicament vous lisez la notice ?)

toujours toujours à cause des effets secondaires et voir qu’ils peuvent pas être approprier vous comprenez

(alors à propos des effets secondaires est ce que vous pensez que c’est la même chose entre l’original et le générique ?)

je ne sais pas

(d’accord alors on revient aux informations, est ce que ça vous arrive de regarder sur internet lorsque vous prenez un médicament ?)

non honnêtement pas non

(vous utilisez internet pour d’autres euh .. ?)

pour mon travail mais j’ai jamais commandé un médicament sur internet sachant qu’il y a beaucoup de charlots là dedans et des vulgaires imitations qui peuvent être dangereuses donc j’estime déjà pas normal qu’on laisse vendre des prétendus médicaments sur internet je suis bombardé par ceux qui veulent vendre du viagra des crédits des machins c’est une horreur on est assommé par ces gens alors il y a des gens qui finissent par dire pourquoi pas et ils vont tomber sur n’importe quoi sur une pilule bleue avec du sucre dedans

(alors là vous dites ce sont de vulgaires imitations ?)

à mon avis oui

(ça c’est autre chose ?)

ça c’est autre chose oui ce qui se vend sur internet alors peut être il y en a 1 ou 2 % qui sont des bons médicaments mais le reste pour moi c’est de l’attrape nigauds

(alors je pensais pas tellement à acheter des médicaments mais il y a des sites qui donnent des informations)

j’ai jamais été dessus jamais non

non moi le médecin il me donne un médicament je l’achète je lis et après je le prends et si je sens que ça va pas j’avise le médecin et on essaie de trouver une solution

(vous lisez donc vous dites la notice d’emballage)

je lis toujours la notice d’emballage oui

(vous les gardez toutes ?)

je les garde jusqu’à que je termine le … mon traitement et si il y a pas besoin de recommencer je balance oui

(si je vous demandais est ce que les médicaments génériques sont soumis aux mêmes contrôles que les médicaments originaux ?)

par l’office euh…..(par exemple)

j’ose espérer que oui

(et vous savez qui les fabrique ?)

bah il y a mepha et puis dernièrement j’ai vu à la télévision il font un bombardement avec helsa je sais plus quoi est ce que c’est l’assurance qui fait ça je sais pas mais je sais que c’est jours-ci c’est plein c’est régulièrement ils nous donnent ce nom qui apparaît je connaissait pas mepha c’est plus connu avec l’arc en ciel

(donc ça c’est une entreprise pharmaceutique ?)

je pense qui oui du moins j’espère

(vous même si votre médecin vous prescrivait un générique est ce que vous hésiteriez à le prendre ou vous le prendriez sans hésitations ?)

non je le prends sans hésiter si le médecin me le prescrit par contre si le médecin me prescrit un original et qu’à la pharmacie on me dit nous avons ce générique est ce que vous le voulez j’appelle le médecin pour être sûr que je peux le prendre comme ça il y pas vous savez j’estime que ma santé passe avant alors j’appelle le médecin j’ai son numéro toujours là sur moi et si il me dit que il y a pas de problème ou c’est la pharmacienne qui appelle j’ai une très bonne pharmacienne qui est vraiment très humaine très proche et qui n’hésite pas à appeler le médecin si il le faut pour voir si on peut donner un générique

(donc c’est plutôt le rôle du médecin de décider pour le générique c’est pas celui du pharmacien ?)

à mon avis. C’est lui qui connaît le dossier du malade, bon le pharmacien lui il garde dans l’ordinateur tout ce qu’ils prennent souvent j’ai eu le cas ou il m’a dit non vous pouvez pas prendre ce médicament parce que vous prenez un autre et là le médecin n’avait pas c’était pas le même médecin c’était le médecin pour le yeux il savait pas que je prenais ce médicament donc la pharmacienne a très bien réagi aussi

(mais donc la prescription du générique pour vous c’est plutôt le médecin ?)

je pense que oui si maintenant moi j’achète moi même un médicament sans médecin qui est à la vente libre et qu’on me propose un générique moi j’accepte

(d’accord donc c’est plutôt pour le médicaments sur prescription ?)

exact (ou vous voulez l’avis de votre médecin pour savoir si oui ou si non ?)

exact (plutôt que celui du pharmacien ?)

en premier c’est le médecin maintenant si le médecin n’est pas atteignable ou que le pharmacien me convainc que c’est la même chose que je peux y aller les yeux fermés comme je vous ai dit comme je connais très bien cette pharmacienne c’est une amie j’accepte et je le fais parce que je sais qu’elle connaît bien son travail

(d’accord donc en fait c’est l’affaire que vous soyez convaincu que c’est exactement la même chose ?)

oui oui c’est une question de confiance oui

on voit vous savez on voit la différence entre une pharmacie ou il y a personne qui vous répond qui connait son sujet et ou il y une pharmacienne qui connaît pas très bien son sujet et qui doit attendre vérifier cette pharmacienne va pas vous proposer des génériques qui à mon avis elle devrait quand même à mon avis essayer de le faire avec l’accord de la pharmacienne ou du pharmacien mais…toutes les pharmacies ne le font pas est ce que ils gagnent plus avec les médicaments originaux qu’avec les génériques c’est possible je ne sais pas je connais pas la part qui revient

(mais alors vous au fond qu’est ce qui vous fera décider pour le générique ou plutôt pour l’original ?)

très honnêtement si ça fait baisser les prix pour la sante je le ferai parce que on dépense des milliards et parfois inutilement parce que toutes les assurances ne font pas attention à tout parce que quand vous rentrez à l’hôpital et que c’est passé minuit et que ils vous font payer 2 jours l’assurance elle vous dit que c’est pas son problème j’estime que les assurances elle ne vont pas du tout en suisse elles sont très chères elles ont des réserves excellentes elles en profitent au maximum et leurs patrons se remplissent les poches moi j’avais avant l’assurance accordia faites par les médecins et les pharmaciens qui était une excellente jusque XX a eu sa peau quand il était conseiller fédéral c’est un gars excusez moi qui a fait beaucoup de tord au système de la santé en suisse et ce monsieur tout simplement et personne nous le disait était avant de devenir conseiller fédéral était le secrétaire de la mutuelle valaisanne et sous fils est le secrétaire de la mutuelle valaisanne actuellement donc c’est scandaleux de rentrer dans ces choses-là c’est très valaisan cette façon de faire ça va trop loin ça va trop loin on a coulé avant je payait moins de 500chf avec la complémentaire en privé actuellement je paie 1390 chf par mois d’accord merci XX.

On a eu deux abrutis l’un après l’autre à la santé c’était XX et XX j’espère que maintenant il essaiera de nettoyer j’espère mais j’y crois plus parce que moi tout ce qui est politique je le met dans mon sac madame.

Parce que cette histoire de gros sous c’est possible, c’est possible

(ça vous est donc arrivé que votre médecin vous prescrive un générique ?)

oui c’est arrivé

(qu’est ce qu’il vous a dit quand il vous a prescrit un générique ?)

il fallait qu’il regarde et puis il lit, il lit l’autre et il dit c‘est la même chose est ce que vous êtes d’accord que je vous le donne ? j’ai dit il y a pas de problème du moment que vous me conseillez mais docteur B qui est un excellent médecin de médecine interne regarde toujours les deux dans son livre

(donc vous vous avez accepté quand il vous a proposé ?)

oui absolument et j’ai eu de très bons résultats à part deux le Flector et l’autre là à la place du Nexium je sais plus comment il s’appelait zurcan ? sarcan ? mais le Nexium m’a fait tout de suite de l’effet alors que l’autre pas j’avais toujours ces acidités

(vous m’avez dit que c’est arrivé que votre pharmacienne vous propose un générique et que vous préférez qu’elle appelle votre médecin )

si elle me garantit que c’est la même chose j’accepte parce que c’est une personne de confiance

(et qu’est ce qu’elle vous a dit quand elle vous a proposé le changement ? comment est ce qu’elle vous a proposé ça ?)

elle et sa collègue elles sont deux pharmacienne elles regardent et me disent attends parce que on se connaît depuis longtemps on se tutoie elle me dit attends je crois j’ai le même je vais aller voir et puis elle l’ouvre elle sort le mode d’emploi et me dit écoute c’est pareil tu peux y aller si tu veux il y pas de problème c’est le même mais c’est meilleur marché alors je dis ok on y va

(donc c’est sur la base d’une comparaison ?)

c’est toujours en comparant et on regarde non elles font bien leur boulot on a des bonnes pharmaciennes faut leur laisser…ce sont pas tous des rigolos qui veulent se remplir les poches

(donc votre pharmacienne quand elle regarde les deux et qu’elle compare c’est la même chose vous lui faites confiance pour les génériques ?)

tout à fait

(avec un effet positif ?)

oui c’est bien ça

(pour votre traitement actuel pour la douleur est-ce que vous prenez des génériques ?)

je crois pas qu’il y ai des génériques là dedans s’il y en a je suis d’accord, je prends de l’Oxycontin du 10mg j’en prends 20 le matin 30 le soir et ils vont me le monter à 15 et 30 et je prends de l’Oxycontin en gouttes pour essayer d’aider ça fait qu’empirer ce que n’a pas ……

et je prends Oxycontin oxedan, je prends Nexium je prends plavix et je prends Meto zerok c’est pour le cœur et de l’aspirine cardio je prends alors là les médecins me l’avaient donné pour le cholestérol un médicament chimique qui m’a pas du tout convenu on m’a même donné un générique mais j’avais des douleurs avec on l’a arrêté et la pharmacienne m’a donné qui s’appelle le HD quelque chose qui à base de produits naturels qui m’a fait un grand bien et aujourd’hui je l’ai là, la preuve que son médicament me fait pas mal et qui est un bon produit ça m’a baissé le cholestérol et c’est un produit naturel voilà donc vous voyez c’est vrai je suis toujours ouvert aux nouveaux produits, aux plantes, je suis très ouvert à ça j’y crois je dis pourquoi pas quel mal y a t il d’essayer si ça peut amener des bons résultats c’est que du bonheur pour moi

(donc là dedans c’est plutôt des médicaments originaux que vous prenez ?)

je prends je crois que c’est des originaux je sais pas et à part ça qu’est ce que j’aurais d’autre j’ai un Anxiolit donc je crois que c’est un générique c’était le seresta avant et j’avais arrêté et il va très bien on me le rembourse je prends rémérons mais là c’est un générique non ? puis c’est tout c’est déjà pas mal ?

(oui. Si pour l’un ou l‘autre de ces médicaments il y avait un générique est ce que vous aimeriez que votre médecin vous le propose ?)

oui, oui, oui tout à fait parce que quand j’ai vu le prix de ces médicaments ça vous dresse les cheveux c’est surtout pour le cœur les produits du cœur sont très cher je sais pas si le Plavix a un générique si le Meto zerok 125 je sais pas

(donc pour vous si il y avait un générique euh)

je serais ouvert oui

(vous seriez ouvert à changer si ça coute moins cher )

absolument oui si on peut baisser les couts de la santé oui vous savez déjà mon fils à qui j’ai payé 140 francs par mois il a eu 18 ans c’est monté à 409 non 450, 440 pardon c’est monté c’est de la folie la folie parce que 18 ans ça a multiplié par 3 on va ou là ?

(donc euh si on vous prescrit des génériques qu’est ce que vous aimeriez avoir comme information d’office ?)

d’office si ces génériques c’est adapté à mon problème de santé et ils ont les mêmes effets que les originaux donc je crains rien que je peux les prendre en toute confiance c’est ça et après à ce moment-là je les prendrai sans autre

(est ce que pour vous quand vous prenez un médicament vous avez des inquiétudes ?)

des inquiétudes ?

(oui est est ce que il y a quelque chose qui vous inquiète dans les médicaments ?)

……non

parfois le goût qui est mauvais à cause de la circulation on m’avait donné un truc qui a très mauvais goût, bon je suis un peu inquiet avec la cortisone je tiens pas à grossir parce que ça enfle je fais attention mais avoir des inquiétudes non vous savez j’ai eu 26 opérations 26 anesthésies donc et je suis pas dans le Guinness book

(pas toutes pour vos cervicales tout de même ?)

non non

(c’était pour des accidents… ?)

écoutez ça a démarré tout petit quand il vous endort là en faisant respirer de l’éther pour les végétations je me vois encore sur les genoux de ma mère, ensuite c’était l’appendicite ensuite j’ai eu une hernie hiatale à l’époque ils l’opéraient ils m’ont tout ouvert pour rien elle a repoussé, double fracture ouverte au judo quand j’étais à la police judiciaire avec une plaque métallique qu’ont m’a renlevée une année après re anesthésie j’ai vu là il ont bloqué deux orteils hallux valgus donc j’avais des problèmes d’arthrose ici, j’ai pété là de l’autre côté ils me l’ont aussi enlevé, j’ai eu hernies inguinales euh….j’ai eu plusieurs interventions pour la prostate finalement on l’a enlevée j’ai eu on m’a enlevé la première côte ici et c’était un faux diagnostic je faisais une thrombophlébite et ils ont dit que la première côte contre le muscle bloquait comme ça c’était n’importe quoi c’était un fameux moi je l’appelle charcutier qui s’appelle R. qui est décédé, 1 mois après j’ai fait une thrombophlébite j’étais le voir il m’a viré de chez lui 6h30 d’opération pour rien, j’ai été opéré des yeux de la cataracte etc etc là les hernies lombaires dorsales les 3, 1, 2, 3 la dernière opération le 8 janvier ils ont opéré le matin il a fait un très bon boulot monsieur M ils ont fait une hémorragie ils m’ont descendu au bloc ils pouvaient pas intuber ils ont ouvert à vif et pendant que monsieur R essayait de rentrer par le nez j’ai entendu dire merde je sais pas si je peux le rattraper et j’ai perdu connaissance j’en ris maintenant voilà il y a eu bon des petits trucs pour les bursites c’est incroyable

(donc vous avez le corps qui a été surchargé ?)

maltraité ouai ouai ouai je suis surpris qu’il tienne le coup

(alors moi je reviens à mes génériques c’est vrai que par rapport à tout ce que vous me dites là je comprends que vous me disiez que les médicaments ça vous inquiète pas trop vous avez eu toutes sortes d’autres expériences euh vous avez déjà pris vous des génériques contre vos douleurs ?)

non on m’a jamais donné

(on vous a jamais proposé des médicaments génériques contre la douleur ?)

non non

(s’il en existait est ce que si on vous en proposait est ce que vous seriez d’accord pour le traitement de la douleur ?)

oui tout à fait ah bah on sait jamais

(donc pour vous c’est pas du tout dépendant du type de maladie le fait de dire oui ou non aux génériques ?)

non non je suis prêt à essayer si ça peut me faire du bien je suis vraiment ouvert à toute proposition

(donc pour vous c’est pas du tout dépendant du type de maladie c’est plutôt comme vous le disiez si j’ai bien compris je résume juste pour être sûre d’avoir bien compris c’est une question de comparer bien l’original et le générique dans les indications ?)

voilà en espérant que ça puisse me faire du bien

(voilà mais ça vrai pour le générique comme pour l’original ?)

tout à fait

(c’est juste ce que vous ne voulez pas c’est une imitation ou quelque chose qui ne serait pas tout à fait la même chose c’est ça ?)

c’est peut être pas tout à fait le même chose mais aussi bon attention ça peut ne pas avoir un petite truc qui change je sais pas quelle est la loi normalement quand il y a une invention mais là pour les médicaments je sais spas si c’est la même chose est ce que les génériques ont le droit de faire exactement pareil qu’un vrai médicament enfin un vrai médicament l’original et le vendre sous générique j’ignore si c’est possible mais si le générique me fait du bien oui je le prends

(d’accord parce que quand vous dites vrai médicament qu’est ce que vous voulez dire ?)

je veux dire le médicament créé par XX etc qui a reçu l’accord de Berne et qui est sur le marché officiellement et que les médecins trouvent qu’il est bon et qu’ils le proposent le prescrivent voilà ce que j’appelle vulgairement un vrai médicament euh disons que je l’appellerai l’original mais l‘autre le générique si il est aussi bon que l’original et meilleur marché oui je le prendrais

(mais est ce que vous diriez que c’est un faux médicament ?)

un faux médicament ? ah non si c’est un faux je le prends pas

(d’accord ok alors j’arrive au bout de mes questions vous voulez ajouter quelque chose ?)

Non

**Patient 17**

(A votre avis, qu’est-ce qu’un générique ?)

Les génériques (‘nagériques’), c’est une photocopie, on peut dire comme ça, du vrai médicament, exactement la même chose, mais avec un autre nom… c’est moins cher… on peut dire comme ça…

(photocopie ?) photocopie, ça veut dire que c’est pratiquement le même mais avec un autre nom seulement.

(pratiquement le même ?) bonne question… ça veut dire…. Ils disent à la pharmacie que c’est exactement la même chose, mais il y a que le nom qui change, alors si c’est ça, ça veut que le médicament, c’est le même, c’est pas pratiquement le même, c’est le même, avec un autre nom c’est tout.

(‘à la pharmacie’ – et vous ?) moi je pense sincèrement si c’est moins cher, ça veut dire qu’il y a pas la même chose ! voilà c’est ça que je pense. ( ?) j’en sais rien je m’ai pas posé la question mais… peut-être qu’il y a des choses que c’est pas tout à fait les mêmes… sinon pourquoi il y a le générique et il y a pas vraiment le vrai ?

(substance ?) si vraiment le générique c’est la copie de le vrai, normalement ça doit être la même chose, mais comme c’est moins cher… alors je n’en sais rien.

(même effet ?) ouias, j’ai déjà pris les deux alors… pour moi c’est égal.

(mêmes EI ?) très peu que j’ai pris les génériques, mais quand je l’ai pris, c’était bon.

(lesquels ?) le Ponstan, j’ai pris l’autre le cium, quelque chose comme ça, pour moi c’était pareil.

(mêmes contrôles ?) voilà c’est par là que je voulais venir ! comme c’est le générique, je sais pas ! est-ce qu’il y a le vrai contrôle comme les autres, je sais pas. Mais je voudrais bien savoir, à la pharmacie ils me rassurent c’est la même chose ! alors c’est la même chose, alors moi je dis « pourquoi pas alors le même nom ? », « ah celui-là c’est moins cher ! », « alors pourquoi c’est moins cher ? » moi aussi j’ai fait la question à la pharmacie ! « ah parce que c’est le générique ! » mais ils m’ont pas expliqué.

(copie ? photocopie ?) non, c’est pas le vrai mot… il faut dire que… voilà, c’est le même avec un autre nom. (pas copie ?) ah ben non, c’est déjà une copie, c’est pas tout à fait le vrai… c’est déjà le deuxième, c’est pas le vrai !

(hésitations ?)… moi, j’ai pas hésité, on me l’a proposé et alors moi j’ai dit c’est la même chose, c’est le nom qui change, pourquoi pas, c’est moins cher. J’ai pas hésité, je l’ai pris, oui… ( ?) des fois, il y a des questions aussi, pourquoi c’est moins cher, ça vient toujours au même, ça veut dire pourquoi ils ont fait moins cher celui-là s’il y a exactement la même chose que l’autre ?! alors il y a quelque chose qui doit changer, certainement !.... mais je sais pas quoi…..

(à votre avis, qu’est-ce qui changerait ?) …. S’il y a une chose…. Les choses qu’on met dedans que c’est moins cher…. Qu’ils vont les chercher ailleurs, je sais pas… si c’est exactement le même, c’est les choses qu’on met dedans qu’on achète moins cher que quand on achète le vrai… Ou ça veut dire que c’est simplement la marque qui est plus chère, on va dire comme ça… ça doit être ça, c’est simplement la marque qui est plus chère… par exemple moi j’achète deux habits, un qui est de marque et un autre que ça lui ressemble qui est pas de marque, c’est la même chose mais c’est la marque qu’on paie ! [son téléphone sonne]. ( ?) c’est la marque… s’il est dans le marché, c’est qu’il est étudié et c’est pour ça qu’il est moins cher, à cause de la marque. J’espère qu’on va pas mettre un médicament mauvais ! On va dire c’est la marque qui fait ça.

(entendu parler comment ?) c’est la pharmacie.

(internet ?) non [ne lit la notice que si elle a oublié la posologie maximum]

(MD ?) ça je peux pas le dire, je me rappelle pas

(MD a prescrit ?) oui… (lesquels ?) je sais pas si c’est mon médecin traitant ou si c’est quelqu’un d’autre… mais certainement… je suis pas sûre. (ponstan-générique ?) … non je crois c’est à la pharmacie… qu’ils m’ont dit à la place de ça il y a le générique c’est moins cher… je crois c’est à la pharmacie. Ils m’ont dit c’est la même chose, il y a que le nom qui a changé. ( ?) j’ai pensé alors pourquoi pas essayer, c’était ça ma réaction, et j’ai demandé c’est exactement le même, ça va pas faire de mal ? ils m’ont dit ‘oui oui c’est la même chose’ ! il y a le même truc dedans, il y a que le nom qui change, alors j’ai dit ok je vais l’essayer. (autres génériques ?) je sais pas si j’ai pris un autre ou pas… [x] mais je connais des cas de ma belle-sœur elle refuse tous les génériques… elle dit tant qu’elle paie, au moins qu’elle fait le bon… au moins comme ça elle sait que c’est bon, et l’autre elle a un peu de doute.

(voudrait savoir quoi en plus sur génériques quand MD prescrit ?) alors j’aimerais bien qu’il me dise qu’est-ce qu’il y a dedans, que c’est pas grave pour la santé, c’est pour ça qu’il me le donne, il faut qu’il me rassure quand même, comme la pharmacie quand je suis allée. (pour original ?) alors je demande si c’est pour la douleur… si c’est bien ça que… s’il va me… quand je prends un médicament, qu’il me dise et moi je dis c’est ça que je dois prendre ? qu’est-ce qu’il y a dedans ? pourquoi je le prends, c’est pour cette douleur ? voilà, il m’explique quand même.

**Patient 18**

A votre avis, qu’est-ce qu’un générique ?)

Ce que c’est… par rapport au vrai ?... pour moi je pense que c’est le même médicament, juste ce qu’ils mettent dessus, les excipients ou les colorants… pour moi ça c’est différent, mais à part ça je pense que le médicament reste le même… je crois hein ! c’est ce que je pense…

(même substance ?) pour le médicament oui et après ce qui entoure le médicament… je pense qu’il y a plein d’autres… c’est fait différemment, c’est tous les trucs chimiques, les… ouais… L’extérieur, si on veut, l’emballage, la capsule… le truc colorant rouge, ça c’est différent

(même effet?)… oui… je peux donner un exemple ? par exemple le ponstan et le Méfenacid me font le même effet au niveau douleur, par contre le ponstan je le supporte, le Méfenacid je le supporte pas et je suis sûre qu’il y a du lactose dedans parce que ça me fait les mêmes douleurs que le lactose… alors voilà… c’est aussi pour ça que je pense que les petits… mais autrement au niveau efficacité de la douleur, quand j’ai mal à la tête, reste la même…

(pas mêmes EI ?) oui j’ai les effets secondaires, alors qu’avec le ponstan j’en ai pas… qu’avec Méfenacid, j’ai des douleurs comme si j’avais mangé du lactose sans mes pilules, sans mes anti-lactoses… donc je le prends plus, ça c’est sûr.

( ?) en générique, j’ai pris donc le Méfenacid que je prends plus, j’ai pris le Irfen, je crois que c’est le Brufen… que je prends plus non plus… comme génériques je crois que c’est tout. ( ?) je prends plus l’Irfen… en fait, je sais pas… je prends plus ( ?) juste parce qu’il me fait rien, en fait le Brufen me fait rien et j’ai pris une ou deux fois le Irfen… il me fait mal au ventre aussi… donc en fait c’est pas un médicament qui me fait du bien donc je le prends plus… mais en générique, je crois que c’est tout ce que j’ai pris.

(Copie ?) une copie pour tout ??... non moi je crois pas. Parce que quand je regarde, quand je prends le Ponstan, je me sens bien avec, j’ai ni mal au ventre ni… bon, des fois je dois prendre un [x] juste pour les brûlures… que le Méfenacid, je me sens vraiment pas bien avec… je crois pas que ce soit une copie… (copie= ?) exactement le même effet… et que moi je trouve que ça fait pas du tout le même effet !

(qui les fabrique ?) je sais pas…

(mêmes contrôles ?) j’en sais rien, mais je crois pas… moi je pense que non, mais… mais j’en sais rien… je pense que non… on se dit voilà on met ça, ça, ça donc ça fera le même effet que l’autre… pour moi, non, ils reprennent juste les bases mais ils font pas de contrôle dessus, à mon avis je pense que c’est ça… comme ils savent déjà que le ponstan fait tel effet, tel effet secondaire… donc ils prennent pas le temps de regarder la copie, pour eux ça reste exactement la même chose, donc à mon avis, c’est pas contrôlé (ils ??) ceux qui fabriquent… l’industrie qui fabrique !!

(MD prescrit ?) oui, donc le Brufen je crois que c’est l’Irfen et le… Méfenacid, mais qu’elle a arrêté… elle sait pas exactement si… mais moi je suis sûre qu’il y a du lactose dedans… donc c’est pour ça que je l’ai arrêté.

(si MD prescrivait un autre générique, hésitations ?) oui, c’est juste que j’ai très peur des médicaments, déjà pour prendre l’original, j’ai déjà peur, une copie entre guillemets exacte hein… eh ben, non… ! Voilà, je… il faut vraiment qu’elle me dise s’il y a pas de danger, bien tout m’expliquer, mais non je suis pas… comme avec le psy par exemple, avec le docteur C, …j’ai refusé de prendre le générique de l’antidépresseur… parce que j’ai peur, donc on est resté sur le… non, je crois que ça me fait peur de prendre déjà les médicaments alors les génériques c’est encore pire… ( ?) parce que j’ai eu tellement d’allergies que… j’ai tellement peur qu’ils mettent des substances qui sont poison dedans, moi je pense à ça, poison, donc ça me fait peur… (poison ?) voilà on revient sur la question de tout à l’heure, ils sont pas vérifiés, je suis sûre qu’ils sont pas vérifiés, donc on sait pas exactement les effets secondaires qu’il peut y avoir et tout ça ça me fait peur !... comme quand on entend à la télé, voilà, tel médicament a été retiré du marché…tout ça ça me fait peur, donc c’est vrai que je suis assez contre les génériques. ( ?) l’original, tout en faisant très attention mais… je préfère avoir l’original.

(Pharmacien propose ?) si le pharmacien propose ?.... je sais pas… je sais pas, est-ce qu’il connait bien mon dossier… il connaît bien mon dossier le pharmacien ?? J’ai pas tellement confiance moi, il faut vraiment qu’on connaisse mon dossier, j’ai trop subi de choses avec les médicaments… d’effets secondaires pour euh… non je crois pas que je ferais confiance… il fait vraiment que ce soit mon médecin qui… voilà ! je fais confiance à mon médecin ! Donc non, le pharmacien je crois pas…

(Quoi expliquer à d’autres personnes pas sûres?) moi je crois que… voilà, on donne l’original, on sait, on regarde les allergies tout ça… et quand on donne le générique, on fait pas attention à tout ça et il y a tellement de gens allergiques, par exemple comme moi au lactose, ou à d’autres choses… et ça on n’y fait pas attention… je pense que si… ‘voilà, vous prenez tel générique, alors je vais regarder vos intolérances et tout ça’… ben là, je crois que ça donnerait déjà plus confiance… ce qu’ils font pas en fait, on vous donne tel médicament… ‘alors vous prenez le générique parce que c’est moins cher pour l’assurance’ et tout ça… et en même temps on reste dans le flou du médicament, est-ce qu’on supporte vraiment tout. Voilà, moi je pense que c’est une chose qui devrait changer ça en fait. ( ?) qu’est-ce qui change exactement dans le médicament… mais vraiment expliquer tout… ça mettrait en confiance les gens… et au fur et à mesure le générique deviendrait… bien et après les gens le prendront facilement… que là, non, on reste un peu dans le vide… est-ce que c’est bon, c’est pas bon, est-ce que ça fait vraiment le même effet que l’autre… et je crois que c’est toutes les questions qu’on se pose…

(expérience Ponstan/ Méfenacid – tous comme ça ?) moi je crois que oui… parce que j’ai peur en fait de prendre… quand on me dit voilà on va prendre un générique, je pense au Ponstan au Méfenacid, ahhh tellement mal au ventre, je suis sûre qu’il y a du lactose dedans, donc je suis sûre que dans tous il y a du lactose… donc si je sais que l’original me fait du bien, j’ai pas envie de changer.

(renseignements comment ?) déjà avec ma doctoresse… chaque médicament qu’elle me donne… déjà elle ouvre son livre et on regarde tous les effets secondaires qu’il pourrait y avoir, pour moi, par rapport à mes allergies et tout ça… et pi après, moi j’avoue que, j’ai fait beaucoup de fois, je suis allée sur internet… et j’ai regardé les excipients du… maintenant bon, on trouve pas tout le temps, mais j’ai retrouvé, par exemple pour le Méfenacid, effectivement il y a du lactose dedans… et c’est pour ça que … maintenant, est-ce que c’est le bon truc que j’ai trouvé… est-ce que vraiment… mais c’est pour ça que je dis quand je pensais qu’il y avait du lactose dedans parce que j’avais tellement mal au ventre, ben ça m’a donné confirmation et voilà… je vais me renseigner comme ça en fait.

(internet ?) je regarde, je demande, par exemple, euh…la notice, on l’a, et après quand ils marquent excipients… je marque sur internet les excipients de, par exemple, tel médicament… Ponstan… et pi, en cherchant bien, on trouve… et, donc ça les ouvre, et on peut lire tous les excipients qu’il y a dedans, et j’ai vu Ponstan et pas de lactose… et j’ai cherché Méfenacid parce que je comprenais pas pourquoi j’avais mal au ventre avec, et j’ai regardé, et c’était marqué… ‘lactose’ ! Parce que j’avais demandé à ma doctoresse qui a pas su me répondre ce qu’il y avait vraiment comme excipients, donc c’est pour ça que j’ai fait une recherche… et j’avais raison ! Donc c’est vrai que maintenant je suis persuadée que tous les autres ont la même chose… maintenant c’est peut-être faux hein ! (internet pour plus d’infos sur médicament) oui, je regarde pas les effets secondaires ou les choses comme ça, c’est vraiment ce qu’il y a dedans. C’est tout ce que je cherche sur internet… c’est pour savoir pourquoi ces médicaments me faisaient mal, et l’autre qui est soi-disant le même, enfin, c’est plutôt l’inverse, le Ponstan me fait pas mal et l’autre qui est soi-disant le même me fait mal… voilà, c’est pour ça que j’ai voulu savoir, et j’ai trouvé.

(en général pour les médicaments sur internet ?) non, parce que j’en ai pas tant que ça des médicaments… quand le médicament me convient et que j’ai pas d’effets secondaires, je regarde pas.

(autres questions de santé ?) non… pas vraiment… je demande à mon médecin et pi euh… j’ai regardé une fois le glaucome, ce que ça faisait le glaucome, mais autrement non, je regarde pas…[x] il y a tellement de conneries, on a peur, il y a tellement de choses qui sont peut-être pas vraies que… je fais pas tellement confiance, donc je préfère demander à mon médecin, qui sait. On tombe aussi beaucoup sur des forums et tout ça et… il y a des gens qui disent des trucs, après ça peut nous mettre des trucs dans la tête, c’est un peu angoissant en fait… je préfère pas… Je demande à mon médecin, mon médecin me dit, et voilà.

[reprend le fait qu’elle pense que les génériques sont pareils aux originaux en substance, la base est la même] voilà je pense que l’original coûte plus cher parce qu’ils mettent peut-être des excipients de… plus chers, qui sont de meilleure qualité, et je pense que le générique, ils mettent des trucs… de moins bonne qualité !

**Patient 19**

(A votre avis, qu’est-ce qu’un générique ?)

C’est le même produit mais le laboratoire qui change… bon il y a le nom commercial, le nom d’origine… il y la laboratoire qui l’a fabriqué, après s’il y a quelqu’un derrière lui qui vient fabriquer le même médicament, il lui laisse le nom chimique mais il lui donne un autre nom commercial. Moi je sais comme ça.

(Le même?) ça veut dire il y le même effet.

(même substance ?) normalement c’est la même chose oui. Après la couleur ça change mais… après… normalement c’est le même produit. ( ?) il y a le laboratoire d’origine et le laboratoire, comment dire ça, pas d’origine, qui doit pas donner de nom d’origine.

(‘normalement’?) normalement ça veut dire… ça dépend, il y a des gens qui… je sais pas si c’est psychique ou… ça m’est arrivé moi de prendre des produits génériques mais… il y a d’autres contre-indications… ( ?) peut-être je supporte mieux le d’origine que le générique… ( ?) je sais pas, je sais pas si c’est psychique ou… question matière ou… parce que ça c’est pas le premier choix comme on dit… (‘pas le premier choix’?) c’est pas le produit d’origine… on dit c’est pas le premier choix, c’est différent… (différence ?) je sais pas, peut-être…. d’origine… peut-être il est 99% efficace que le générique… (plus efficace ?) oui, le d’origine il sera plus efficace que le générique ( ?) je sais pas… la différence de laboratoire, la différence de formule chimique…je sais pas… peut être c’est ça. (pas tout à fait le même ?) pas à 100% mais sur la feuille c’est la même chose ! sur la notice. (expérience personnelle?) D’ailleurs mes médecins ils m’inscrivent toujours l’origine, toujours de prendre le produit d’origine (quels médicaments ?) il y a le trittico, il y a… il y a pas mal de produits… par exemple le Lyrica… il y a pas mal… parce que moi je prends beaucoup de produits hein ! donc c’est ça. Les médecins ils me conseillent toujours le produit d’origine !

(votre avis ?) j’ai rien de contre… tant qu’ils me soulagent un petit peu… ils me calment un petit peu pour la douleur ou même pour la dépression… pourquoi pas ? parce qu’il y a le prix qui change aussi !... question le prix…. L’original il est plus cher que le générique… donc c’est ça… ( ?) moi qu’est-ce que je sais c’est pas les mêmes laboratoires, c’est pas le même emballage, c’est pas le même nom commercial, peut-être c’est à cause de ça… oui, je dirais.

( ?) j’ai pas une préférence, c’est mes médecins qui me disent !... ils me disent toujours ça.

(Si MD prescrit générique ?)… ben… je l’essaie… je suis pas contre… j’essaie… après si je vois que quelque chose que va pas, qu’il me donne un autre effet secondaire, là j’arrête, j’appelle mon médecin et je lui demande… je lui dis ‘est-ce que c’est psychique ?’ parce que c’est psychique aussi… (?) le cerveau… il lit pas le médicament la même chose, comme on dit hein !

( ?) je prends le médicament, je vois, si je vois que ça va sur moi sur mon corps, est-ce que mon sang il l’accepte… là il y a pas de problème mais si je vois que je me sens pas bien ou quelque chose, je stoppe. Je demande au médecin pourquoi j’ai ça, pourquoi avec l’autre j’ai pas ça, pourquoi ça, pourquoi ça…

(Si pharmacien propose générique ?) ça m’est arrivé déjà, à Meyrin, mon pharmacien il m’a déjà proposé un générique… (réagi comment?) normalement… ( ?) j’ai dit oui, que j’étais pas contre, j’ai toujours essayé, voilà… parce que eux ils disent c’est le même produit, c’est la même chose… c’est le même effet… mais des fois… (quel médicament ?) je me rappelle pas pour quel médicament, mais je me souviens une fois il m’a donné un générique le pharmacien… pour quel produit je me souviens pas parce que j’ai pris tellement tellement de traitements, tellement de produits… Par exemple j’ai les sachets pour l’estomac, comment ils s’appellent… [x] c’est un pansement gastrique…ben il y a en comprimés et il y a en sachet… et moi je supporte plus le sachet que le comprimé… le comprimé j’ai pris une fois… il y a pas d’effet, je sais pas….je sais pas pourquoi.

(pris générique proposé ?) oui… mais c’était pas tellement efficace… j’ai pris directement après l’original… c’est allé mieux…

(copie ?) oui ! on peut dire ça... c’est une copie du produit d’origine, parce que le laboratoire… l’original… ils donnent jamais la formule exacte… donc c’est pour ça c’est une copie…

(mêmes contrôles ?) non… on les contrôle moins, des fois on les contrôle même pas. Parce que des fois quand on voit des produits qui viennent d’Inde ou……. ou…. C’est pas la même chose. Moi je pense c’est comme ça… c’est pas fiable à 100%.... Moi je prends beaucoup de médicaments et j’ai que l’origine, que les laboratoires français ! [x] C’est une copie moi je pense…. Ou ça soulage peut-être à… 60%... des fois ça soulage pas… des fois c’est pas le même effet.

(explications pour confiance génériques ?) …. J’aimerais savoir pourquoi ils ont pas le même nom parce que moi je suis pas convaincu… je suis pas sûr à 100% comme j’ai dit… mais j’aimerais bien pourquoi… pourquoi ils ont pas le même nom… pourquoi… est-ce qu’il y a des contrôles à 100% pour ça… pour ces produits… et est-ce que… pourquoi ils font pas le même effet… pourquoi ça change… ça change de personne à personne… il y a des gens qui acceptent le générique… ils veulent pas l’origine ! et il y a d’autres, le contraire… Par exemple moi… avant je prenais le Tramal , Lyrica, Sirdalurd, brifen… pi après à force à force le brifen il a attaqué mon estomac, il a bousillé mon estomac… je l’ai stoppé [parle de son traitement. Prenait Tramal , stoppé récemment par sa psychiatre qui lui a prescrit du Trittico dont elle lui dit que devrait agir sur douleur et dépression, mais lui ne voit pas d’effet pour le moment, en tous cas sur la douleur].

**Patient 20**

(votre avis, qu’est-ce qu’un générique ?)

Les génériques, pour moi, c’est moins cher, mais il y a toujours les mêmes substances qu’il y a dans le médicament normal, sauf que c’est fabriqué moins cher ou quelque chose, je sais pas du tout… je suis pas sûre mais je pense que c’est pour ça que c’est moins cher… mais je pense qu’il y a les mêmes effets, sauf que c’est des fabricants différents… c’est la marque, je pense, c’et la différence on paie pour la marque l’autre

(même substance ?) oui, parce que je demande toujours quand je vais à la pharmacie, parce que les employés de la pharmacie me demandent si je veux un générique… comme j’utilise l’Effexor en générique pas le…. ils m’expliquent que c’est moins cher… parce que je lui demande parce que j’ai peur que ça a des effets comme les antidépresseurs, j’ai peur… j’ai peur beaucoup de prendre des médicaments avec antidépresseurs parce que j’ai déjà eu mal avec un antidépresseur et pi elle m’a dit ‘non c’est la même chose’, elle m’a montré pour me rassurer que c’était la même chose ( ?) oui je lui ai demandé pourquoi c’était moins cher pi elle m’a dit que c’est la marque.

(mêmes EI?)… elle m’a dit… j’ai demandé parce que moi j’ai peur des effets secondaires… j’ai peur de qu’est-ce qui peut m’arriver parce que j’ai eu des effets avec des médicaments que je n’ai pas supporté… donc aujourd’hui quand quelqu’un me propose un médicament en disant que c’est pareil, là je panique… pour pas avoir le mal que j’ai eu avec un médicament.

( ?) elle m’a dit que c’est la même chose sauf c’est le fabriquant qui change. Parce que moi je lui ai dit j’ai eu l’Effexor que la tablette c’était jaune et elle m’a donné un rose, j’ai dit non c’est pas la même chose, elle m’a dit oui c’est la même chose sauf que celui-là il est générique, l’autre c’est pas générique et elle m’a expliqué comme ça, c’est ça que j’ai compris.

(copie ?) je pense pas que c’est une copie, parce que… on dirait… je pense qu’on peut mettre les habits pareils… sauf que je peux acheter, c’est comme une copie… on peut acheter un tee-shirt que c’est de louis Vuitton et je peux utiliser le même modèle pour faire un autre sauf qu’il y a pas la marque louis Vuitton… c’est comme une réplique… (médicament ?) avec les habits on peut pas utiliser le même tissu, donc ça va changer le tissu, ça va changer la marque, beaucoup de choses… pour le médicament, je pense qu’on peut pas trop changer. (Donc pas copie) Comme le sac louis Vuitton, vous savez qu’il y a faux et qu’il y a vrai, donc si vous regardez il y a du faux qu’on dirait que c’est vrai mais sauf que la matière c’est pas la même, ça fait la différence et pi la marque qui est pas dessus, tandis que le médicament non, je vois pas la différence entre le générique et l’autre que j’ai pris.

(MD prescrit ?) oui, il m’a dit une fois je vous prescrit le générique, des fois ça change le nom du médicament parce que c’est générique, mais que ça fait les mêmes effets. Il m’a dit que c’est la même chose, ça va faire les mêmes effets, il m’a dit des fois c’est pas couvert par l’assurance, avec le générique, je suis couvert.

(Pharmacien propose ?) Ah toujours ! oui comme avec l’Effexor (prescrit par MD ?) c’est le psychiatre… mais je me souviens pas si c’était générique, la première fois j’ai pris celui-là du jaune et après la deuxième fois elle m’a donné le rose qui m’a pris l’attention et comme c’est des antidépresseurs je fais beaucoup d’attention… pour pas prendre beaucoup, pour pas oublier parce que je sais maintenant… je commence à comprendre que j’ai besoin…. (vérifié car AD?) oui parce que… non, avant c’était toujours générique, même dans mon pays, le Brésil, toujours le générique parce que c’est moins cher, mais comme j’ai commencé à prendre de l’efexor, j’ai vu la différence parce que la boîte c’est jaune, c’est… le normal… et le générique c’est violet-rose… et là j’ai dit tout de suite ‘mais c’est pas le bon !’ et elle m’a dit non… parce que avant j’ai pris 2x 75 maintenant je suis avec 100 et 50 et moi j’ai dit non c’est pas la même chose, je pense que vous vous êtes trompée parce que la boîte c’est jaune, et elle m’a dit ‘non, c’est parce que c’est générique’ mais ça fait les mêmes effets’, moi j’ai dit non, il faut… moi je commence à paniquer parce que c’est des antidépresseurs, j’ai peur… j’ai eu des problèmes [x]. Une fois que mon corps il rejette ça veut dire que ça va pas pour moi. J’insiste toujours, mais des fois je sais que ça va pas, j’arrête. Ça dépend du médicament… la jaune au début, ça me faisait mal, j’ai demandé au médecin, il m’a dit que c’est normal, j’ai pris, j’ai lu pour savoir si c’est des effets que j’ai… et pi après le deuxième, ça veut dire qu’avec le temps, deux mois, le deuxième c’est mis en place, ça a donné la continuation, ça veut dire que j’ai pas eu des problèmes… j’ai du pour prendre un médicament accepter d’être malade, accepter de prendre un antidépresseur… pour moi c’était bon, j’ai besoin… disons j’ai commencé à tout regarder, je me méfie, je veux pas perdre ma personnalité…

(renseignements comment ?) le médecin, parce que moi je pense si le médecin me donne un médicament, il sait ce qu’il est en train de faire… maintenant, j’ai confiance dans mon médecin traitant… donc lui je sais qu’il va pas me donner une chose pour me faire mal… s’il m’a dit c’est générique ça me fait du bien, peut-être il peut se tromper…normal… mais s’il m’a dit le générique il est meilleur pour vous parce que celui-là il est plus cher, il sait ma situation, parce que moi , je lui ai pas demandé de me donner un générique… vous comprenez ? donc s’il me donne un générique, c’est parce qu’il sait que pour moi ça va aller, donc on doit faire la confiance. Donc moi je lui ai pas demandé de me donner un médicament moins cher parce que moi je sais pas… des fois je lui demande ‘c’est couvert par l’assurance ?’ et il dit je vous mets ça parce que c’est mieux pour vous , parce que c’est générique, c’est moins cher et ça fait les mêmes effets.

(autres sources ?) la notice, des fois oui, des fois beaucoup ! pour savoir s’il y a pas de la cortisone, s’il y a je grossis beaucoup, je commence à faire attention, pour savoir ce qu’il va me donner comme effet collatéral, parce que j’ai eu beaucoup avec le reméron, donc ça m’a paniquée… alors j’ai commencé à regarder.

(internet ?) j’ai regardé le Reméron… et pi Cipralex… c’est toujours les antidépresseurs. Et puis pour la douleur… j’ai dit ‘ça me fait rien les médicaments’, le début ça me fait mal à l’estomac… je me souviens pas … j’ai regardé aussi Irfen… ( ?) pour savoir comment je peux rester, qu’est-ce qu’il peut me faire, s’il va changer ma personnalité, si je peux venir plus calme, qu’est-ce que ça peut m’aider quoi ! ( ?) google…. Après je vais là-dedans, je mets en portugais et puis en français… jusqu’à je comprends… parce que c’est un dictionnaire… [x] comme quand j’ai cherché la fibromyalgie, savoir qu’est-ce que c’est… au début, j’ai rigolé, j’ai pensé que le médecin est fou, je suis pas malade… mais après bon… on se rend compte c’est pas le médecin qui est malade, c’est nous qui doit accepter…

(hésitation pour générique ?) … c’est ça que j’ai dit concernant l’antidépresseur… les effets secondaires. [a stoppé Irfen, essaie de fiare sans médicaments, car peu d’effet] J’ai vu la notice c’est marqué que l’antidépresseur il peut m’aider pour la douleur, mais je pense que c’est faux… le début on dirait que ça change… je suis motivée… mais après… ko… [X] les génériques et les autres c’est pareil, j’en ai déjà pris le même, l’Effexor, ça a rien changé, mais j’ai dit au pharmacien que j’ai peur parce que j’ai pris l’autre, la tajette elle était jaune et pi il m’a dit non, vous inquiétez pas…

(qui les fabrique ?) non pas tout à fait

(Convaincre d’autres pas sûrs?) comme moi au début...( ?) ce que le médecin m’a dit, la pharmacienne elle m’a montré que c’était moins cher et puis elle m’a montré, si vous regardez, le médicament il a disons cortisone 100%, là aussi 100%... et pi, je dis un exemple, caféine à 20, et là aussi caféine à 20… et pi c’est comme cacao, chocolat, des fois c’est plus cher, mais je regarde et c’est tous pareils, mais pourquoi l’autre il est plus cher ? parce que ça change l’emballage, ça change la couleur… c’est comme m-budget… si vous regardez tout ce qu’il y a dedans, il y a pareil, mais il y a l’emballage pour faire recyclable, il y a pas pour faire joli, attirer l’attention du client… voilà ! j’ai trouvé la réponse.

**Patient 21**

(A votre avis, qu’est-ce qu’un générique ?)

Euh c’est le même médicament qui a les mêmes molécules que le médicament qui est sous le nom d’une marque c’est moins cher parce que il y a pas cette marque

(d’accord, vous pensez qu’il contient exactement la même substance que le médicament de marque ?)

Bah j’ai un certain doute par rapport à l’enveloppe du médicament

(qu’est ce que vous appelez l’enveloppe ?)

Quelque chose qui protégerait du milieu avant d’aller ou ça doit aller

(ok donc dedans il y aurait la même substance et autour il serait enrobé si on peut dire ?)

euh bah non j’ai ce doute par rapport à un médicament que je prends qui est la Dépakine et puis la pharmacienne m’a proposé le générique et en général je dis toujours oui et là en l’occurrence j’ai demandé si il y avait une différence parce que bon j’ai pas envie de … et elle m’a dit que certains clients avaient remarqué que ça convenait pas donc j’ai pas pris le risque

(d’accord donc vous pensez que il y a quand même une différence alors ?)

Euh non je suis pas sûre parce que on me dit le contraire qu’en fait ça doit être exactement la même chose donc…

(quand vous dites on c’est qui ?)

oui justement c’est pas vraiment c’est des connaissances

(vous avez entendu dire ?) oui voilà (c’est pas la pharmacienne qui elle vous a dit qu’il y a peut être une différence ?)

euh je me rappelle plus si elle est allé dans les détails

(et vous pensez que le générique est toujours aussi efficace que le médicament original ?)

J’ai toujours cru oui

(d’accord et que il a plus ou moins d’effets indésirables secondaires ?)

c’est là ou je me posais la question par rapport à l’estomac que ça pouvait faire plus d’acidité

(d’accord et est-ce que vous pensez qu’un générique est soumis aux mêmes contrôles du gouvernement ?)

oui (d’accord vous savez un petit peu quels sont les contrôles que fait le gouvernement ?)

euh non pas trop exactement mais je crois que c’est très bien surveillé

(pour vous c’est les mêmes contrôles ?)

oui

(euh si vous avez une expérience est-ce que vous pensez que les génériques sont plus difficiles à absorber, à avaler?)

euh ce que j’utilise souvent c’est le Irfen et ça me convient tout à fait pourtant on dit que ça peut aussi faire mal à l’estomac mais j’ai pas remarqué

(d’accord vous savez qui fabrique les génériques ?)

euh je pensais que c’était plusieurs boites différentes je sais pas (donc des firmes pharmaceutiques ?)

oui (vous pensez que ce sont des grandes firmes pharmaceutiques comme celle qui font les médicaments avec les noms de marque ?)

Bah j’ai remarqué que il y en avait une qui faisait de la pub donc non je pensais pas que c’étaient les mêmes qui faisaient aussi le générique

(d’accord vous avez vu quelle pub vous vous souvenez ?)

c’était Sandoz

(ok pour un générique) oui (d’accord ils faisaient de la pub uniquement pour le générique ?)

Bah c’est ce que j’avais retenu je sais pas si c’était l’inverse voulu mais bon

(d’accord ok )

(donc vous est ce que vous hésiteriez à prendre un générique ?)

non en général je prends le générique surtout qu’ils ont en principe moins chers

(d’accord sauf la Dépakine là vous avez pas suivi) non j’hésite encore je sais pas si c’est…..

(ok comment vous avez entendu parlé des génériques vous avez dit la pub est ce que il y a d’autres .. ?)

euh oui je sais pas euh ça remonte d’assez loin j’ai fait des études d’aide soignante et là quand on me parlait des génériques comme vous voyez j’ai pas tout retenu mais ouais euh …. plutôt positivement donc effectivement les autres médicaments on se demande pourquoi les médecins parfois prescrivent la marque d’office et puis qu’après à la pharmacie on nous donne le choix puis on se demande pourquoi (d’accord donc en fait vous vous préféreriez que le médecin prescrive directement le générique, est ce que ça vous perturbe d’arriver à la pharmacie et que le pharmacien en fait change l’ordonnance du médecin ?)

euh disons ça me rassurerait si le médecin le mettait tout de suite mais c’est aussi que il y a plusieurs sortes de génériques pour un médicament

(c’est pas facile oui)

(est ce que vous êtes allé chercher des informations sur les génériques sur internet par exemple ?)

non

(est ce que parfois vous allé cherché des informations sur les médicaments sur internet ?)

non plutôt sur les problèmes de santé

(d’accord pour quels types de problèmes de santé vous êtes allé sur internet ?)

euh par rapport à la douleur, des opérations que j’ai du subir

(vous avez un site de santé sur lequel vous aimez allé ?)

non, je tape plutôt le sujet que j’aimerais avoir

vous tapez sur quel moteur de recherche plutôt ?

google

(Google ok, donc vous mettez par exemple une maladie sur google et vous regardez ce qui vient ?)

Après je sélectionne Je regarde ce qui est sérieux, et je vois

(Comment savez vous ce qui est sérieux)

Je regarde plusieurs choses

(Donc vous ouvrez plusieurs sites et vous comparez, c’est ca qui vous dit ce qui est sérieux ou pas)

Oui mais je me méfie quand même ce qu’on peut avoir comme renseignement…y’a quand même le docteur que mon médecin dr C avait mis en ligne

(C’est lui qui a fait le site ?)

C’est son groupe oui

(Je connaissais pas d’accord)

Oui c’est un site que j ‘ai l’habitue de regarder

(Est ce que la pub sur les génériques c’était dans des journaux, des placards pub, télé ? Affiches en ville)

Oui ile me semble aussi les avoir vus (ok.) (Votre médecin vous a déjà prescrit des génériques ?)

Euh…probablement mais je suis pas sur…je sais plus lesquels… oui enfin c’est à ma demander de renouveler l’Irfen, ça il marque directement

(Pour l’Irfen il marque directement mais c’est pas lui qui l’a mis en premier)

je sais plus ça

(Est-ce que il vous a expli…)

Excusez moi, à l’hôpital on prescrit l’Irfen ?

(Oui oui)

Alors ça vient peut-être d’ici

(On vous a expliqués à l’hôpital qu’on donnait des génériques ou pas ?)

Je sais pas, on m’a dit que c’était un anti inflammatoire mais voilà

(Et avec votre médecin vous avez abordé le sujet)

Non non je crois pas

(Et votre pharmacienne par contre oui hein il a abordé le sujet, avec en particulier la Depakine)

Oui elle elle m’a dit à force de voir que je prenais souvent de la Dépakine elle m’a demandé si ça m’intéressait de changer

(Mais elle vous a pas vraiment rassuré en fait, on peut dire ça)

Oui, en fait au départ j’étais pas tellement rassurée de prendre des médicaments différents de mes médicaments habituels

(Oui d’accord, qu’est ce qui vous permettrai de vous décider, de prendre par ex la Dépakine en..)

Par exemple en ce moment j’ai des problèmes d’acidité gastrique, je me dis que si c’est la Dépakine pourquoi pas essayer le générique

(Pour vous il faudrait que le médecin se mette dans la discussion et donne son avis est-ce que ce serait quelque chose qui vous permettrait de vous décider ou que quelqu’un vous dise, que ce soit le pharmacien ou le médecin)

Je voudrais bien que ce soit le médecin

(Vous voudriez que le médecin prenne l’initiative)

Oui que ce soit l’un ou l’autre parce que moi je sais pas

(D’accord vous voudriez qu’on vous dise que la Dépakine générique ait moins d’effet sur l’estomac)

Non mais que les deux soit équivalent disons et que je vois un avantage à prendre le générique plutôt que la marque

(Vous voudriez un avantage physique plus que seulement économique)

Non plus juste économique, si on me prouve que c’est équivalent autant prendre le générique.

(Ok, donc effectivement il existe un générique pour la Dépakine, peut-être que je vous dirai deux mots après. Donc en effet vous avec une expérience avec les génériques, avec un antalgique, avec l’Irfen, vous aviez pris du Brufen avant une fois dans votre vie.)

Oui je pense, mais pas longtemps donc je me rappelle pas tellement.

(Mais vous avez pas eu l’impression que l’Irfen moins efficace)

Non

(Ou qu’il y avait plus d’effets indésirables)

Non

(Et le Brufen ne faisait pas plus d’atteinte sur l’estomac que l’Irfen par exemple)

Non je me rappelle pas tellement

(Quand vous avez su que l’Irfen était un générique, qu’avez vous pensé)

Ca m’a rien fait du tout

(Vous avez pas hésité à le prendre)

Non je suis plutôt pour les génériques

(Autre chose à ajouter sur les génériques)

Y’a juste une fois on m’a proposé un médicament en générique, j’avais rien contre les génériques alors j’ai pas hésité à le prendre, mais le générique était plus cher que le médicamentent, finalement c’était une erreur d’étiquetage donc je me suis demandé si chaque pharma décidait de ses prix, ou si c’était moins cher à l’étranger

(Mais finalement vous avez quand même pris le générique)

Oui oui c’était effectivement moins cher

**Patient 22**

(A votre avis, qu’est-ce qu’un générique ?)

A mon avis c’est un médicament c’est une copie d’un médicament où il a perdu ses droits pour être le seul en fait d’exclusivité

(D’accord, donc c’est la même molécule ? c’est le même médicament ?)

Ah ça je sais pas

(D’accord)

Apparemment ils changent quelques formules quelques additifs dans les formules je sais pas du tout je sais pas du tout

(Mais alors la différence entre celui qui avait l’exclusivité et le générique ou plutôt la ressemblance ?)

Ben c’est la recherche celui qui a l’exclusivité c’est celui qui l’a découvert en quelque sorte qui l’a mis au point et le générique il vient longtemps après quand le premier ait censé s’être remboursé ses frais de recherche du moins c’est ce que j’avais compris…c’est un peu comme des droits d’auteurs qui sont échus c’est ce que j’avais compris

(Et qu’est-ce-que cela impliquerait alors de votre point de vue ?)

Ca impliquerait qu’il y ait plus les frais de recherche remboursé donc c’est moins cher y a plus que des coûts de fabrication y a plus d’études à financer pour mettre au point le médicament vu que ça déjà été fait

(D’accord. A votre avis, le générique est-ce-qu’il est aussi efficace, moins efficace …ou la même efficacité ?)

Ben il y a pas de raison qu’il soit moins efficace moi on m’a dit qu’il y avait eu peut-être des problèmes avec les additifs qu’il devait changer dans la formule et qui était apparemment pas ce qu’était actif dans le médicament c’est ce que j’avais entendu une fois mais j’sais pas si c’est vrai

(D’accord. Est-ce-que en termes d’effets secondaires, y a de votre point de vue est-ce-que le générique a des particularités vous pensez ?)

Je pense pas

(Non ?)

Non j’pense pas

(C’est les mêmes que celui du médicament du départ ?)

Je pense

(D’accord. Moi j’y pense aussi parce que vous parliez de différence liée aux additifs)

Mais euh…j’peux pas vous dire franchement

(D’accord. A votre avis, est-ce-qu’il est soumis au même contrôle que l’original par les autorités qui s’occupent des médicaments ?)

Je sais même pas si les médicaments ont un contrôle suivi une fois qu’ils ont été autorisés sur le marché j’en sais rien franchement j’me suis jamais posé la question (rire) j’me suis jamais posé la question j’pense qu’une fois qu’il est autorisé sur le marché le médicament il a peut-être de temps un temps un contrôle sur la fabrication mais comme pour la nourriture ou n’importe quoi mais j’pense que pour les génériques ça doit être pareil il me semble que je vois pas pourquoi la loi suisse ferait une différence entre les deux…j’pense que tout est soumis au même régime j’espère

(Y a des gens qui disent que les génériques sont plus difficiles à avaler)

Moi je sais même pas si j’ai déjà pris des génériques il me semble que l’Irfen est un générique du Brufen donc là j’ai pris de l’Irfen j’ai vu aucune différence euh vraiment

(Tout pareil ?)

Oui

(D’accord)

(Vous-même si on vous parlait de générique, qu’est-ce-qui vous ferait hésiter à prendre un générique ?)

Euh ben justement j’hésite bon chaque fois qu’on m’en a proposé je les ai acceptés mais ce qui pourrait me faire hésiter c’est effectivement les effets secondaires ou un manque d’efficacité du médicament ou des effets secondaires non désirables mais pour l’instant j’crois que j’ai à chaque fois qu’on m’en a proposé accepté

(Vous dites ce serait pour des effets secondaires ou pour un manque d’efficacité, ce serait différent avec les génériques qu’avec le médicament dit original ?)

Moi je pense pas

(Vous auriez la même réaction, les mêmes hésitations dans les deux cas ?)

Ouais je vois de toute manière prendre un médicament ce n’est jamais innocent et puis…

(Dans quel sens ?)

Ben quand on lit les notices dans les emballages on voit les effets secondaires c’est pas vraiment réjouissant on espère que c’est sur une minorité de patients que ça ait lieu mais…j’pense que c’est de façon que ce soit un générique ou un médicament normal en tout cas la première fois qu’on le prend on se demande toujours si on va avoir des boutons des plaques ou un problème

(Vous lisez souvent la notice ?)

Ca m’arrive oui

(Quand c’est un nouveau médicament c’est de temps en temps, une fois sur deux, toutes les fois ?)

Par exemple si c’est quelque chose de tout nouveau ça peut si j’ai l’impression que c’est quelque chose d’assez fort oui je lis (vous lisez) si c’est une espèce d’aspirine ou quelque chose comme ça… même un antidouleur je lis pas

(Donc ça dépend de la puissance que vous pensez que le médicament peut avoir ?)

Oui

(Quand vous dites fort, c’est dans le sens, c’est dans quel sens en fait ?)

C’est dans le sens que pour moi un antibiotique c’est plus fort qu’une aspirine

(D’accord)

Un Ponstan c’est plus fort qu’un analgifor puis je pense qu’il y a encore plus fort qui fait encore plus de dégâts sur le corps…par force c’est plus lié par rapport aux effets secondaires

(D’accord)

(Alors si maintenant je vous demandais comment vous avez-vous entendu parler des génériques ?)

Par les médias

(Oui ?)

Ouais je pense

(Vous vous souvenez quel type de média c’était plutôt la tv, la radio, internet ?)

Non ça devait être ou la télé ou le journal papier

(Est-ce-qu’il vous est arrivé à vous d’aller chercher des informations à propos des génériques sur internet ?)

Non pas du tout

(D’accord)

(Est-ce-que vous êtes déjà allé chercher d’autres informations de santé sur internet ?)

Oui

(Des choses qui se rapportent….plein ?)

Oui

(Qui se rapportent à votre douleur en particulier)

Euh non par rapport à d’autres choses

(D’accord)

Par rapport à des maladies virales des choses comme ça que j’ai été voir des cancers ou des choses comme ça mais j’y vais plus parce que je me suis vite rendu compte que c’est la foire et qu’il a vraiment de tout et n’importe quoi

(De ce que vous dites j’ai l’impression, enfin vous dites que ça a finalement ajouté de la confusion plus que ça n’a répondu à vos questions ?)

Absolument ah même c’était pas j’ai compris très vite que n’importe qui pouvait répondre n’importe quoi et que le peu que je savais dés fois j’avais des textes ou c’était complètement le contraire du peu que je savais…on voit tout de suite si c’est des hypocondriaques ou…bref y a de tout et n’importe quoi et puis c’est loin d’être sérieux

(Est-ce-que votre médecin vous en a déjà prescrit ? Vous m’avez dit oui l’Irfen ?)

Mais je sais plus alors moi y’a des médecins qui m’ont prescrit dans ce cas dés fois je savais même pas que c’était des génériques et dés fois en pharmacie on me proposait le générique à la place quand je présentais mon ordonnance alors dés fois c’était par manque de stock je crois et d’autres fois c’était par souci d’économie en pharmacie

(Et qu’est-ce-que vous en avez pensé ?)

Moi je pense que c’est très bien j’pense que….j’pense que c’est une des manières de réduire les coûts de la santé qui me parait logique quoi….j’pense que les boites pharmaceutiques ont même assez d’argent donc au bout d’un moment ce serait bien qu’ils en fassent un peu moins j’sais pas (rire)

(Mais quand on vous a prescrit un antidépresseur ou quand la pharmacie vous a proposé un générique…)

J’ai pas d’antidépresseur

(Quand le médecin vous a prescrit un générique ou bien quand la pharmacie vous a proposé une substitution du médicament de l’ordonnance par un générique, est-ce-qu’on vous a dit quelque chose à ce sujet, on vous a donné une explication ?)

Non on m’a dit juste qu’il y avait un générique qui existait si ça me dérangeait pas de le prendre à la place…alors en pharmacie dès fois c’est ouais on est désolé on n’a plus l’original en stock par contre on a le générique et puis sinon c’était ouais ça vous dérangerait pas de prendre un générique…mais ce que c’était exactement non on m’a jamais rien expliqué

(Quand le médecin vous a prescrit ou quand le pharmacien vous l’a donné, on vous a jamais expliqué pourquoi un générique, c’est quoi ?)

Non

(Vous trouvez que c’est nécessaire ou pas d’avoir cette explication ?)

moi je pense que le patient qui a besoin d’une explication il est assez grand pour la demander...ce n’est pas non plus quelque chose que le médecin doit systématiquement avoir une discussion d’1/4 d’heure avec un patient pour expliquer ce que c’est un générique…maintenant…on peut pas demander à tout le monde aussi d’être curieux là-dessus..moi j’essaie de faire confiance au système de la santé…je peste contre les assurances maladie c’est un autre problème quoique c’est un peut le même problème aussi c’est une histoire d’argent finalement ces génériques c’est pas quelque chose d’autre qu’une histoire d’argent histoire de coûts de la santé

(Donc quand on vous a donné ou prescrit le générique, vous l’avez pris ?)

Toujours

(Vous avez jamais hésité à… ?)

Non

(Est-ce-qu’actuellement vous prenez un traitement sous forme d’un générique ?)

Non

(Aucun ?)

Aucun

(Vous ne prenez aucun traitement ou vous ne prenez aucun traitement générique ?)

Aucun traitement

(Ah, vous ne prenez aucun médicament actuellement. D’accord. Donc euh l’expérience que vous avez des médicaments génériques pour la douleur, c’est en particulier l’Irfen ? C’est juste ?)

Ouais mais sinon j’sais même pas si j’ai eu des génériques

(D’accord)

J’vous dis dès fois peut être que le médecin m’a prescrit des génériques sans m’en parler euh j’en sais rien

(D’accord. Donc l’Irfen vous savez que celui là vous l’avez eu, ça eu le même effet ?)

J’dirais que c’était aussi inefficace que l’autre

(Voilà, donc vous avez eu la même absence d’effets ?)

Exactement

(D’accord)

c’est que c’était pas le bon médicament pour ce que j’avais enfin tout simplement…dans les antidouleurs par rapport à ce que j’ai eu le seul qui était efficace c’est le Ponstan…pendant une courte période

(Mais là vous ne le prenez plus non plus ?)

Non…non parce que je sais que les effets secondaires sont…je veux pas en prendre un par jour toute ma vie…c’est pas pour des longues périodes c’est pas bon quoi

(Qu’est-ce-que vous faites alors actuellement contre vos douleurs ?)

Pour l’instant ben j’ai…j’ai finis tous mes examens et puis on va commencer un traitement du centre de la douleur

(Donc là vous allez commencer quelque chose de tout à fait enfin un traitement différent de ce que vous avez eu jusqu’à présent sur la base des investigations qui ont été faites ?)

Oui

(D’accord. En ce moment par exemple, vous avez une douleur de combien sur une échelle de 0 à 10 ?)

Maintenant ?

(Là ?)

3-4

(3-4. Ce matin c’était à combien ? au lever on va dire)

quasiment 0

(Quasiment 0, bien. C’est habituel ou c’est une journée particulière ?)

Habituel

(D’accord. Donc vous vous levez plutôt bien le matin ?)

Oui…non mais là c’est juste parce que je suis assis

(Ca vous convient ?)

Non…ouais c’est mieux que coucher

(Coucher c’est le plus difficile ?)

Assis ça va moins mais debout y a pas de problème

(D’accord, ça toujours été comme ça ?)

Depuis…euh la première crise j’avais mal partout tout le temps

(D’accord. Et ça c’est amélioré avec, je veux dire ça été l’évolution naturelle ou bien c’est…)

Non j’ai eu un traitement antibiotique qui m’a complètement remis normal pendant quelques mois puis la douleur est revenu beaucoup plus doucement par derrière…au bout de quelques mois ça fait tellement longtemps que je pourrai pas vous dire exactement les périodes

(Et donc, un seul médicament efficace le Ponstan ?)

Mais j’sais même pas s’il serait toujours efficace

(Et pour celui-là vous avez peut-être eu un générique mais vous ne le savez pas on va dire ?)

La cortisone…

(La cortisone aussi. Pour ces médicaments, vous avez peut-être eu des génériques mais ça ne vous a pas frappé ?)

Ponstan ça existe en générique ?

(Ecoutez, je ne saurais pas vous répondre)

Moi non plus donc je sais pas peut-être

(D’accord. Pour l’ibuprofène vous avez eu l’Irfen, vous aviez eu aussi du Brufen avant ?)

Oui

(D’accord. Et là comme vous disiez vous aviez eu la même absence d’effets ?)

mm

(Et vous avez eu des effets indésirables ?)

Non

(Ce n’était juste pas efficace ?)

Oui

(C’est pas qu’il y avait des effets particuliers de l’Irfen ? D’accord)

(Ben écoutez, on arrive au bout, vous voyez y avait pas tellement de questions que ça.)

Fin de l’enregistrement

**Patient 23**

(A votre avis, qu’est-ce qu’un générique ?)

C’est un médicament qui est meilleur marché qu’un original

(C’est la même chose ?)

Ben tout le monde dit qu’en principe c’est la même chose

(« En principe » ça veut dire que vous avez des doutes ?)

Non, j’en sais rien du tout

(Vous pensez qu’il contient la même substance ?)

Oh je pense que oui

(D’accord. Qu’il est toujours aussi efficace, qu’il est aussi efficace que l’original ?)

Je ne sais pas parce que moi je ne suis pas habitué aux médicaments parce que ceux que j’ai eu je sais pas si ce sont des génériques ou bien des originaux ça je sais pas

(Qu’est-ce-que vous avez eu ?)

Euh Lyrica (ça c’est pas un générique) Brufen… qu’est-ce-qu’il y a eu euh rivotril euh j’me rappel plus quoi

(Donc on vous a jamais dit « on vous prescrit un générique « ? Votre médecin ne vous a jamais dit « là je vais vous prescrire un générique » ?)

Ben c'est-à-dire que lui je pense que si y a des médicaments qui sont génériques et qui ont la même valeur il le dit et il me le propose

(D’accord, donc il vous a déjà proposé des génériques ?)

Oh oui oui toujours

(D’accord. Pour quels médicaments, vous vous souvenez ? Pour lesquels il vous a prescrit le générique ?)

Non aucune idée

(D’accord. Donc vous en avez déjà pris vous ?)

J’sais pas lesquels

(D’accord, ok. Est-ce-que vous avez l’expérience ou entendu dire que les génériques pourraient faire plus d’effets indésirables que l’original ?)

Non, non j’ai jamais entendu dire ça

(D’accord. Vous ne l’avez pas vécu ?)

Non

(Pensez-vous que les génériques sont soumis aux mêmes contrôles par le gouvernement que l’original ?)

J’en sais rien j’pense que c’est le même système

(D’accord, vous savez quel système ?)

Non

(D’accord. Est-ce-que selon votre expérience ou est-ce-que vous avez entendu dire que les génériques sont plus difficiles à avaler que l’original ?)

Non pour moi une pilule c’est une pilule euh ce qu’il y a dedans…

(D’accord, vous savez qui fabrique les génériques ?)

Oh je pense tous les grands produits pharmaceutiques (les grandes firmes pharmaceutiques) j’sais pas il faudrait demander à Couchepin peut-être

(D’accord. Est-ce-que vous, vous hésiteriez à prendre un générique ?)

Non (D’accord)

(Vous en avez entendu parler ?)

Oui disons que le médecin quand il parle il a un générique bon moi j’ai rien contre… le pharmacien quand on veut un médicament il dit écouter il existe un autre c’est un générique euh vous êtes d’accord j’ai rien contre

(D’accord)

Il reste que mon fils il a dû prendre des antibiotiques il avait pris la toux et puis j’ai été pour lui à la pharmacie et là la pharmacie m’a dit écoutez il existe un générique j’ai dit oui pas de problème puisque qu’il a la même chose que moi pour les médicaments

(D’accord, ok. Donc là le pharmacien vous a proposé en tout cas une fois voir d’autres de modifier la…)

Oh oui ça les pharmaciens …

(Ils sont attentifs à ça ?)

Oui oui (D’accord)

(Et puis, vous avez entendu parler par les médias par exemple, les journaux, la télé ?)

Il y a des articles mais disons que ce n’est pas ce qui m’intéressait le plus.

(D’accord, donc vous ne les lisez pas ?)

non ni sur internet entre parenthèses

(D’accord, ok. Mais vous allez sur internet pour des problèmes de santé ?)

Moi j’ai été voir sur internet pour les problèmes de dos et puis j’ai été voir une dernière fois c’est quand on a reçu le dernier euh comme quoi il marquait qu’il y avait un angiome volumineux….j’sais pas ce que c’était cette histoire d’angiome j’ai regardé et puis j’ai vu que c’était des tâches de la peau des tâches machin et tout… puis j’ai regardé ce que c’était c’est dans l’os quoi exactement ce que sais je sais pas

(C’est une modification des petits vaisseaux, c’est tout à fait bénin.)

(Quand vous allez sur internet, ça m’intéresse, par exemple l’angiome vous avez tapé, comment avez-vous été chercher ?)

Ben déjà je savais pas comme ça s’écrivait alors j’ai pris le rapport puis j’ai marqué uniquement angiome

(Sur Google par exemple ?)

Oui sur Google ça comme toute une catégorie

(Vous ouvrez comment les sites ? Les uns après les autres, vous vous souvenez ?)

Bon ben on se lasse au bout d’un moment parce qu’on cherche un truc qui est pour nous mais bon y’a sur la peau sur les bébés sur les femmes enceintes sur ouhlala

(Oui, ça peut être différentes parties du corps les angiomes. Ca vous est arrivé d’aller chercher des informations sur internet sur les médicaments ?)

Non absolument pas

(D’accord. Vous lisez la notice ?)

Non jamais

(Non ? D’accord.)

Ma femme les lit mais ça j’sais pas ces notices c’est déjà écrit tout petit et puis c’est plus un truc féminin que masculin

(D’accord. Vous vous renseignez comment sur les médicaments ?)

J’me renseigne pas

(Non ?)

Non

(Le médecin vous informe ?)

Oui oui

(D’accord. Puis ça vous suffit ?)

Ca me suffit puis dés fois ça rentre par une oreille et ça ressort par une autre

(Le pharmacien vous donne des informations ?)

Oui oui le pharmacien bon et puis c’est lui qui note toutes les doses

(Oui, tout à fait, d’accord. Vous vous souvenez qu’il y avait eu une grande campagne publicitaire d’affichage sur les génériques ?)

Non absolument pas

(Non. D’accord)

(Donc en fait, votre médecin quand il y a des génériques il vous les prescrit ?)

Oui oui

(Ok)

(Et le pharmacien ?)

Aussi

(Et il change parfois et vous, vous lui faites confiance ?)

Tout à fait

(Même si ce n’est pas ce qui est écrit sur l’ordonnance ?)

Tout à fait

(D’accord. Vous acceptez qu’il change, que le pharmacien change, d’accord.)

(Ok. Donc effectivement pour le Brufen que vous avez pris, il a y plusieurs génériques, vous vous souvenez d’avoir pris l’Irfen ou des génériques ?)

L’Irfen je crois que ça me dit quelque chose

(Ou Grefen ?)

J’sais pas j’me rappelle pas

(D’accord)

( Pour vous, c’était la même chose, les médicaments ? Bon ils n’ont pas eu beaucoup d’effets malheureusement chez vous mais, selon votre expérience, d’avoir eu l’original par exemple le Brufen et puis l’Irfen, vous n’avez pas eu plus de douleurs à l’estomac ou plus de problèmes ?)

Non avec les médicaments j’ai jamais eu disons euh des douleurs à l’estomac même ici au début quand on se fait opérer le lendemain quant ils enlèvent la morphine ils nous en donnent des médicaments ça je les ai toujours pris (oui) et puis au bout de deux ou trois jours là je demande aux infirmières qui regardent parce que j’aimerais arrêter les médicaments ils disent non les médecins ils conseillent de les prendre et tout puis je dis écoutez moi je veux essayer sans médicament ce qu’il y a de dommage c’est qu’ils les déballent (ah oui, donc ils sont perdus entre guillemets) mais y a plusieurs qui étaient à côté de moi et ils tiraient la table de nuit…bon ben j’dis que c’est une bonne chose si’ls peuvent arrêter les médicaments

(Ok. Si je me souviens bien, vous vous n’êtes plutôt pas pour les médicaments ?)

Non pas du tout

(Vous avez eu de mauvaises expériences avec les médicaments ?)

Non c’est tout simplement……c’est tellement rare que j’ai été malade donc quand on a des petits rhumes des petites grippes on s’fait un thé citron un thé rhum et un vin chaud et puis le wk on transpire bien comme il faut…le lundi on était tout bien

(Pour les douleurs c’est un petit peu plus difficile)

Ah ça au vin chaud ça passe pas.

**Patient 24**

(A votre avis, qu’est-ce qu’un générique ?)

En raccourci un médicament moins cher euh c’est censé être les mêmes molécules

(Censé ?)

Entre parenthèses

(Entre parenthèses, vous n’y croyez pas trop ?)

Moi j’ai pas vraiment de souci par rapport à ça…j’ai ma sœur qui a été greffée des reins y’a une cyclosporine ou j’sais pas quoi qui ont été euh enfin de synthèse non j’sais pas comment ça s’appelle (de marque ?) voilà et puis y a eu le générique et y a eu enfin d’après ce qu’elle me raconte moi j’vous dis que ce je raconte je sais rien du tout là-dessus j’suis pas pharmacien y a eu des gros problèmes… ma mère qui souffre un peu d’hypertension a pris un générique soi-disant exactement le même et c’est reparti à la hausse alors ça c’est que des trucs que j’ai entendu mais que j’ai pas testé

(D’accord. Pour vous, est-ce-que c’est toujours aussi efficace que l’original ?)

Dans ce que j’ai testé moi ? (oui) euh j’ai pas fait de différence je dirais mais vue que c’est tout le temps variable (D’accord) moi j’ai des problèmes de tension artérielle qui paraît euh un jour je suis bien toujours en prenant mes médicaments régulièrement (oui) un jour j’suis bien un jour j’suis moins bien j’ai commencé avec un…enfin un d’usine quoi j’ai suivi avec des génériques (oui) je regarde la courbe presque la même ça n’a rien changé

(Là l’effet est le même. Les effets indésirables sont les mêmes ?)

J’en sais rien du tout

(D’accord, vous n’avez pas remarqué de… ?)

Non

(D’accord. Et puis votre sœur et votre mère ne vous ont pas dit les effets indésirables ?)

Je n’ai pas de renseignements là-dessus

(Vous ne savez pas, d’accord)

Non non

(Vous pensez que les génériques sont soumis aux mêmes contrôles que les médicaments originaux ?)

J’espère que oui…j’imagine que oui

(D’accord. Vous savez comment un médicament est mis sur le marché ?)

Euh non pas vraiment

(Quel genre de contrôle ?)

J’imagine enfin j’imagine qu’il y a des tests quoi enfin ouais

(D’accord. Si vous êtes d’accord je vais rien dire je reprendrai après ?)

Oui

(D’accord, ok. A votre avis est-ce-que le générique est plus difficile à avaler que l’original ?)

Non

(Vous n’avez pas remarqué, pour vous ce n’était pas un problème ?)

Non c’est vrai qu’effectivement ils ont l’air un peu moins lisses mais je parle de ceux que j’utilise moi mais non ça ne me pose pas de problèmes….en moyenne j’en mange une poignée de cinq ou six par jour ça descend d’un coup

(Ok. Vous savez qui fabrique les génériques ?)

Pfffou euh je pense que c’est les usines pharmaceutiques quoi

(D’accord. Les mêmes que…)

Oh peut-être pas les mêmes parce qu’il y a pas de raisons qu’ils montent pas leur truc hyper cher contre un truc un peu moins cher…mais d’autres usines enfin

(D’autres usines, d’accord.)

(Vous n’avez pas hésité quand on vous en a proposé ?)

Non non je trouve que les pharmaciens sont assez riches

(D’accord.)

Même trop riche

(Ok)

(Comment en avez-vous entendu parler ?)

Information télé journaux toubibs

(D’accord. La télé vous vous souvenez du genre ?)

Non

(Vous vous souvenez en avoir entendu parler mais vous ne vous souvenez pas de quoi ?)

Non pas vraiment

(Et les journaux ?)

Pas plus que ça non plus

(D’accord)

C’est le flou enfin je sais que j’ai été informé mais je sais pas vraiment par quel canal

(Vous savez un peu quel genre d’informations vous avez reçu ? Ca portait sur quoi ?)

J’crois la qualité enfin…ils nous garantissaient que c’était les mêmes molécules euh puis que le prix était quand même nettement inférieur…dans les grandes lignes

(D’accord. Ca vous l’avez bien retenu. Ok. Vous vous souvenez des campagnes publicitaires dans les affichages dans la ville ?)

Non pas du tout

(Non ? Il y a quelques années qu’il y a eu ça ?)

J’y suis très peu en ville je reste dans mon trou

(Avez-vous été chercher des informations à propos des génériques sur internet ?)

Non j’suis pas internet

(Pas du tout ?)

Non

(Jamais été chercher pour le mal de dos sur internet ou comme ça ?)

J’ai pas internet

(Ah vous n’avez pas internet, d’accord.)

(Votre médecin vous en a-t-il déjà parlé ou prescrit?)

Dans quel sens ?

(Est-ce-qu’il vous en a prescrit ?)

Oui oui

(Est-ce-qu’à cette occasion il vous en a parlé ?)

Oui

(Vous vous souvenez de ce qu’il vous en a dit, à propos des génériques ?)

Pas pas grand-chose enfin oui il m’a dit enfin ce que je crois savoir déjà que c’était moins cher et que c’était les mêmes molécules et puis je pense qu’il pensait un peu comme moi qu’il était pas là pour enrichir la chimie bâloise

(D’accord. Et quand il vous les a proposés, vous avez accepté ?)

Sans souci oui

(Sans souci ? D’accord.)

( Votre pharmacien vous a-t-il déjà proposé de substituer le médicament original pour un générique ?)

Peut-être j’ai pas vraiment de souvenirs peut-être qu’on m’en a parlé

(D’accord. Si c’est le pharmacien qui propose, vous acceptez de changer ?)

Oui

(Même si le médecin n’en a pas parlé avant ?)

ah peut-être pas j’entends à la rigueur s’il me dit c’est le même j’entends j’fais la même démarche qu’avec le médecin j’ai confiance aux formations ainsi qu’aux médecins

(D’accord. Ok)

Mais je pense que je lui en parlerai peut-être

(Vous parlez d’abord au médecin, avant de changer ?)

Voilà si je suis sûre que c’est la comment….untel pour untel s’il est vraiment s’il est vraiment l’équivalence oui…le pharmacien me ferait peut-être pas changer de médicament que le médecin m’aurait donné ou quelque chose d’autre je dirai

(Donc si vous venez à la pharmacie avec une prescription faite par le médecin et que le pharmacien vous dit…)

J’ai le générique de ça

(J’ai le générique de ça, vous le prenez ?)

Oui

(D’accord. Même si ce n’est pas le même nom ?)

Non non moi je m’en fous un peu

(D’accord. Le nom n’a pas d’importance ?)

Aucune vu que je sais pas ce qu’il y a dedans alors sachant qu’ils peuvent appeler ça comme ils veulent

(Vous regardez quand vous recevez la boite de médicament, vous regardez ce qu’il y a dedans, vous relisez la notice ?)

La notice oui oui en général ça déprime mais il faut

(Ca déprime pourquoi ?)

Ben tous les effets secondaires ouais…j’aime déjà pas trop les médicaments alors je la lis pas toutes les fois que j’ouvre une nouvelle boite

(D’accord. Vous la lisez si vous ressentez quelque chose ou pas vraiment ?)

pas vraiment oui c’est vrai que la dernière fois on a testé d’autres médicaments dont je me rappelle pas le nom avec mon médecin…ça me foutait des nausées des envies de vomir enfin de tournis j’étais zombie là j’ai un peu lu quand même et puis ben j’ai arrêté de les prendre quoi et je lui en ai parlé

(Donc actuellement pour les douleurs, je reprends votre lettre. Donc, vous avez du Dafalgan, du Sirdalud, vous savez s’il y a des génériques pour ce médicament ?)

Je sais pas

(Vous savez pas. Donc on vous les a pas proposés ?)

Non parce que j’ai d’autres médicaments je sais que c’est des génériques alors je pense que….

(La pravastatine ?)

Oui

(C’est le générique, tout à fait.)

Et puis je sais plus euh

(L’Exforge, l’Exorge ? et puis vous avez l’aspirine cardio aussi, c’est que j’ai marqué.)

oui

(D’accord. Parce que par exemple pour le Dafalgan, y a des génériques.)

Ah

(Le Dafalgan est déjà un générique mais je ne suis pas sûre que ce soit absolument le meilleur marché, mais on ne vous a pas proposé autre chose pour ça ?)

Non je savais même pas

(D’accord. Qu’est-ce que vous aimeriez avoir par exemple si le médecin vous prescrit un générique, pour vous quelle est l’information qui est importante d’avoir de la part du médecin ?)

Si ça va me soulager j’entends comme n’importe quel autre médicament que ce soit un générique ou pas…à partir de là j’ai pas besoin d’autres informations

(D’accord. Donc, vous n’attendez pas une information particulière sur le générique de la part du médecin ?)

Non j’attends une information sur la qualité du médicament et qu’est-ce-qu’il va cibler mais au-delà…

(Au-delà, d’accord. Et puis si le pharmacien substitue le médicament, vous venez avec une ordonnance et il vous dit ah mais là j’ai le générique, qu’est-ce-que vous aimeriez avoir comme information de la part du pharmacien à propos de ce générique ?)

Si c’est la même molécule je chercherai pas d’autres informations j’entends pour autant que ce soit un médicament que j’ai testé que j’ai déjà pris quoi…sur un nouveau médicament je sais pas vraiment

(Donc sur un nouveau médicament…)

Si c’est sur une nouvelle prescription…j’entends…j’aimerai être informé déjà par mon médecin après si on me dit c’est un générique j’y vais quand même

(D’accord. Donc vous acceptez la substitution du pharmacien si c’est un médicament que vous connaissez en fait ?)

Voilà

(D’accord. Mais si c’est une toute première fois, vous préférez prendre ce qu’à écrit le médecin ?)

Voilà

(D’accord.)

Mais il peut m’avoir mis un générique d’entrée je pense si ça vient de lui ça me gêne pas du tout

(Tout à fait. Le générique en soi vous gêne pas…)

Non

(Mais vous aimez en tout cas débuter un traitement avec ce qu’à prescrit le médecin, c’est juste ?)

Oui

(D’accord, ok.)

Parce qu’après j’me dis si j’ai des effets secondaires après ça peut embrouiller j’dis c’est un générique c’est peut-être le générique qui n’est pas terrible mais ça s’arrête à ça

(Oui, tout à fait, d’accord.)

(Donc vous avez une expérience des génériques comme la pravastatine. Contre la douleur, est-ce-que vous avez eu une expérience avec des génériques ?)

Peut-être bien oui je sais plus j’en ai tellement essayé je sais plus trop…parce que là je vous ai mis un petit peu ce que je me souvenais mais j’ai dû essayer la moitié de votre liste

(Parce que le Brufen par exemple….)

Ca j’ai pris

(Ca le Brufen, c’est le nom de marque mais y a beaucoup de génériques du Brufen : Irfen, Grefen…. ?)

Ca ne me dit rien non

(Ca vous dit rien ?)

Non

(Par exemple le Ponstan, ça vous l’avez mis et y a comme un nom de générique le Méfénacide, non ?)

Ca ne me dit rien du tout

(Ca vous dit rien non plus, euh le Tramal , y a des génériques comme le tramadol, elvepharm, tramadol mefa ?)

Peut-être….ça fait très très longtemps que j’en ai pas….Tramal ça date d’il y a très très longtemps

(D’accord. Et vous vous souvenez si ça avait été la même chose, la forme générique ?)

J’entends le Tramal je suis pas resté très très longtemps parce que ça c’est quelque chose qui me tuait complètement

(D’accord.)

J’ai peut-être des réactions bizarres par rapport à certains médicaments mais y a des trucs ça me couche alors j’aime pas du tout

(Autrement les autres médicaments que vous me signalez là, y a pas de génériques. Le Voltarène, y a des génériques du voltarène : diclofénac)

J’ai aussi arrêté le Voltarène parce que c’est une histoire on sait pas si elle est vraie ou si elle est pas vrai quand je me suis explosé le genou les ligaments on faisait des injections de Voltarène et quand je suis sorti de la clinique c’est là où mes problèmes d’hypertension ont explosé et y a quelqu’un qui m’avait fait une sorte de rapport avec le…

(Ca peut, ça peut…)

C’est extrêmement rare on m’a dit mais que ça pouvait avoir déclenché…

(Ca peut disons être mis en évidence, quelque chose qui existait…)

Alors à partir de là non avant j’étais bien on avait fait les examens avant les opérations j’étais nickel et quelques semaines quelques mois après c’était parti un peu en délire et tout d’un coup y avait eu une histoires avec le Voltarène donc depuis là j’y touche plus vraiment

(D’accord, ok. Bon autrement il n’y a pas d’autres génériques. Donc c’est en tout cas pas récent celles que vous auriez pu faire avec le tramadol, vous ne vous souvenez pas tellement en faite ?)

Non

(Ok. Est-ce-qu’il y a quelque chose que vous aimeriez ajouter à propos des génériques ?)

non rien de particulier ils ne me font pas plus peur que ça

(D’accord, oui, ça j’ai compris. Je vous remercie.)

**Patient 25**

(A votre avis, qu’est-ce qu’un générique ?)

Ben c’est quelque chose qui est un essai… c’est pour les essais sur les personnes pour voir si un médicament il peut agir ou pas comme les autres, moi c’est ce que je pense…

(C’est un essai ??) Ben je veux dire n’importe quel médecin chimiste… je vois ça comme ça… il doit essayer… je ne sais pas… mélanger les choses…et dire bon, mais ça ça doit donner… par exemple moi j’ai pris chez le Dr Ong mon médecin traitant… d’ailleurs je l’ai demandé, un journal… qui disait que la cannelle… ça peut être intoxicant, ça peut intoxiquer quelqu’un et certains médicaments… d’ailleurs je l’ai ce livre et je l’ai gardé parce qu’il m’a dit ‘vous êtes trop curieuse, je vais vous le donner’…. Beaucoup de choses… le kiwi… des choses comme ça… que c’est très… des fois c’est offensif pour la douleur, pour beaucoup de choses, intoxicant… Mais moi le médicament, sincèrement… l’autre jour à la pharmacie elle voulait me les donner… les génériques… moi je lui ai dit vous pouvez me les donner, moi si j’aime je prends si je n’aime pas je prends pas… elle m’a dit alors vous n’aimez pas, j’ai dit ‘écoutez, je ne sais rien’… au Portugal ça se fait beaucoup, ici ça commence à se faire, maintenant je n’en sais rien… Je l’ai pris mais sincèrement je l’ai pas pris, j’ai pas essayé encore… en fait c’est le… pour aller aux toilettes tous les jours… le XXX je sais pas comment… elle m’a donné… le YYY… je sais pas ce que c’est ! et pis après elle m’a dit ‘ça c’est exactement la même chose, mais comme on n’a pas en stock…’, ils ont téléphoné à 2 ou 3 pharmacies, ils n’avaient pas non plus… ben, j’ai dit ‘écoutez, donnez-moi… je vais essayer… mais sincèrement j’ai pas essayé…

(Qu’est-ce qui vous fait hésiter?)

Parce qu’en fait j’ai confiance, j’ai confiance au médecin et à ce qu’il me donne le médecin…par exemple le médecin ils m’ont donné le… pour les douleurs… je sais pas le nom mais…que vous-mêmes vous m’avez dit non ne prenez pas 60, prenez 30… pour les douleurs d’ici, le bras tout ça et moi sincèrement comme j’étais tellement mal un jour, j’ai tout arrêté… mais ça ne va pas mieux… ça ne va pas mieux ! Le docteur il m’engole parce que je devrais prendre le médicament qu’il me donne… mais ça ne va pas mieux !... je commence, j’essaie, j’arrête… voilà… alors c’est possible qu’avec les génériques, j’arrive à… si on m’explique bien peut-être que j’arrive à mieux comprendre qu’est-ce que c’est le générique…

(Même substance ?) Si, bien sûr ! bien sûr !

(Même efficacité ?) Non ! ( ?) Le problème c’est que quand on s’habitue avec un médicament, par exemple moi, ce qui me sauve c’est le co-Dafalgan… mais je suis à 4 par jour… et la douleur la nuit, elle persiste… et moi j’ai mal au bras, j’ai mal partout… et je dis mais pourquoi je prends pas un avant de me coucher ? parce que c’est trop ! Il est vrai que s’il y avait un autre qui pouvait remplacer le co-Dafalgan… peut-être ce serait une bonne chose… mais qui soit assez fort pour qu’il m’élimine la douleur…

(Dedans la même chose… ?) je pense c’est fait par les plantes….

(Donc tout pareil dedans ?) je pense quand même… !?

(Si on compare original et générique, dedans idem ?) je sais pas… je connais rien en biologie… mais je sais pas… la science est tellement avancée… que c’est possible que les génériques fassent le même effet que les autres… maintenant, si vous me demandez ‘est-ce que vous pensez que vous êtes guérie ?’, moi je vous dirai ‘non’ !

(même substance –pas même efficacité ?) oui, et vous savez, pour une raison très simple… parce que vous savez, je suis habituée au co-Dafalgan et quand je prends… ils m’ont donné xxx c’est une autre marque… après la bleue ( ??) tout ça moi je dis mais je comprends pas parce que ça ça m’enlève la douleur à la longue et le co-Dafalgan c’est boum une demi-heure après j’ai rien par contre 2 heures après c’est fini, la douleur elle remonte… c’est vrai s’il y avait quelque chose dans les génériques pour mes douleurs, moi je serais la première à prendre… et je peux même faire l’essai !...

(Générique pour intestin – on vous a dit même que original ?) et moi j’attends les autres parce que je suis habituée avec l’autre…

(pas la même chose ?) il faut connaître la substance, qu’est-ce qu’il y a dedans… si c’est la même chose, ben c’est ça….

(Substance aussi dans générique - même ? différente ?) ah ben non parce que sinon pourquoi ils ont le mis le générique !!? qu’est-ce que ça veut dire le générique ??

(donc 2 médicaments un peu différents ?) pas la même substance… pi pas la même puissance peut-être, je sais pas…

(c’est un autre ?) Tout à fait ! par exemple, moi le Dafalgan, en pastille qu’on avale avec de l’eau, ça me fait mal à l’estomac, ça me fait beaucoup de choses, tandis que le co-daflagan que je fais dans l’eau, il me fait pas de mal… c’est dans ma tête ?!

(Pas mêmes effets indésirables ?) ça je ne sais pas…

(Copie - photocopie ?) moi je dis que c’est plutôt une photocopie… moi j’ai la vraie photo de mon papa et de ma maman et de mes enfants… mais quand je fais une photocopie, c’est pas pareil ! La vraie photo, c’est une chose… je suis peut-être méchante, mais c’est la vérité… ! … c’est pas aussi bien que la vraie

(votre MD vous a prescrit génériques ?) Non. Non non non… et de toutes façons, il sait que je suis têtue… il m’engole chaque fois que je fais pas les choses comme il faut… mais en fait, moi je veux bien essayer… mon problème ici (joue) par exemple des fois le matin je me lève très bien, et après je m’énerve pendant la journée et la douleur tac ! sinon, je me lève déjà avec l’œil sec, très douloureux, comme en bois à l’intérieur, je masse un tout petit peu… et petit à petit ça se réveille, donc il doit y avoir une solution pour ça… Quand je masse gentiment c’est mieux… parce que c’est trop sec ici… on dirait en bois là-derrière… je asse gentiment et je lave le visage, et je mouille, mais 2 heures après, la douleur est là, donc il faut trouver une solution ! moi je suis pour !

(pas de génériques par MD, no pour douleur ni autre ?) Je crois pas

(Proposition par pharmacien ?) Oui et moi j’ai dit tu restes bien là (dans l’armoire) et tant que je m’en passe, tant mieux… sinon je suis obligée… de commencer…

(Inquiétudes par rapport générique ?)…. Ben… moi je connais pas grand-chose des plantes… mais je sais qu’il y a certaines choses comme par exemple… soi-disant, parce que moi j’ai jamais essayé, le gros cigare ( ??) le vrai vrai cigare, il paraît que ça enlève… certaines douleurs… moi je sais pas, ça c’est une plante, moi je la connais pas… peut-être que cette plante, mélangée avec quelque chose, ça va aider… je me demande si ça c’est générique ou pas… mais je ne sais pas… parce que les génériques en fait c’est pas un bout de plastique, c’est une plante quand même…

(qui les fabrique ?) c’est de toutes façons le pharmacien… le biologiste… (même contrôle ?) oui je pense… quand même… c’est important… on peut pas donner un médicament à n’importe qui… voilà… ‘tu prends ça et pi c’est bon’, non ! quand même ! il doit y avoir un contrôle très précis mais bon, dans le médicament il y a toujours des erreurs… et des avantages, ça c’est normal…

(Erreurs et avantages ?) ben voyez en France les médecins qu’ils ont fait des bêtises avec le… le médicament pour le cœur, eh ben c’est interdit même de le prendre, c’est fini, on les enlève du marché… et c’est pourquoi… parce que je sais pas d’où ça vient, les gens, nous les malades, on sait pas d’où ça vient… c’est confié ça, c’et confié au médecin… même pas au médecin traitant parce que le médecin traitant il ouvre son livre pi il dit ben vous avez le diabète, vous pouvez prendre ça ça et ça… et sinon si vous ne l’avez pas vous pouvez prendre un autre, enfin moi je vois les choses comme ça… c’est pour ça que moi je trouve que… il faut faire confiance des fois … aux intelligents quoi !... aux gens capables…

(Si MD prescrivait générique ?) ça dépend pour quoi faire, oui… oui, bien sûr… si c’est mon médicament… mon médecin traitant ou… ou… plus… l’hôpital, quelqu’un que j’ai vraiment confiance, parce que moi j’ai besoin d’un traitement… j’ai besoin d’un traitement ! d’ailleurs il fait qu’on me fasse un scanner tout entier. Il y a quelque chose chez moi !... mais quoi je ne sais pas, je ne sais pas… parce que les douleurs… écoutez ! on me dit que là (bras) j’ai rien et j’ai toujours… ça m’embête, ça me prend jusqu’au doigt tout tout tout… alors quelque chose il y a… mon médecin il me dit que c’est à cause des nerfs, je sais pas quoi… entre-temps j’aimerais bien trouver un médicament pour ça !

[XXX décrit ses douleurs et sensations au niveau de la bouche et de la joue] Bien sûr un traitement générique, pourquoi pas ?

(Si prescrivait ? confiance dans efficacité ?) Bon… ! L’eau qui vient du ciel, c’est l’eau que la terre elle circule, tatata, tout ce baratin… ce n’est pas de l’eau filtrée et que c’est l’eau que on peut le boire sans le bouillir… et si c’est de l’eau du robinet pour un enfant, par exemple, on ne peut pas lui donner de lui qui n’est pas bouillie… alors, il y a quelque chose ! pour tout, il y a une solution, mais simplement… il faut la trouver quoi ! Par exemple moi je suis pas trop pour boire de l’eau de bouteille qui est de l’eau mise dans une bouteille, quand l’eau du robinet si elle est bien désinfectée, avec n’importe quoi ! si elle est bien désinfectée, elle est plus pure, plus propre que celui-là (montre bouteille sur le bureau)… moi je suis pas une personne intelligente à ce point de savoir beaucoup de choses, mais c’est vrai que l’eau de la pluie, on peut utiliser pour tout…

(Vous vous posez des questions?) je me pose des questions à la nature… on sait que les plantes il y en a qui sont très bonnes pour la santé, on sait qu’il y a des plantes qui sont très mauvaises pour la santé, et nous… disons… certaines fleurs, on a des fleurs à la maison, et ils sont agressives pour nous, pour la peau, pour le corps, pour le cerveau, on doit pas les avoir à la maison et peut-être que cette plante elle va me faire un bon médicament pour que je guéris de ça de ça ou de ça. Pour ça, il faut connaître.

( ?) moi mon médicament, privilège, c’est le co-Dafalgan… et les piqûres automatiquement, tzzzt, ça passe la douleur pour un bon moment et pi c’est bon… j’ai demandé au docteur Ong qu’il me le fasse et il veut pas me le faire. Il me l’a fait de ce côté et c’est tip-top, je suis tip-top (épaule droite). C’est lui qui save, c’est pas moi. Bien sûr que la piqûre il va agir [xxx morphine] et pi il va agir comme il faut, sur la partie que j’ai mal… et si c’est un médicament, petit à petit, petit à petit, c’est plus long, je ne sais pas, je pense comme ça moi. [xxx piqûre a guéri côté droit]

(Avez-vous été chercher des informations à propos des génériques sur internet ?)

(Pour vous renseigner ?) Je peux voir si vous avez quelque chose qui peut me montrer plus ou moins qu’est-ce que c’est, si c’est des fruits naturels, si c’est des médicaments naturels, qu’est-ce que c’est… par exemple les médicaments chinois, il paraît que c’est très bien et que c’est plus de naturel qu’autre chose… mais à savoir ??... moi j’ai une amie… une amie… elle est médecin, elle me dit ‘Fatima, fais la thérapie chinoise, tu verras que tu va guérir’… mais moi je suis liée à l’hôpital, je veux continuer…

(renseignements ?) a pris rendez-vous auprès du chirurgien qui l’avait opérée de sa tumeur à la joue. Avait pris des médicaments, ne pense pas qu’il s’agissait de génériques.

(nom ‘générique’ ?) bonne question… moi je suis pas assez intelligente pour vous répondre… ( ?) ça peut être… gestion… une manière de gérer, comme par exemple, il y a plus cette plante là ou celle-là, on va essayer avec l’autre, ça va faire la même chose… c’est une façon de dirigir les choses, même pas l’économie mais pourquoi pas l’économie…parce que vous êtes d’accord, s’il y a pas de pommes-de-terre au Portugal, ils viennent les chercher en Suisse, le Portugal est servi avec les pommes-de-terre suisses, pourquoi pas ? ça c’est une façon d’essayer de diriger mieux les choses, manque de plantes, manque de… je ne sais pas, je pense…

(commentaires supplémentaires) Je connais quelqu’un, Monsieur O, il habite à B… c’est un médecin, un médecin-biologiste, bon tout ça, ils ont essayé avec un Australien de faire des médicaments pour le sida, il a eu de très bons résultats, mais ils étaient pas accordés avec les autres... enfin les gens de droit de faire ça, alors tout est stoppé pour le moment et pourtant tout le monde a donné un peu, un peu de l’argent, un peu de l’argent, un peu de l’argent, ça c’est vrai, je sais, c’est des gens que je connais bien. Alors je pense que le médicament, les génériques, c’est la même chose, si on n’a pas l’ordre de faire ça ça ça, on peut pas le vendre, on peut pas le proposer aux gens, et voilà, c’est comme ça.

( ?) comme le parfum, quand vous allez chez XX, ils font des essais avec le parfum… il y a le vrai, il y a le faux ( ?) moi je suis pas capable (de les distinguer) il y a que les gens diplômés qui savent s’il est bon ou s’il est pas le même, exactement le même, la bonne qualité. Il faut des gens diplômés pour ça. C’est comme la crème pour laver les dents, il y a mon José, il me dit ‘non, je veux que ça’ et moi je vous dit que la crème pour laver les dents , s’il y a le fluor, n’importe lequel il est bon, pas besoin de payer 10 francs pour un truc pour laver les dents, mais non, il dit que non, il faut que ce soit que ça… ça c’est dans la tête, c’est comme moi, moi je suis comme ça
