# Supplementary material for: Prescribing Generic Medication in Chronic Musculoskeletal Pain Patients: An Issue of Representations, Trust, and Experience in a Swiss Cohort
Source: PLoS One. 2015 Aug 3;10(8):e0134661. doi: 10.1371/journal.pone.0134661 (PMC4523195; doi:10.1371/journal.pone.0134661)
Supplement: S1 Table — (DOCX) [file pone.0134661.s002.docx]

**Prescribing generic medication in chronic musculoskeletal pain patients: an issue of representations, trust, and experience in a Swiss cohort**

**S1 Table. Sociodemographic, pain and medication intake characteristics of the patients**

| Patients number | 1 | 2 | 3 | 4 | 5 | 6 | 7 | 8 | 9 | 10 | 11 | 12 | 13 | 14 | 15 | 16 | 17 | 18 | 19 | 20 | 21 | 22 | 23 | 24 | 25 |
| --- | --- | --- | --- | --- | --- | --- | --- | --- | --- | --- | --- | --- | --- | --- | --- | --- | --- | --- | --- | --- | --- | --- | --- | --- | --- |
| gender | m | m | f | m | m | m | f | f | f | f | f | m | f | m | m | m | f | f | m | f | f | m | m | m | f |
| age | 51 | 39 | 39 | 55 | 43 | 78 | 62 | 42 | 60 | 58 | 54 | 43 | 54 | 48 | 53 | 72 | 43 | 40 | 38 | 35 | 50 | 40 | 59 | 57 | 60 |
| Education level | 4 | 3 | 1 | 1 | 1 | 4 | 2 | 3 | 3 | 2 | 1 | 4 | 2 | 2 | 2 | 4 | 1 | 1 | 1 | 1 | 2 | 3 | 2 | 3 | 2 |
| Employment | 6 | 1 | 6 | 2 | 6 | 3 | 6 | 5 | 5 | 2 | 2 | 2 | 2 | 2 | 6 | 2 | 2 | 5 | 5 | 5 | 6 | 4 | 5 | 5 | 5 |
| pain | ms | ms | lbp | lbp | lbp | lbp | ms | fm | ms | fm | lbp | ms | fm | lbp | ms | ms | ms | fm | lbp | lbp | ms | ms | lbp | lbp | ms |
| VAS | 10 | 50 | 100 | 90 | 60 | 60 | 50 | 80 | 90 | 100 | 80 | 80 | 90 | 50 | 60 | 80 | 70 | 80 | 70 | 100 | 60 | 20 | 50 | 60 | 10 |
| duration | 50 | 9 | 5 | 10 | 8.5 | 18 | 18 | 13 | 15 | 1.5 | 30 | 20 | 10 | 3 | 15 | 3 | 1 | 20 | 2 | 3 | 8 | 8 | 3 | 8 | 1 |
| NSAIDs |  |  |  | x | x |  |  | x |  | x |  | x | x |  | x | x |  | x | x | x | x |  |  |  |  |
| Paracetamol |  | x | x |  | x |  |  |  |  |  | x | x | x | x |  |  |  |  |  | x | x |  |  | x | x |
| Weak opioid | x | x | x |  |  | x |  |  |  | x |  | x | x | x |  |  |  |  | x |  |  |  |  |  | x |
| Strong opioid |  |  |  |  |  |  | x | x | x |  | x |  |  |  | x | x | x |  |  |  |  |  |  | x |  |
| Antidepressant |  |  | x | x | x |  | x | x |  | x | x |  | x |  | x | x | x | x | x | x | x |  |  |  |  |
| Anticonvulsivant |  |  |  |  | x | x | x | x | x | x |  |  |  |  | x |  |  |  | x |  |  |  |  |  |  |
| Myorelaxant |  |  |  |  |  |  |  |  |  | x |  |  |  |  |  |  |  | x | x | x |  |  |  | x |  |
| Non analgesic medication | x |  |  | x | x | x |  |  | x | x |  |  | x |  | x | x |  |  |  | x |  |  |  | x | x |
| Generic analgesic | x | x | x |  | x | x |  | x | x |  | x | x | x | x | x |  | x | x | x | x | x | x | x | x |  |
| Generic other | x | x |  | x |  | x |  | x | x | x | x |  | x |  |  | x |  |  |  | x |  |  |  | x | x |
